# When Fiction Is Just as Real as Fact: No Differences in Reading Behavior between Stories Believed to be Based on True or Fictional Events

**DOI:** 10.3389/fpsyg.2017.01618

**Published:** 2017-09-20

**Authors:** Franziska Hartung, Peter Withers, Peter Hagoort, Roel M. Willems

**Affiliations:** ^1^Neurobiology of Language, Donders Institute for Brain, Cognition, and Behaviour, Radboud University Nijmegen, Netherlands; ^2^Neurobiology of Language, Max Planck Institute for Psycholinguistics Nijmegen, Netherlands; ^3^Faculty of Arts, Centre for Language Studies, Radboud University Nijmegen, Netherlands

**Keywords:** narrative, reading, language comprehension, narrative perspective, narrative engagement

## Abstract

Experiments have shown that compared to fictional texts, readers read factual texts faster and have better memory for described situations. Reading fictional texts on the other hand seems to improve memory for exact wordings and expressions. Most of these studies used a “newspaper” vs. “literature” comparison. In the present study, we investigated the effect of reader's expectation to whether information is true or fictional with a subtler manipulation by labeling short stories as either based on true or fictional events. In addition, we tested whether narrative perspective or individual preference in perspective taking affects reading true or fictional stories differently. In an online experiment, participants (final *N* = 1,742) read one story which was introduced as based on true events or as fictional (factor *fictionality*). The story could be narrated in either 1st or 3rd person perspective (factor *perspective*). We measured immersion in and appreciation of the story, perspective taking, as well as memory for events. We found no evidence that knowing a story is fictional or based on true events influences reading behavior or experiential aspects of reading. We suggest that it is not whether a story is true or fictional, but rather expectations toward certain reading situations (e.g., reading newspaper or literature) which affect behavior by activating appropriate reading goals. Results further confirm that narrative perspective partially influences perspective taking and experiential aspects of reading.

## Introduction

The main goal of the present study investigated whether expectations regarding a story being based on true or fictional events alone can drive differences in reading behavior and affective responses during reading. Research on reading behavior and comprehension shows that reading behavior can be affected by whether readers believe a text to be factual or fictional. For instance, Zwaan (1994) showed that knowing that a text was taken from a newspaper (factual) or from a novel (fictional) influences reading behavior and memory. In two studies he reported that texts labeled as factual were read faster compared to fictional texts (see also Wolfe, 2005; Altmann et al., 2014). Moreover, readers showed better performance on situational memory for factual texts, but better memory for the text's surface structure for fictional texts (Zwaan, 1994). This means that readers better remembered what has happened and in which order for factual texts; while readers better remembered the exact wording of a story for fictional texts.

There is further evidence that the subjective intensity of negative valence is weaker for fictional than to factual information, even when arousal is the same for both (Sperduti, 2016). Other studies suggest on the contrary that there is no difference in emotional response to fact and fiction (Goldstein, 2009), but rather that observed differences in emotional response to factual or fictional information are mediated by individual variation in how much participants scrutinize the information they are presented with (Green et al., 2006; Wolfe and Mienko, 2007). Some accounts argue that the expectation of reading fiction triggers a specific reading strategy which allows us to get immersed and experience strong emotions while engaging with fiction (Oatley, 1999b; Mar and Oatley, 2008). This would mean that readers would get more immersed, and experience stronger emotions in a fictional story compared to factual stories. Other accounts argue however, that it is not the knowledge about the factuality of a narrative, but rather an engaging narrative style which is causing readers to get more immersed and to experience stronger emotions, regardless of whether the information is believed to be true or not (van Krieken et al., 2015a,b). For instance van Krieken et al. (2015a) showed that when reading public reports about crimes, readers identify more with eyewitnesses and feel more present when reading a narrative eyewitness report than when reading a non-narrative report of the same event.

Processing of fictional and factual information also seems to be supported by different neural networks (Han et al., 2005; Mar et al., 2007; Abraham et al., 2008; Metz-Lutz et al., 2010; Altmann et al., 2014). For instance, Altmann et al. (2014) found evidence for different neural networks involved depending on whether the text was believed to be factual or fictional. The activation pattern while reading factual texts was associated with motor areas suggesting “an action-based […] reconstruction of what happened” in the story' (Altmann et al., 2014, p. 26). Reading fictional stories on the other hand was associated with networks linked to social cognition and imagining possible events suggesting “a constructive simulation of what might have happened” (ibid, p. 27).

From the reviewed research above it seems to be clear that readers apply different reading strategies when being presented with texts that are associated with different default expectations regarding their factuality. One problem in the current research however is, that all studies reporting effects of factuality manipulated factuality by telling readers that the texts are taken from newspapers or from novels. Because novels and newspaper articles differ on more dimensions than factuality, it might be the case that these effects are not driven by the expectation regarding whether the story is true or not, but rather by expectations regarding writing style and reading goals associated with these types of text and with situations in which people typically read them. Such effects may therefore be a particular instance of a more general effect of genre expectation and not driven by the factuality of the information.

We know that readers use prior knowledge about the genre to systematically select criteria and strategies for comprehension linked to different reading goals (van den Broek et al., 2001, 2005 for review; Zwaan, 1994). Reading goals for factual texts are typically to obtain information about reality (e.g., reading for study purposes or reading the news) and are thought to prompt reading strategies which emphasize connections in the text in order to reconstruct the contents (van den Broek et al., 2001). Reading for enjoyment on the other hand is associated with a stronger motivation for subjective experience and is linked to reduced scrutiny and attention to detail (Zwaan et al., 1995; van den Broek et al., 2001; Mar and Oatley, 2008). For example, when reading a route description, readers are more likely to focus on spatial relations and the order of reference points, whereas reading a landscape description in a novel is more likely to motivate self-relevant thoughts and associations, as well as aesthetic experiences.

Narratives, as compared to non-narrative texts, often cause the reader to get immersed into the story and construct multimodal situation models (Zwaan and van Oostendorp, 1993). Immersion is a state of cognitive, emotional, and imaginative absorption (Gerrig, 1993; Green and Brock, 2000; Green, 2004; Zwaan, 2004; Jacobs, 2015), which overlaps conceptually with flow (Csikszentmihalyi, 1990), and transportation (Sestir and Green, 2010). Such states of absorption are marked by “deep concentration, losing awareness of one's self, one's surroundings and track of time”(Kuijpers, 2014, p. 28; see also Csikszentmihalyi, 1990). Immersion is a multidimensional experience based on factors, whose contribution to the experience of being immersed varies with the situation (see also discussion in Hartung et al., 2016) and is positively linked to enjoyment (Green, 2004; Busselle and Bilandzic, 2009). The most important factors which contribute to immersion along a variety of narrative texts are the experience of mental imagery, emotional engagement with protagonists, transportation into the story world, and attention during reading (Kuijpers et al., 2014).

Readers can get immersed in a story by either taking the role of an observer (3rd person perspective) or by taking the viewpoint of one of the characters (1st person perspective) (Oatley, 1999a; Boyd, 2005; Sanford and Emmott, 2012). Readers often take the mental perspective of the protagonist and simulate his or her mental states as the point of view when constructing a situation model (O'Brien and Albrecht, 1992; Albrecht et al., 1995; Horton and Rapp, 2003). It has further been shown that with which character the viewpoint is aligned affects if readers take a 1st person perspective (de Graaf et al., 2012). Taking the viewpoint of a character is linked to identification. During the course of the story this results in experiencing emotions of empathy (Oatley, 1999a). While some accounts argue that fictional narratives in particular promote immersion during reading (Oatley, 1999b; Mar and Oatley, 2008; Jacobs, 2015), other accounts argue that the engaging style in which fiction is typically written is driving immersion and engagement during reading (van Krieken et al., 2015a).

Perspective is important in language comprehension especially for situation models. For example, when reading a novel, readers can get immersed as if they would experience the situations from the viewpoint of the protagonist or from the viewpoint of an eyewitness who is merely observing. Narrative techniques like focalization and the use of personal pronouns (*I* or *he/she*) or proper names referring to agents of actions are thought to guide perspective taking during comprehension and to influence how readers relate to characters in narratives (Gerrig, 1993). Indeed, recent studies show that whether the protagonist is referred to with 1st or 3rd person pronouns can influence perspective taking and experiential aspects of reading (Hartung et al., 2016) as well as spatial memory of events (Brunyé et al., 2009; Ditman et al., 2010). However, some recent studies suggest that these effects might better be explained by individual preferences for perspective and their interaction with the perspective in which the text is written (Brunyé et al., 2016; Hartung et al., 2017). In addition, Hartung and colleagues (Hartung et al., 2016; see also van Krieken et al., 2015b) showed that readers get more immersed when reading stories in 1st person perspective and that readers like these stories better as well.

It is unclear in how far the influence of narrative perspective on experiential aspects of reading is specific to fiction reading and its associated reading goals. While flexibility in perspective taking might be typical for reading fiction for pleasure and interacting with fictional characters, perspective taking might be less flexible when a real person is telling a story. In order to test whether flexible perspective taking is more strongly associated with reading goals specific for fiction reading, we take perspective taking as influenced by narrative perspective as one aspect of the experience of reading that is under investigation in the present study.

### Aim of the study

The goal of the present study was to test whether expectations about the factuality of a story alone can drive differences in reading behavior. We wanted to extend the research on how factuality expectations affect reading behavior by testing the effect of factuality expectation on reading behavior without potential confounding expectations associated with typical reading situations of certain types of text. In contrast to previous studies on readers' expectations regarding factuality (e.g., newspaper vs fiction), we decided for a different manipulation of factuality in order to keep the narrative character constant in both conditions. We investigated the effects of factuality expectation on experiential effects during reading and test whether processing of perspective is affected by reader's expectations about factuality of the stories. As a measure for experiential aspects of reading, we measured immersion into stories with the Story World Absorption Scale (SWAS) (Kuijpers et al., 2014) with its subscales *transportation, mental imagery, attention*, and *narrative engagement*. In addition, we included two items measuring perspective taking. Moreover, we measured liking of stories. We tested a large and diverse sample in an online experiment in which participants read a short story labeled as fictional or as based on true events and afterwards indicated how immersed they felt during reading and how much they liked the story. In addition, we tested memory of events in the story with a picture recognition task with pictures of events from 1st and 3rd person perspective. The overall goal was to test if perspective taking and experiential aspects of reading are influenced by reader's expectations about a text being based on true or fictional events and characters.

### Hypotheses

In line with previous research (Zwaan, 1994) we expected that stories labeled as based on true events are read faster and result in better memory performance in the picture recognition task compared to stories labeled as fictional. Moreover, in line with previous experimental research on narrative perspective, we expect that 1st person stories compared to 3rd person stories promote immersion, appreciation (Hartung et al., 2016), and mental imagery from the perspective of the protagonist (Brunyé et al., 2009). In line with Mar and Oatley (2008) we expected that people show stronger emotional engagement and are more likely to immerse with stories in 1st person (Zunshine, 2008). This effect might be more pronounced for fictional stories as suggested by accounts arguing for a stronger involvement in fictional narratives (Oatley, 1999b; Mar and Oatley, 2008). If the latter is the case this would show up as a statistically significant *fictionality* x *narrative perspective* interaction effect. This prediction assumes that a 1st person narration serves as an invitation to identify and immerse with the protagonist and might be specific to (certain types of) fiction (Gerrig, 1993; Oatley, 1999a; van Krieken et al., 2015a). In this case, 3rd person narration would be understood as a more distant mode which makes immersion more difficult, especially from the perspective of the protagonist. Alternatively, 1st person narration could be understood as a cue to understand the narrator as a person and listen to his or her story like an interlocutor in a conversation. Here, 3rd person would be the more open and flexible mode which leaves it open to the reader from which perspective she immerses (see also discussion in Hamburger, 1993).

At the same time, some accounts argue for a stronger role of narrative style and less influence of expectations toward factuality (van Krieken et al., 2015b). Therefore, as an alternative hypothesis, it is also possible that stories labeled as based on true events do not show a different behavioral pattern from fictional stories because both stories are in an equally engaging narrative form. This finding would support the idea that it is not the expectation toward a story about being based on factual or fictional events which is driving a certain reading behavior, but rather it is the narrative style which encourages readers to engage with a story in a certain way.

## Methods

All materials and data used in this study can be downloaded here: https://osf.io/uj6b4/

### Participants

All participants were naïve as to the purpose of the experiment. They gave informed consent in accordance with the declaration of Helsinki before the experiment started by accepting the use of their data and continuing to the instructions. The study fell under the approval of the local Ethics Committee of the Social Sciences faculty of the Radboud University (Ethics Approval Number ECG2013-1308-120).

#### Online sample

Participants were recruited via different online sources. Advertisements for the study were posted on several blogs, websites, Facebook, and Twitter accounts specifically targeting Dutch readers interested in language, reading, and research. Examples include regional libraries, the national foundation for reading (*Stichting lezen*), a Dutch language magazine (*Taalpost*), a literature collective (*Wintertuin*), and the Max Planck Institute for Psycholinguistics (MPI) websites including related pages (e.g., *www.hettaligebrein.nl*).

A total number of 2,100 people participated in our study. We restricted the analysis per task to datasets from participants who completed the task and were within a reasonable time per item (<three times the next slowest time). This means that the dropout rate increased per task in the experiment. For the first task (reading the story and fill in the immersion questionnaire) participants who took more than 5 min to read the instructions (*N* = 5), or did not fill in the immersion questionnaire at all (*N* = 186), or only partially (*N* = 60), as well as participants who took more than 1.5 min on average per item to respond to the questionnaire items were excluded from the analysis. In addition, four more subjects who took disproportionally long (>three times longer than the next slowest subject) to read the stories were excluded from the analysis. This adds up to a total exclusion of 257 participants for the immersion questionnaire task.

**Figure 1:**
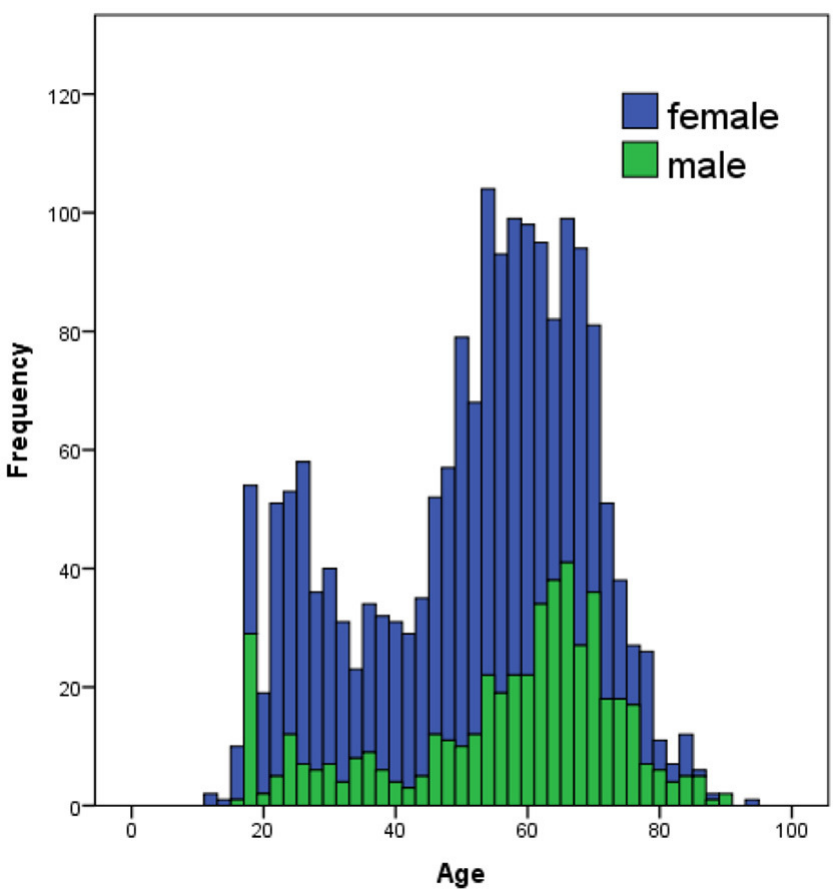
Distribution of age in the tested sample. Participants from 12 to 93 years participated in the study with a mean age of 51.33 years (s.d. = 17.08).

The data of 1,843 subjects (1,326 female, 497 male, 19 other) entered the analysis in its initial stage for the immersion questionnaire. Age varied considerably with a mean age of 51.33 years (s.d. = 17.08, range = 12–93 years, see Figure 1, Table 1). Most participants indicated that their highest educational level was university or technical college (specialized vocational or applied training; *N* = 1,485), but education level ranged from primary education (*primair onderwijs basisschool, N* = 4), high school (*voortgezet onderwijs, N* = 175), or community college (*middelbaar beroepsonderwijs MBO, N* = 145; other forms of education *N* = 27) to university level. Most participants (*N* = 1,651) were native speakers of Dutch. Non-native speakers (*N* = 87) were learning Dutch on average since 24.32 years (s.d. = 21.71, range = 1–82 years).

**Table 1:** Demographic information.

	***N***	**Minimum**	**Maximum**	**Mean**	**Std. Deviation**
Age	1,836	12	93	51.33	17.06
Education	1,809	1	4	3.72	0.64
Do you like fiction?	1,842	0	7	5.24	1.83
Do you like factual stories?	1,842	0	7	5.27	1.63
Dutch is native language	1,836	1 (yes)	2 (no)	1.10	0.30
If no, years learning Dutch	87	1	82	24.32	21.71

For the second task (the appreciation rating), further subjects were excluded from the analysis who did not participate in the appreciation rating at all (*N* = 28) or only partially (*N* = 6), as well as one participant who took more than three times as long as the next slowest participant to complete the task. This adds up to a total additional exclusion of 35 more participants for the appreciation rating (total *N* = 1,808). For the third task (reaction time picture task), additional datasets were excluded in which participants did not complete the task (*N* = 62), gave responses faster than 1 s on average per item (*N* = 1) or took on average more than two times the time the next slowest participant did (*N* = 3), leading to a total exclusion of 66 participants (*N* = 1,742).

#### Reference group

In order to have a reference group for identifying participants in the online sample who did not seriously participate in the experiment and to have a group with a more controlled setting for the reaction time task (picture task), we tested the experiment with a reference sample in the lab. We recruited 46 (30 female, 16 male) proficient speakers of Dutch without reading impairments via the Max Planck participant database in Nijmegen. Participants were students between 18 and 20 years old (mean age = 19.07). They were tested in a small, comfortable meeting room at the Max Planck Institute individually or in small groups (max. 3 participants) with the experimenter present in the room. Participants in the reference group were paid for compensation. The data obtained from these participants was analyzed separately.

### Stories

We used four short stories written by a young Dutch writer who studies creative writing (see Datasheet S1). Each story was used in 2 versions, one in 1st person perspective in which the protagonist is referred to with 1st person pronouns (“I”) and one in 3rd person perspective in which the protagonist is referred to with 3rd person pronouns (“he” or “she”; see Table 2 for more information). This way, we created the impression that the 1st person stories are told by the protagonist from a 1st person viewpoint whereas the 3rd person story seemed to be told about the protagonist by an invisible narrator who has access to the protagonist's thoughts. The stories were written to fit this manipulation.

**Table 2:** Number of words per story in both perspective conditions.

**Title**	**1st person perspective**	**3rd person perspective**
Emotioneel (*Emotional*)	336	338
Meesterwerk (*Master piece*)	571	573
Koffiemolen (*Coffee mill*)	884	880
Matroesjka (*Matryoshka*)	396	396

*The stories differed in the pronouns and the dependent verb forms, as well as some minor changes (1 change in Emotioneel and 3 changes in Koffiemolen) for readability (e.g., more colloquial expressions or writing conventions for 1st person stories)*.

All stories were narrated from the perspective of the protagonist with internal focalization. This means that the stories are told from the protagonist's subjective experience, see example below from the story “Matroesjka” (full transcript of the story in Datasheet S1).

*[…]There is a picture in a photo book in the filing cabinet upstairs, showing all cousins sitting around grandma in similar looking blue dresses. It was taken on her birthday. Every girl looks adorably into the camera as they should. Cheese*.

Except you and grandma. You're both looking naughty, as if grandma had just told you she has discovered where grandpa keeps his candy jar. Two pairs of straight noses with a small valley close to the tip, four bright blue eyes, grandmother's white hair sticking out from under her headscarf, your mouth slightly open, no idea yet what posing means, two pairs of apple cheeks, glowing like match heads. You don't remember it, you were only three when grandma died and I recently read that you don't remember anything before the age of four. But I just don't get it, when I look at that picture I just don't get it. Grandma so beautiful, you so beautiful - you were different, extraordinary. […]

### Questionnaires and tasks

#### Immersion questionnaire

To measure immersion into the stories, we used the SWAS (Kuijpers et al., 2014) with the subscales *attention, emotional engagement with the protagonist, mental imagery*, and *transportation into the story world*. The questionnaire contains 18 items with four to five items per subscale. We extended the subscale for mental imagery by adding two items, to account for differences depending on perspective taking: “*At times, I had the feeling of seeing right through the eyes of the protagonist*” and “*During reading, I saw the situations in my mind as if I was an eyewitness*” (see Datasheet S2 for details). Apart from the added items, the SWAS is a standardized measure of reading immersion with its subscales representing individual dimensions of absorption into narratives. Participants responded to the items on a 7-point scale ranging from “*I totally disagree*” (1) to “*I totally agree*” (7).

#### Appreciation rating

Story appreciation was measured directly after the immersion questionnaire. Participants saw 10 adjectives, which correspond to empirically established dimensions of appreciation (Knoop et al., 2016). For each adjective participants indicated how much they agreed that the adjective was applicable to the story [7-point scale ranging from “*I totally disagree*” (1) to “*I totally agree*” (7)]. The measure contained the following adjectives: *interesting, well-written, of high literary quality, easy to understand, accessible, thrilling, beautiful, fascinating, emotional, boring*, and *sad*.

#### Pictures for the event recognition task

For the picture task, we took photographs of situations similar to the ones described in the stories. For each story, we depicted two action events (16 pictures in total, see Datasheet S3). Each picture was taken from both the actor's perspective (1st person) and from an observer's perspective (3rd person, see Table 3). The photos were converted into stencil like pattern drawings with *Free Picture Stencil Maker* (Patrick Roberts Software, http://online.rapidresizer.com/photograph-to-pattern.php).

**Table 3:** Examples of picture stimuli taken from 1st and 3rd person perspective for the stories *Meesterwerk* and *Koffiemolen*.

**1^st^ person perspective**	**3^rd^ person perspective**
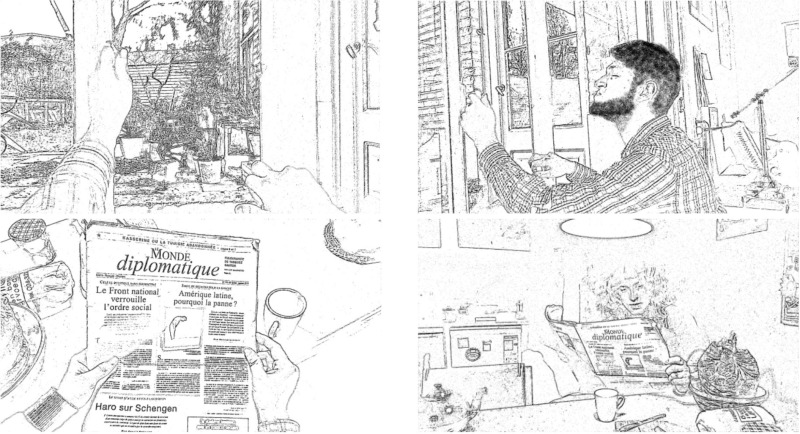	

#### Individual differences in engagement with fiction and non-fiction

We had six items addressing people's regular reading habits and other types of engaging with factual and fictional narratives like films or popular science books. Participants responded to the items on a 7-point scale ranging from “*not at all*”/”*never*” (=1) to “*very much*”/“*daily*” (=7). The 3 fiction items were: *Do you like reading fiction?*; *Do you engage with other types of fiction (e.g., movies or series, comic books, etc.)?*; *How often do you engage with fiction?*. The three factual items were: *Do you like reading non-fiction (stories based on true events)?*; *Do you engage with other types of non-fiction media [e.g., journal articles, science reports, (auto-) biographies, etc.]?*; *How often do you engage with non-fiction?*

### Procedure

The experiment started with a screen giving an overview of the goal of the experiment. Informed consent was acquired on this page when the subjects confirmed willing to participate by button click. On the subsequent screen, information about age, gender, education level, and proficiency in Dutch was acquired. Then, participants were randomly assigned to one of the two fictionality conditions, which prompted an instruction screen which was different for the two conditions:

**Table:** 

**Fact**	**Fiction**
You are going to read a story written by Martin Rombouts. He is a young Dutch columnist. He writes about his everyday life, always inspired by a real event.	You are going to read a story from Martin Rombouts. He is a young Dutch writer. He writes short fictional stories that are inspired by his imagination.

The introduction about the level of fictionality of the stories was the only difference between the two conditions. On the next screen, the story was presented. Each participant only read one of the stories in one version in either 1st or 3rd person perspective. There was no time limit and participants had to click a button at the end of the page to continue with the experiment. Total reading time of the story was measured from story screen onset to button click. After participants read the story, they responded to the items of the immersion questionnaire followed by the appreciation rating. Items were presented individually per screen in random order and subjects answered on a 7-point scale ranging from “I totally disagree” (=1) to “I totally agree” (=7). Selecting a value on the scale prompted the next screen.

After this, participants were presented with the picture task (Brunyé et al., 2009). Participants saw pictures and decided whether the scene displayed on the image happened in the story they just read or not. Each participant saw all 16 pictures, of which four were displaying two different situations from the story they just read. Every event was displayed once from the protagonist's perspective and once from the perspective of an eyewitness. The pictures from the stories that the participant had not read functioned as filler items. Participants were instructed to react as fast as possible. Reaction time was measured from the time of picture onset to button press.

Finally, participants responded to the six questions regarding their regular engagement with fiction and non-fiction. After these participants were debriefed and received some information about the writer and the research. Participants were given the option to sign up for receiving the results of the study. In total, the experiment took about 10–15 min.

### Apparatus

The data for this experiment was collected online by the use of a self-contained web application and a separate data submission/reporting web service, both of which were produced with FRINEX, (framework for interactive experiments) developed at Max Planck Institute for Psycholinguistics, Nijmegen. Participant responses were collected by the web application as time series data, which were sent to the server when a connection was available. The data submission/reporting web service was run in a Java Tomcat server using a Postgres database. Communication between the web application and the server was done over a JSON/REST interface. If the connection to the server failed during this communication process, then the web application stored the data and retried later in the experiment. This retry/store process could continue if required until specific points in the experiment such as the registration screen, where a successful submission was mandated before proceeding. This combination allowed users to do the experiment on desktop computers or mobile devices and in environments with periodic internet access such as when commuting.

The application flow was restricted to linear navigation with each screen being visible once in its sequence. Neither refreshing the browser nor using the browser back button would alter this linear application flow. The participant could exit the experiment at any stage; with the data from their participation having already been stored on the server provided internet access was available.

### Data analysis

The data were averaged for each scale of the immersion questionnaire. For each other measure (e.g., appreciation questionnaire, reading time, individual differences), we used the single value entered for each measure by the participants or the time stamp difference between button presses.

The data were analyzed with R implemented in the RStudio GUI, using the nlme library for testing linear mixed models (Pinheiro et al., 2014). The data of the online sample and the reference group, which was tested in the lab were analyzed separately. Each of the measures was analyzed in a separate model with fictionality (fact or fiction) and perspective (1st or 3rd person) as predictors which were allowed to interact, and story as random effect with random intercept (Baayen et al., 2008). In addition, individual differences in preference for perspective taking, gender, age, education level, whether Dutch was native language or not, and the two mean scores for general exposure to fictional and factual stories were included as factors in the model. For the 2 models testing reaction time in the picture recognition task we also included whether the response was correct or not. Age, education level, and whether they were native speakers of Dutch were not included in the model for the reference group because of the homogeneity of the sample. P-values for specific effects were obtained by a model comparison procedure using the asymptotic chi square distribution. Statistical details about all models and results can be found in the supplementary materials (Datasheets S4, S5).

## Results

### Summary of results

Because of the number of measures, we first report a summary of findings, followed by statistical details per measure. We want to point out that due to our large sample size, effects with very small effect sizes can become statistically significant. Here, we only report main effects and effects with |β| > 0.004, which leads to a fair representation of the results without hindering readability from reporting very small effects. For ease of reading, the results of the reference group are only reported for the picture task as they did not differ drastically from the online group. Details about the statistical models and the results of both groups can be found in the supplementary material (Datasheet S4).

Whether the stories were presented as fictional or factual had no influence on how long participants spent on reading the stories, or on any of the immersion subscales. There are also no statistically significant effects for fictionality for any of the appreciation measures, but there was a trend suggesting that fiction stories were rated as less easy to understand than factual stories (β = −0.58, s.e. = 0.31, *t* = −1.86, *p* = 0.06). In sum, whether stories were presented as fictional or as factual did not influence reading experience as we measured it.

For perspective (1st or 3rd person), the second factor of interest, several statistically significant effects were observed. Stories in 1st person showed higher scores for emotional engagement with the protagonist (β = −0.13, s.e. = 0.06, *t* = −2.09, *p* < 0.05) and people were more likely to engage in 1st person perspective taking in 1st person stories (β = −0.24, s.e. = 0.11, *t* = −2.16, *p* < 0.05). There was no effect of perspective on reading time, nor on the immersion subscales attention, transportation, and mental imagery. Stories in 3rd person were rated as sadder (β = 0.31, s.e. = 0.10, *t* = 2.97, *p* < 0.005), and there was a statistically significant interaction between perspective and fictionality on this item (β = −0.31, s.e. = 0.15, *t* = −2.13, *p* < 0.05). In addition, there was a trend suggesting that 3rd person stories were rated as less fascinating (β = −0.15, s.e. = 0.09, *t* = −1.79, *p* = 0.07). Otherwise, none of the appreciation measures were affected by the perspective of the story and there were no interaction effects.

Regarding individual differences, liking fiction was generally associated with faster reading (β = −3.31, s.e. = 0.71, *t* = −4.64, *p* < 0.0001), higher probability for 1st person perspective taking (β = 0.03, s.e. = 0.01, *t* = 2.03, *p* < 0.05), and lower ratings on the items “well-written” and “literary” (−0.03 < β < −0.02, s.e. = 0.01, −2.44, *t* < −2.21, *p* < 0.05) in all conditions. High scores for both 1st person perspective preference and 3rd person perspective preference were associated with higher scores on all scales of the immersion questionnaire (0.24 < β < 0.47, 0.01 < s.e. ≤ 1.67, 14.89 < *t* < 28.91, *p* < 0.0001) and on almost all items of the appreciation rating (0.24 < β < 0.47, 0.01 < s.e. ≤ 1.67, 14.89 < *t* < 28.91, *p* < 0.0001). In addition, 1st and 3rd person preference were associated with lower scores on the rating whether the stories were perceived as sad.

There was a small age effect throughout most measures indicating that older readers score slightly lower on the immersion scales transportation, emotional engagement, and mental imagery (−0.42 < β < −0.01, 0.00 < s.e ≤ 0.00, −4.15 < *t* < −2.94, *p* < 0.005). An additional, and exploratory correlation analysis showed that age was negatively correlated with liking fictional texts (*r* = −0.16, *p* < 0.0001) and positively with liking factual texts (*r* = 0.07, *p* < 0.05) as tested by a partial correlation analysis controlling for gender and education.

In the picture task, we observe no effect for fictionality or perspective. There were no differences in the reaction times to pictures associated with condition, perspective, or perspective taking preference.

For the distribution of effects between stories, see Datasheet 6, the full data set is available for further analyses and replication on Datasheet 7.

### Full description of results

#### Perspective taking

**Figure 2:**
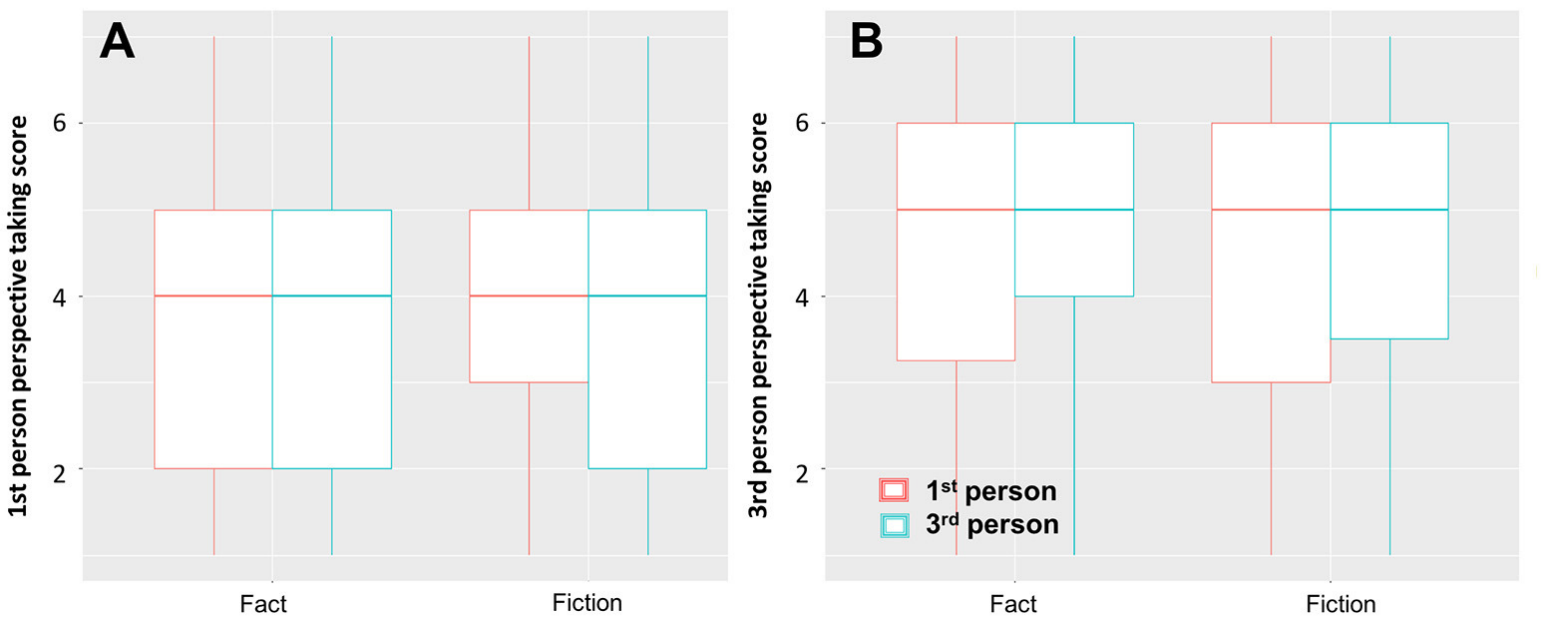
Differences in perspective taking; **(A)** 1st person perspective taking, **(B)** 3rd person perspective taking. There was no difference in perspective taking depending on whether the stories were presented as factual or fictional. Stories in 1st person perspective were rated significantly higher for 1st person perspective taking than stories in 3rd person perspective.

For the **1st person perspective taking** questionnaire item we observe a main effect of perspective (β = −0.24, s.e. = 0.11, *t* = −2.16, *p* < 0.05, see Figure 2) meaning that readers are less likely to engage in 1st person perspective taking when reading a story in 3rd person perspective. There is no main effect of fictionality (β = 0.39, s.e. = 0.43, *t* = 0.91, *p* = 0.36) and no interaction effect with perspective (β = 0.15, s.e. = 0.15, *t* = 0.96, *p* = 0.34). People who report to like fiction are more likely to engage in 1st person perspective taking (β = 0.03, s.e. = 0.01, *t* = 2.03, *p* < 0.05).

For the **3rd person perspective taking** questionnaire item we observe no main effect of perspective (β = −0.06, s.e. = 0.11, *t* = −0.58, *p* = 0.56, see Figure 2) or fictionality (β = 0.01, s.e. = 0.41, *t* = 0.03, *p* = 0.98), and no interaction effect (β = 0.12, s.e. = 0.15, *t* = 0.81, *p* = 0.42). Moreover, we found that older (β = −0.01, s.e. = 0.00, −4.03 < *t* < −3.03, *p* < 0.005) and male readers (−0.31 < β < −0.26, 0.08 < s.e. < 0.09, −3.77 < *t* < −3.00, *p* < 0.005) are less likely to engage in perspective taking regardless of perspective.

#### Reading time

**Figure 3:**
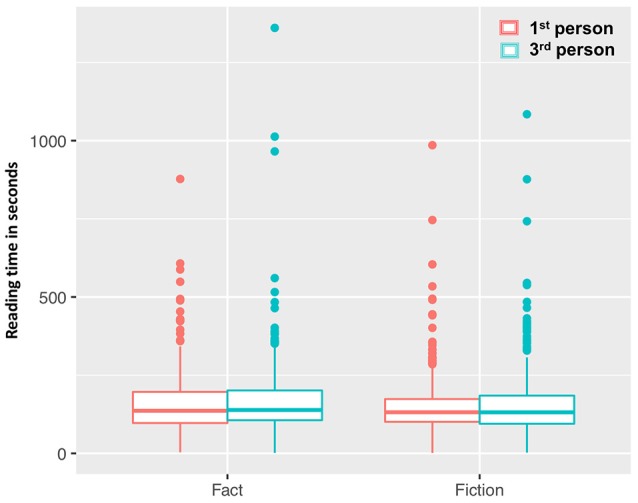
Time in seconds participants took to read the story. There was no difference between reading times in the fictional or factual condition, as well as no difference in reading time dependent on perspective.

For the measure of how long participants took to read the story, there were no effects for fictionality (β = 2.53, s.e. = 21.39, *t* = 0.12, *p* = 0.91, see Figure 3) or perspective (β = 9.17, s.e. = 5.85, *t* = 1.57, *p* = 0. 12), and no interaction effect (β = −0.14, s.e. = 8.25, *t* = −0.02, *p* = 0.99). When looking at individual differences, we observe that readers for whom Dutch is the native language read faster (β = −28.60, s.e. = 6.81, *t* = −4.20, *p* < 0.0001). Liking fiction was also associated with shorter reading times (β = −3.31, s.e. = 0.71, *t* = −4.64, *p* < 0.0001).

#### Immersion

**Figure 4:**
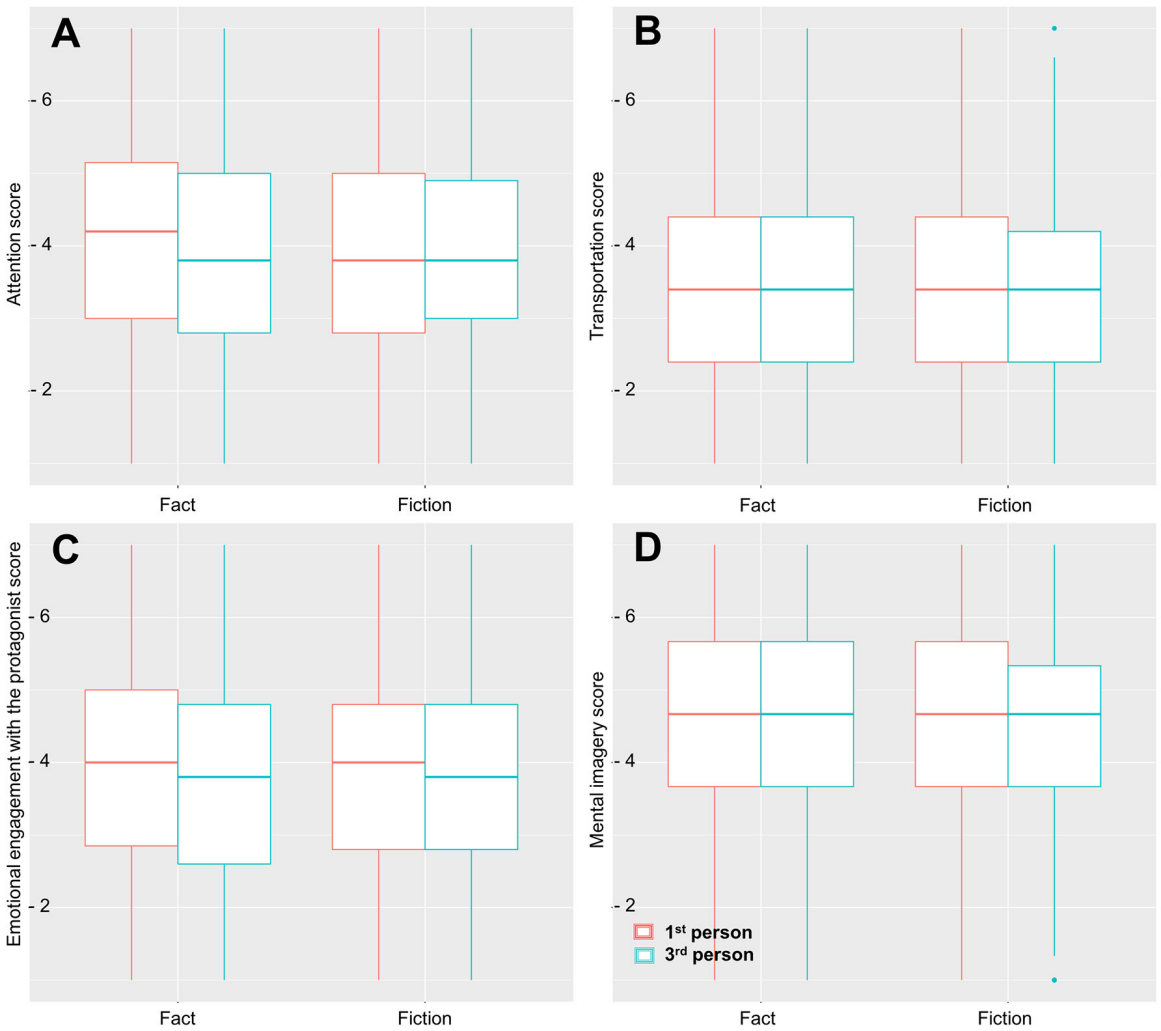
Effect on the immersion subscales; **(A)** Attention, **(B)** Transportation, **(C)** Mental imagery, **(D)** Emotional engagement. There was no difference in immersion depending on whether the stories were presented as factual or fictional. Stories in 1st person perspective had significantly higher scores for attention and emotional engagement with the protagonist compared to stories in 3rd person perspective, but not for transportation and mental imagery during reading.

For the **attention** ratings, we observe no main effect of perspective (β = −0.12, s.e. = 0.07, *t* = −1.69, *p* = 0.09), or fictionality (β = 0.04, s.e. = 0.26, *t* = 0.14, *p* = 0.89), and no interaction (β = 0.13, s.e. = 0.10, *t* = 1.32, *p* = 0.19; see Figure 4). Both 1st (β = 0.36, s.e. = 0.02, *t* = 19.91, *p* < 0.0001) and 3rd (β = 0.29, s.e. = 0.02, *t* = 15.70, *p* < 0.0001) person perspective taking were associated with higher scores on the attention scale.

For **transportation**, there were no main effects of fictionality (β = 0.09, s.e. = 0.23, *t* = 0.41, *p* = 0.69) and perspective (β = −0.01, s.e. = 0.06, *t* = −0.24, p = 0.81), and no interaction (β = 0.03, s.e. = 0.09, *t* = 0.34, *p* = 0.73; see Figure 4). Both 1st (β = 0.42, s.e. = 0.02, *t* = 26.87, *p* < 0.0001) and 3rd (β = 0.27, s.e. = 0.02, *t* = 16.52, *p* < 0.0001) person perspective taking were associated with higher scores for transportation.

For **emotional engagement with the protagonist** we observe a main effect of perspective (β = −0.13, s.e. = 0.01, *t* = −2.09, *p* < 0.05) showing that readers are less engaged when reading a story in 3rd person perspective (β = 0.01, s.e. = 0.00, *t* = 0.92, *p* = 0.36; see Figure 4). There was no effect of fictionality (β = 0.00, s.e. = 0.0.23, *t* = 0.13, *p* = 0.89) and no interaction of fictionality and perspective. Both 1st (β = 0.46, s.e. = 0.00, *t* = 28.91, *p* < 0.0001) and 3rd person perspective taking (β = 0.25, s.e. = 0.00, *t* = 14.89, *p* < 0.0001) were associated with higher scores for emotional engagement with the protagonist.

For **mental imagery**, we observe no main effect of perspective (β = 0.02, s.e. = 0.06, *t* = 0.37, *p* = 0.71) or fictionality (β = 0.11, s.e. = 0.23, *t* = 0.46, *p* = 0.64), and no interaction (β = −0.07, s.e. = 0.09, *t* = −0.75, *p* = 0.45; see Figure 4). Both 1st (β = 0.30, s.e. = 0.02, *t* = 19.26, *p* < 0.0001) and 3rd (β = 0.38, s.e. = 0.02, *t* = 23.95, *p* < 0.0001) person perspective taking were associated with higher scores for mental imagery.

#### Appreciation

For the rating how **interesting** the story was, we observe no main effect of perspective (β = −0.13, s.e. = 0.09, *t* = −1.47, *p* = 0.14) or fictionality (β = −0.23, s.e. = 0.27, *t* = −0.83, *p* = 0.41), and no interaction (β = 0.02, s.e. = 0.13, *t* = 0.16, *p* = 0.87). Both 1st (β = 0.32, s.e. = 0.02, *t* = 14.17, *p* < 0.0001) and 3rd (β = 0.28, s.e. = 0.02, *t* = 11.63, *p* < 0.0001) person perspective taking were associated with higher appreciation scores for interesting.

The rating of how **well-written** the story was rated revealed no effect for perspective (β = −0.16, s.e. = 0.10, *t* = −1.62, *p* = 0.10) or for fictionality (β = −0.20, s.e. = 0.29, *t* = −0.68, *p* = 0.50), and no interaction (β = −0.10, s.e. = 0.14, *t* = −0.71, *p* = 0.48). Readers who scored high on liking fiction rated the stories as less well-written (β = −0.02, s.e. = 0.01, *t* = −2.21, *p* < 0.05). Both 1st (β = 0.30, s.e. = 0.02, *t* = 12.58, *p* < 0.0001) and 3rd person perspective taking (β = 0.30, s.e. = 0.03, *t* = 12.01, *p* < 0.0001) were associated with higher appreciation scores for interesting.

The rating of how **literary** the stories were showed no main effect for perspective (β = 0.03, s.e. = 0.09, *t* = 0.39, *p* = 0.70) and fictionality (β = 0.03, s.e. = 0.26, *t* = 0.12, *p* = 0.90), and no interaction (β = −0.22, s.e. = 0.13, *t* = −1.73, *p* = 0.08). Both 1st (β = 0.26, s.e. = 0.02, *t* = 11.70, *p* < 0.0001) and 3rd (β = 0.26, s.e. = 0.02, *t* = 11.28, *p* < 0.0001) person perspective taking were associated with higher scores for the rating if the story was considered literary. Older readers rated the stories as less literary (β = −0.01, s.e. = 0.00, *t* = −2.89, *p* < 0.0001), and readers who score high on liking fictional stories rated them as less literary (β = −0.03, s.e. = 0.01, *t* = −2.44, *p* < 0.05). There was a significant interaction of liking fictional texts and whether the participant was in the factual or fictional condition (β = 0.03, s.e. = 0.01, *t* = 2.38, *p* < 0.05).

The rating of how **easy to understand** the stories were showed no main effect of perspective (β = −0.14, s.e. = 0.10, *t* = −1.36, *p* = 0.09). There was a trend for an effect of fictionality (β = −0.58, s.e. = 0.31, *t* = −1.86, *p* = 0.06) showing that readers in the fiction condition rated the stories as less easy to understand, but no interaction with perspective (β = −0.19, s.e. = 0.15, *t* = −1.27, *p* = 0.20). Both 1st (β = 0.18, s.e. = 0.03, *t* = 6.87, *p* < 0.0001) and 3rd person perspective taking (β = 0.14, s.e. = 0.03, *t* = 5.13, *p* < 0.0001) were associated with higher scores for easy to understand. Looking at individual differences reveals that male readers rated the stories as less easy to understand (β = −0.20, s.e. = 0.08, *t* = −2.37, *p* < 0.005), whereas older readers were more likely to rate the story as easy to understand (β = 0.01, s.e. = 0.00, *t* = 4.81, *p* < 0.0001). There was a main effect of educational level (β = −0.17, s.e. = 0.06, *t* = −2.80, *p* < 0.01) showing that readers with higher education were less likely to rate the stories as easy to understand.

For the rating on how **accessible** the stories were, we find no main effect for perspective (β = −0.10, s.e. = 0.10, *t* = −0.99, *p* = 0.32) and fictionality (β = −0.28, s.e. = 0.29, *t* = −0.95, *p* = 0.34), and no interaction (β = −0.09, s.e. = 0.14, *t* = −0.65, *p* = 0.52). Both 1st (β = 0.22, s.e. = 0.02, *t* = 9.19, *p* < 0.0001) and 3rd person perspective taking (β = 0.22, s.e. = 0.03, *t* = 8.49, *p* < 0.0001) were associated with higher scores for accessible. Older readers rated the stories as more accessible (β = 0.01, s.e. = 0.00, *t* = 2.37, *p* < 0.05).

For the rating on how **thrilling** the stories were, we find no main effect for perspective (β = −0.08, s.e. = 0.09, *t* = −0.92, *p* = 0.36) and fictionality (β = 0.16, s.e. = 0.28, *t* = 0.59, *p* = 0.56), and no interaction (β = −0.07, s.e. = 0.13, *t* = −0.54, *p* = 0.59). Both 1st (β = 0.27, s.e. = 0.02, *t* = 11.87, *p* < 0.0001) and 3rd person perspective taking (β = 0.22, s.e. = 0.02, *t* = 9.53, *p* < 0.0001) were associated with higher scores for thrilling. Native speakers rated the stories as less thrilling (β = −0.24, s.e. = 0.11, *t* = −2.30, *p* < 0.05) while older readers rated the stories as more thrilling (β = 0.01, s.e. = 0.00, *t* = 4.57, *p* < 0.0001).

For the rating on how **beautiful** the stories were, we find no main effect for perspective (β = −0.07, s.e. = 0.09, *t* = −0.87, *p* = 0.38) and fictionality (β = −0.40, s.e. = 0.27, *t* = −1.51, *p* = 0.13), and no interaction (β = 0.02, s.e. = 0.13, *t* = 0.17, *p* = 0.86). Both 1st (β = 0.32, s.e. = 0.02, *t* = 14.21, *p* < 0.0001) and 3rd person perspective taking (β = 0.27, s.e. = 0.02, *t* = 11.35, *p* < 0.0001) were associated with higher scores for beautiful. Older readers rated the stories as less beautiful (β = −0.01, s.e. = 0.00, *t* = −4.01, *p* < 0.0001).

For the rating on how **fascinating** the stories were, we observe a trend that 3rd person stories are rated as less fascinating (β = −0.15, s.e. = 0.09, *t* = −1.79, *p* = 0.07). There was no effect for fictionality (β = −0.01, s.e. = 0.26, *t* = −0.02, *p* = 0.98) and no interaction with perspective (β = 0.01, s.e. = 0.12, *t* = 0.12, *p* = 0.90). Again, both 1st (β = 0.34, s.e. = 0.02, *t* = 15.60, *p* < 0.0001) and 3rd person perspective taking (β = 0.31, s.e. = 0.02, *t* = 13.87, *p* < 0.0001) were associated with higher scores for fascinating.

For the rating on how **emotional** the stories were, we find no main effect for perspective (β = −0.06, s.e. = 0.09, *t* = −0.68, *p* = 0.49) and fictionality (β = −0.46, s.e. = 0.28, *t* = −1.68, *p* = 0.09), and no interaction (β = 0.01, s.e. = 0.13, *t* = 0.05, *p* = 0.96). Both 1st (β = 0.31, s.e. = 0.02, *t* = 13.40, *p* < 0.0001) and 3rd person perspective taking (β = 0.24, s.e. = 0.02, *t* = 9.81, *p* < 0.0001) were associated with higher scores for emotional. Male readers rated the stories as more emotional (β = 0.21, s.e. = 0.07, *t* = 2.77, *p* < 0.01). Moreover, there was a significant interaction of fictionality and whether readers like fiction (β = 0.04, s.e. = 0.01, *t* = 2.43, *p* < 0.05).

The rating of how **sad** the stories were revealed that 3^rd^ person stories were rated as sadder (β = 0.31, s.e. = 0.10, *t* = 2.97, *p* < 0.005). There was no main effect for fictionality (β = −0.02, s.e. = 0.31, *t* = −0.07, *p* = 0.94). However, there was an interaction effect of fictionality and perspective (β = −0.31, s.e. = 0.15, *t* = −2.13, *p* < 0.05), meaning that 3rd person stories were rated as sadder when thought to be based on true events as compared to fictional events. In contrast to all other appreciation measures 1st (β = −0.24, s.e. = 0.03, *t* = −9.26, *p* < 0.0001) and 3rd person perspective taking (β = −0.11, s.e. = 0.02, *t* = −4.07, *p* < 0.0001) were associated with lower scores on the rating whether the stories were perceived as sad indicating that readers who engage in perspective taking rated the stories as less sad.

#### Picture task

**Figure 5:**
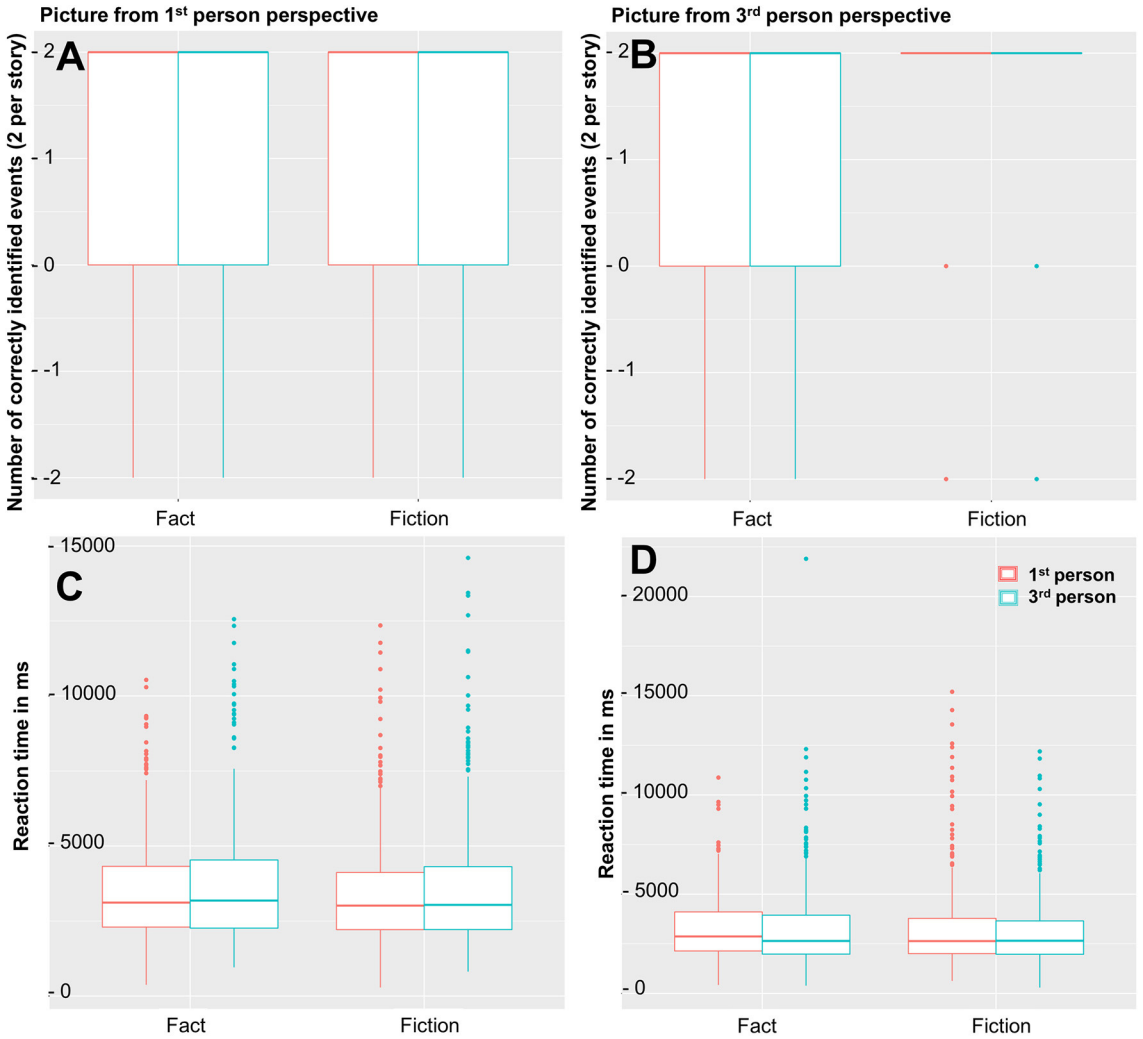
Accuracy and reaction times in responses to events pictured from 1st person perspective (**A** = accuracy, **C** = reaction time) and 3rd person perspective (**B** = accuracy, **D** = reaction time). There were no significant effects of fictionality or perspective.

The **accuracy** rates in the picture task on **pictures depicting events from the 1st person perspective** showed no effect of perspective (β = −0.02, s.e. = 0.09, t = −0.22, *p* = 0.83) or fictionality (β = −0.21, s.e. = 0.39, *t* = −0.53, *p* = 0.60), and no interaction (β = −0.07, s.e. = 0.13, *t* = −0.52, *p* = 0.60; see Figure 5). Readers who engaged in 1st person perspective taking responded more accurate (β = 0.07, s.e. = 0.02, *t* = 3.14, *p* < 0.005), but there was no advantage for readers who engage in 3rd person perspective taking (β = −0.01, s.e. = 0.02, *t* = −0.34, *p* = 0.74). In the reference group, we also did not observe main effects for perspective (β = −0.49, s.e. = 0.40, *t* = −1.25, *p* = 0.23) or fictionality (β = −3.07, s.e. = 2.22, *t* = −1.38, *p* = 0.21), no interaction, and also no effects for perspective taking preference (|β| < 0.10, s.e. < 0.13, |t| < 0.89, 0.40 < *p* < 0.46).

The **accuracy** rates in the picture task on **pictures depicting events from the 3rd person perspective** also showed no effect of perspective (β = −0.00, s.e. = 0.01, *t* = −0.22, *p* = 0.83) or fictionality (β = 0.02, s.e. = 0.31, *t* = 0.59, *p* = 0.55), and no interaction (β = −0.01, s.e. = 0.01, *t* = −0.74, *p* = 0.46; see Figure 5). Both 1st (β = 0.05, s.e. = 0.02, *t* = 2.79, *p* < 0.01) and 3rd person perspective taking (β = 0.05, s.e. = 0.02, *t* = 2.56, *p* < 0.05) were associated with better accuracy in responding to pictures in from 3rd person perspective. Native speakers responded more accurately than non-native speakers (β = 0.21, s.e. = 0.09, *t* = 2.41, *p* < 0.05). Likewise, in the reference group, there were no main effects of perspective (β = −0.37, s.e. = 0.29, *t* = −1.25, *p* = 0.22) or fictionality (β = −2.34, s.e. = 1.71, *t* = −1.36, *p* = 0.20), and no interaction. However, there were main effects for perspective taking preference: whereas 1st person perspective taking was associated with lower accuracy (β = −0.23, s.e. = 0.09, *t* = −2.48, *p* < 0.05), 3rd person perspective taking was associated with higher accuracy (β = 0.26, s.e. = 0.08, *t* = 3.22, *p* < 0.005).

The **reaction times** toward **pictures depicting events from the 1st person perspective** showed no effect of perspective (β = 131.22, s.e. = 126.90, *t* = 1.03, *p* = 0.30) or fictionality (β = −737.94, s.e. = 559.07, *t* = −1.32, *p* = 0.19), and no interaction (β = 20.52, s.e. = 179.41, *t* = 0.11, *p* = 0.90; see Figure 5). Moreover, there are no effects for perspective taking (1st person: β = 27.75, s.e. = 31.77, *t* = 0.87, *p* = 0.38; 3rd person: β = 56.83, s.e. = 33.27, *t* = 1.71, *p* = 0.09). There was a main effect of whether the response was correct or not (β = −139.90, s.e. = 34.80, *t* = −4.02, *p* < 0.0001), and a main effect of age showing that older readers responded slower (β = 34.53, s.e. = 2.78, *t* = 12.42, *p* < 0.0001). In the reference group, we also did not observe main effects for perspective (β = 785.67, s.e. = 503.25, *t* = 1.56, *p* = 0.13) or fictionality (β = 2.96, s.e. = 2799.60, *t* = 0.001, *p* = 1.00), and no interaction, as well as no effects for perspective taking (1st person: β = 42.56, s.e. = 156.71, *t* = 0.27, *p* = 0.79; 3rd person: β = 146.65, s.e. = 136.31, *t* = 1.08, *p* = 0.32).

The **reaction times** toward **pictures depicting events from the 3rd person perspective** showed no effect of perspective (β = 133.47, s.e. = 116.92, *t* = 1.14, *p* = 0.25) or fictionality (β = −441.81, s.e. = 514.98, *t* = −0.86, *p* = 0.39), no interaction (β = −196.42, s.e. = 165.50, *t* = −1.19, *p* = 0.24; see Figure 5), and no effects for perspective taking (1st person: β = −13.13, s.e. = 29.29, *t* = −0.45, *p* = 0.65; 3rd person: β = 45.26, s.e. = 30.74, *t* = 1.747, *p* = 0.14). Male readers responded faster (β = −229.20, s.e. = 94.89, *t* = −2.42, *p* < 0.05) whereas older readers responded slower (β = 30.38, s.e. = 2.56, *t* = 11.88, *p* < 0.0001). Higher education level (β = −140.48, s.e. = 67.39, *t* = −2.09, *p* < 0.05) and liking fiction (β = −170.47, s.e. = 43.02, *t* = −3.96, *p* < 0.0001) were associated with faster reaction times. Liking factual texts on the other hand was associated with slower reaction times (β = 117.51, s.e. = 55.28, *t* = 2.13, *p* < 0.05). In the reference group, there was also no main effect of fictionality (β = 2387.39, s.e. = 1288.68, *t* = 1.85, *p* = 0.10) or perspective (β = 19.91, s.e. = 228.69, *t* = 0.09, *p* = 0.93), and no interaction. In addition, there were also no effects for perspective taking (1st person: β = 124.78, s.e. = 75.84, *t* = 1.65, *p* = 0.12; 3rd person: β = 81.80, s.e. = 67.17, *t* = 1.22, *p* = 0.29). Liking factual text showed a trend to be associated with slower reaction times (β = 283.98, s.e. = 146.40, *t* = 1.94, *p* = 0.07).

## Discussion

In the present study, we tested the influence of perspective referring to protagonists of short stories labeled as fictional or as based on true events. We measured immersion and appreciation as well as memory for events depicted in the stories with an online study reaching a broad sample of readers from all ages.

In line with previous research we found that 1st person stories facilitate 1st person perspective taking. In addition, we found that 1st person stories can lead to higher emotional engagement with the protagonist compared to 3rd person stories. However, we did not replicate earlier findings (Hartung et al., 2016) that 1st person stories generally increase immersion and are liked better by readers on any of our appreciation measures. The only appreciation measure in which we find a difference between 1st and 3rd person stories is on the item “sad.” Here, 3rd person stories were rated as sadder than 1st person stories. Moreover, we found that people who like reading fiction generally read faster and are more likely to engage in 1st person perspective taking. Despite not finding effects for the perspective in which the story is narrated, we find evidence that perspective taking influences immersion and appreciation of stories. Readers who engage in perspective taking, regardless of whether they select 1st or 3rd person perspective, report higher immersion during reading and like the stories better.

We did not replicate previously reported evidence that personal pronouns (perspective) affect perspective taking of the memory representation for events (Brunyé et al., 2009). Instead, we found evidence that people who engage in 1st person perspective taking during reading respond more accurately to pictures from 1st and 3rd person perspective, whereas readers who engage in 3rd person perspective taking only have an advantage in responding to pictures from 3rd person perspective. This suggests that engaging in a story from a 1st person perspective allows readers to construct a more flexible mental representation of the events in the story compared to readers who immerse from a spectator's perspective. We find no reaction time advantages in the picture recognition task associated with perspective taking.

Despite evidence that readers are more likely to engage in 1st person perspective imagery when reading 1st person stories, we cannot replicate the perspective effects for the picture recognition task (Brunyé et al., 2009; Ditman et al., 2010). This could be attributed to the less controlled settings in our online study as compared to typical behavioral experiments in the lab. Yet, we also do not observe any trend for an effect in the reference group. These effects seem to be difficult to replicate (see this replication attempt of the same lab in response to a failed replication by another group: http://goo.gl/KR2Z4S). There is evidence for large individual variation in perspective taking (Vukovic and Williams, 2015; Brunyé et al., 2016) and it is likely that individual differences have a stronger influence on memory of events than the perspective manipulation.

The finding that perspective can influence some aspects of reading is in line with previous research (Hartung et al., 2016). However, in contrast to the findings reported by Hartung et al. (2016) we found that 1st person stories compared to 3rd person stories mainly increase the probability that the reader engages in 1st person perspective taking and shares emotions with the protagonist. Engaging in perspective taking during reading in turn seems to increase immersion and appreciation across all measures, so the pronoun effect reported by earlier research is likely to be an indirect effect of perspective taking and might also vary for different stories. Future research is needed to scrutinize this finding in more detail.

There were some notable individual differences dependent on whether people have a general preference for engaging with fictional or factual stories. We found that avid readers of fiction are also faster readers which is in line with the notion that reading goals associated with fiction are linked to reduced scrutiny and attention to detail (Green et al., 2006) but could also be attributed to a training effect. Moreover, avid readers are more likely to engage in 1st person perspective taking which could be related to the hypothesis that fiction reading is linked to empathy and perspective taking (e.g., Keen, 2007; Kidd and Castano, 2013). In addition, we find age-related differences in multipole measures indicating that older readers generally score lower on most of our measures. As the effect sizes are negligibly small, we refrain from interpreting them because it is likely that these effects are linked to the type of material we chose or older readers being more critical rather than being an effect of psychological interest.

We found throughout all our measures no evidence that knowing that a story is based on true or fictional events affects reading behavior, experiential aspects of reading, or memory for events in the stories. The results show that the belief a reader has about whether a story is based on a true event or not has no effect on the experiential aspects of reading such as immersion and appreciation of stories. This is in line with accounts that argue that an engaging narrative style is more important than readers' expectations about the fictionality of the information (van Krieken et al., 2015a).

The fact that we do not observe any difference between stories labeled as based on true or fictional events seems to be in contrast with previous experimental research on the effects of factuality on reading behavior which showed that factual and fictional stories are read differently (Zwaan, 1994; Altmann et al., 2014). Yet, we think that our findings are complementary rather than in contrast with previous findings. Typically, studies used a “newspaper” vs. “literature” labeling to manipulate readers expectations toward factuality (e.g., Zwaan, 1994). This manipulation does not only address factuality of the information, but likely is confounded with different genre and reading situation dependent contexts and reading goals. In our study, we used literary short stories in both conditions labeled as being factual or made up. The manipulation we used is subtler in a sense, because whether the story was believed to be based on true events and characters, or entirely fictional was the only factor being manipulated. This is both an advantage as well as the main limitation of our study as it is easily argued that our manipulation was too subtle and did not work. Yet, we think that this null-finding is interesting and raises an important conceptual issue with the current research. While differences in reading behavior have previously been attributed to readers' expectations regarding factuality of the events, it is very possible that the effects are based on genre specific reading goals. Based on our finding we suggest that the reading goals which are associated with certain reading contexts are more important drivers for reading behavior than whether the story is believed to be fictional or not.

The previously reported effects might therefore be better attributed to systematic effects of reading situation rather than the factuality of the content. While expository texts like newspaper or textbooks are all about extracting relevant information in appropriate detail, narratives whether they are true or not are often about people and social knowledge. This interpretation is fully in line with the theory that readers activate the appropriate reading goals for the current situation and systematically select criteria and strategies for comprehension (Zwaan, 1994; van den Broek et al., 2001, 2005). Reading narratives clearly activates different reading goals than non-narrative texts, but true and fictional narratives don't necessarily differ in the reading goals that they trigger. Our results and interpretation however are limited to the materials we used in this study. It is entirely possible that the reading experience of stories with different content than the stories that we used here (e.g., stories containing more emotional events) *is* influenced by fictionality. A related possibility is that in some genres fictionality is important, but in others it is not. Future research is needed to better qualify the interaction of factuality with different types of texts and reading goals. The present study provides experimental evidence that prior knowledge about a story being fictional or based on true events does not influence reading behavior. Instead, it seems that reading goals associated with certain situations and types of text are stronger predictors of reading behavior. This finding could be of relevance for accounts arguing for an educational role of fiction reading in social learning (Oatley, 1999b; Mar and Oatley, 2008). We showed that that value of fiction narrative may have more to do with the narrative character of the materials (the fact that they are *narratives*) than with whether they are fiction or not.

## Author contributions

The study was conceived and designed by FH and RW. PW wrote the software for the experiment and contributed to the experiment design and data collection. The data were analyzed by FH. FH, PH, and RW were involved in interpretation of the results. FH and PW wrote the initial draft of the manuscript, RW and PH contributed critically to revising it until its current form. All authors approve the current version and agree to be accountable of all aspects of the work.

### Conflict of interest statement

The authors declare that the research was conducted in the absence of any commercial or financial relationships that could be construed as a potential conflict of interest.
