# Supplementary material for: When Fiction Is Just as Real as Fact: No Differences in Reading Behavior between Stories Believed to be Based on True or Fictional Events
Source: Front Psychol. 2017 Sep 20;8:1618. doi: 10.3389/fpsyg.2017.01618 (PMC5613255; doi:10.3389/fpsyg.2017.01618)
Supplement: Supplementary file 1 [file DataSheet1.DOCX]

# S1: Stories

## Story 1: Matroesjka by Martin Rombouts

### Matryoshka – I-form (English Translation, 1st person)

‘She really looks like grandma,’ they said at Easter, birthdays and Pentecost, when they saw you playing in the garden. You wore the old skirts and dresses from your cousins, who looked like grandma as well, but not as much as you did. There is a picture in a photo book in the filing cabinet upstairs, showing all cousins sitting around grandma in similar looking blue dresses. It was taken on her birthday. Every girl looks adorably into the camera as they should. *Cheese*.

Except you and grandma. You’re both looking naughty, as if grandma had just told you she has discovered where grandpa keeps his candy jar. Two pairs of straight noses with a small valley close to the tip, four bright blue eyes, grandmother's white hair sticking out from under her headscarf, your mouth slightly open, no idea yet what posing means, two pairs of apple cheeks, glowing like match heads. You don't remember it, you were only three when grandma died and I recently read that you don't remember anything before the age of four. But I just don't get it, when I look at that picture I just don't get it. Grandma so beautiful, you so beautiful - you were different, extraordinary.

Yet I'm proud of you, sis. You decided to do it and did it; you lost weight like a matryoshka. But it seems like every bit of fat that disappears takes some of what defines you with it. You change. When mom and dad were away for a weekend lately, you asked for a salad at the snack bar. In the evening, you watched a movie in your room with a friend. I could hear you and him laughing through the wall. Your voice is higher nowadays.

During the most recent Easter egg hunt in grandma's - now aunt Liesbeth's - garden, you gave all the eggs you found to me. Even your favorites, the white ones containing praline.
‘You can handle it,’ you said, when you put them in my hands.
Before I knew it, it had slipped out: ‘You have become normal, sis.’
You walked towards me and put your arms around me.
I felt how you tried to hug me, like old times. But where you used to be soft, I now felt edges, bones: your shoulders, hips, collarbones; how your ribs floated rhythmically against my chest; your bra, and how it dented.
‘Thank you, brother,’ you said.

### Matroesjka – ikvorm (Dutch Original, 1st person)

Wat lijkt ze toch op oma,’ zeiden ze met Pasen, verjaardagen en Pinksteren, als ze je zagen spelen in de tuin. Je droeg de oude rokken en jurkjes van je nichten, die ook op oma leken, maar niet zoveel als jij. Er is een foto in een fotoboek in de archiefkast boven, waarop alle nichtjes in op elkaar lijkende blauwe jurkjes rondom oma zitten. Op haar verjaardag gemaakt. Alle meisjes kijken netjes zoals het hoort snoezig in de camera. *Cheese*.

Maar jij en oma niet. Jullie kijken ondeugend, alsof oma je net heeft verteld dat ze ontdekt heeft waar opa zijn snoeppot bewaart. Twee paar rechte neuzen met vlak voor het puntje een klein dalletje, vier helderblauwe ogen, oma’s witte haren die onder haar hoofddoekje uit piepen, jouw mond een klein beetje open, nog geen idee van wat poseren is, twee paar appelwangetjes, gloeiend als luciferkopjes. Je herinnert je het niet meer, je was pas drie toen oma stierf en van voor je vierde herinner je je niks, las ik laatst. Maar ik snap het gewoon niet, als ik naar die foto kijk snap ik het gewoon niet. Oma zo mooi, jij zo mooi – jullie waren anders, bijzonder.

Toch ben ik trots op je, zusje. Je besloot het en deed het; viel af als een matroesjka. Maar het lijkt wel alsof ieder beetje vet dat jouw lichaam uit verdwijnt wat meeneemt van wat jou zo jou maakt. Je verandert. Toen papa en mama laatst een weekend weg waren, vroeg je bij de snackbar of ze niet ook salade hebben. ’s Avonds keek je op jouw kamer film, met een vriend. Ik hoorde jullie door de muur heen lachen. Je stem is hoger tegenwoordig.

Toen we met Pasen met z’n allen eieren zochten in oma’s – nu tante Liesbeths – tuin, gaf je alle eitjes die je vond aan mij. Zelfs je lievelings, de witte met praline.
‘Jij kan het wél hebben,’ zei je, toen je ze in mijn handen stopte.
En voordat ik het wist was het eruit: ‘Je bent normaal geworden, zusje.’
Je liep op me af en sloeg je armen om me heen.
Ik voelde hoe je me probeerde te knuffelen, zoals vroeger. Maar waar je vroeger zacht was, voelde ik nu randen, botten: je schouders, heupen, sleutelbeenderen; hoe je ribben ritmisch tegen mijn borstkas zweefden; je beha, en hoe die indeukte.
‘Dankjewel, broer,’ zei jij.

### Matryoshka – he/she-form (English Translation, 3rd person)

‘She really looks like grandma,’ they said on Easter, birthdays and Pentecost, when they saw his sister playing in the garden. She wore the old skirts and dresses from her cousins, who looked like grandma as well, but not as much as she did. There is a picture in a photo book in the filing cabinet above, showing all cousins sitting around grandma in similar looking blue dresses. It has been taken on grandma's birthday. Every girl looks adorably into the camera as they should. *Cheese*.

Except her and grandma. They both look naughty, as if grandma just has told her that she has discovered where grandpa keeps his candy jar. Two pairs of straight noses with a small valley close to the tip, four bright blue eyes, grandmother's white hair sticking out from under her headscarf, her mouth slightly open, no idea yet what posing means, two pairs of apple cheeks, glowing as match heads. She doesn't remember it, she was only three when grandma died and he recently read that you don't remember anything before the age of four. But he just doesn't get it, when he looks at that picture he just doesn't get it. Grandma so beautiful, she so beautiful - you were different, special.

Yet he's proud of her, his sister. She decided it and she did it; she lost weight like a matryoshka. But it seems like every bit of fat that she loses takes some of her what defines her. She's changing. When mom and dad were away for a weekend lately, she asked for a salad at the snack bar. In the evening, she watched a movie in her room with a friend. He could hear them laughing through the wall. Her voice is higher nowadays.

During the easter egg hunt in grandma's - now aunt Liesbeth's - garden, she gave all the eggs she found to him. Even her favorites, the white ones containing praline.
‘You can handle it,’ she said, when she put them in his hands.
Before he knew it, it just slipped out: ‘You have become normal, sister.’
She walked towards him and put her arms around him.
He felt how she tried to hug him, like old times. But where she used to be soft, he now felt edges, bones: her shoulders, hips, collarbones; how her ribs floated rhythmically against his chest; her bra, and how it dented.
‘Thank you, brother,’ she said.

### Matroesjka – hij/zij-vorm (Dutch Original, 3rd person)

‘Wat lijkt ze toch op oma,’ zeiden ze met Pasen, verjaardagen en Pinksteren, als ze zijn zusje zagen spelen in de tuin. Ze droeg de oude rokken en jurkjes van haar nichten, die ook op oma leken, maar niet zoveel als zij. Er is een foto in een fotoboek in de archiefkast boven, waarop alle nichtjes in op elkaar lijkende blauwe jurkjes rond oma zitten. Op oma’s verjaardag gemaakt. Alle meisjes kijken netjes zoals het hoort snoezig in de camera. *Cheese*.

Maar zij en oma niet. Zij kijken ondeugend, alsof oma haar net heeft verteld dat ze ontdekt heeft waar opa zijn snoeppot bewaart. Twee paar rechte neuzen met vlak voor het puntje een klein dalletje, vier helderblauwe ogen, oma’s witte haren die onder haar hoofddoekje uit piepen, haar mond een klein beetje open, nog geen idee van wat poseren is, twee paar appelwangetjes, gloeiend als luciferkopjes. Ze herinnert zich het niet meer, ze was pas drie toen oma stierf en van voor je vierde herinner je je niks, las hij laatst. Maar hij snapt het gewoon niet, als hij naar die foto kijkt snapt hij het gewoon niet. Oma zo mooi, zij zo mooi – jullie waren anders, bijzonder.

Toch is hij trots op haar, zijn zusje. Ze besloot het en deed het; viel af als een matroesjka. Maar het lijkt wel, alsof ieder beetje vet dat haar lichaam uit verdwijnt, wat meeneemt van wat haar zo haar maakt. Ze verandert. Toen papa en mama laatst een weekend weg waren, vroeg ze bij de snackbar of ze niet ook salade hebben. ’s Avonds keek ze op haar kamer film, met een vriend. Hij hoorde ze door de muur heen lachen. Haar stem is hoger tegenwoordig.

Toen ze met Pasen met z’n allen eieren zochten in oma’s – nu tante Liesbeths – tuin, gaf ze alle eitjes die ze vond aan hem. Zelfs haar lievelings, de witte met praline.
‘Jij kan het wél hebben,’ zei ze, toen ze ze in zijn handen stopte.
En voordat hij het wist was het eruit: ‘Je bent normaal geworden, zusje.’
Ze liep op hem af en sloeg haar armen om hem heen.
Hij voelde hoe ze hem probeerde te knuffelen, zoals vroeger. Maar waar ze vroeger zacht was, voelde hij nu randen, botten: haar schouders, heupen, sleutelbeenderen; hoe haar ribben ritmisch tegen zijn borstkas zweefden; haar beha, en hoe die indeukte.
‘Dankjewel, broer,’ zei zij.

## Story 2: Koffiemolen by Martin Rombouts

### **Coffee grinder**. (English Translation, 1st person)

There are seven coffee machines in the kitchen of my dorm in Utrecht:

- Two filter coffee machines (old) (dirty) (sticky dust layer) (nobody ever uses it) (are they still working?)

- Three percolators (small ones) (for holidays, to put it on the camping stove?) (all three spotless) (never been used?)

- A Philips *Senseo II* Senseo machine (from those days where beautiful people were acting in coffee pad commercials) (I use it)

- A Krups *Fastspresso* Nespresso machine (*What else?*) (using those little cups) (the others use it)

- and a *real* espresso machine (with a seventies look) (and a little pointer indicating the pressure or something) (you have to put the coffee in a porta filter and press it with the perfect amount of force using some big, shiny chess pawn) (according to Naut, the only one who knows how to use it).

Naut tries it on once a month or so. We’re all sitting at the kitchen table in the morning, and I'm reading the newspaper, and then I grab my cup without looking and I take a sip, and it suddenly turns out to be a different kind of coffee.

I drop my newspaper and there’s Naut's face: ‘Shocking difference, isn't it? That trash of yours compared to an *espressolungo* made from Guatemalan Arabica beans. You taste the difference, right?’

‘Yes,’ I’ll say, ‘I do taste the difference, I just don't taste *thirty cents* of difference.’, and then he’ll look at me with a pitying, sorrowful kind of expression. As if I just told him that I don't believe in God, and he’s picturing me burning in everlasting fire.

To make Naut happy, and to show him that I'm really not indifferent to his great love, I bought an antique-looking and loudly squeaking coffee grinder for seven euros at a flea market in Rotterdam a few weeks ago.
The salesman was asking ten euros for it, but when I told him I only had seven euros, he nodded and agreed.

It’s sometimes said that after proper negotiations both parties feel like they have swindled the other. That was definitely the case here, as he didn’t make any fuss about me paying with a twenty euro note. At home, a closer inspection of my purchase revealed that the loud squeaking and creaking was caused by a sticky layer of green mold on the mill's grinder. It would be almost impossible to remove that mold, because the only access route was the narrow slit in front of the drawer that collects the coffee.

That evening, my cousin Katinkel came round; wine, Japanese snacks, laughter, that kind of thing. When I returned to the kitchen after getting us a second bottle of Aldi-shiraz - we were going out later - I saw her staring at my failed purchase, mesmerized. She asked why I owned a coffee grinder. I briefly told her about the squeaking and creaking, and about the mold that caused it.

Katinkel listened and turned the grinder.

Sure enough, it squeaked and creaked.

Katinkel picked up a nut. ‘Can I grind a peanut?’

‘What?’ I asked.

She took the peanut out of its coating. ‘In your grinder? A peanut.’

‘Sure, whatever.’

Katinkel turned the grinder with much squeaking and creaking. After that she looked in the drawer: finely ground peanut crumbs mixed with green dots of mold. She peeled another peanut, and pulled this one through the grinder as well; then she did it again. And again, and then she used two peanuts at once. Then even three and four peanuts at the same time. And then I said I wanted to try it too.

I threw five peanuts at the same time into the grinder, without taking off their coating. It barely fit. The grinder squeaked and creaked horribly, much louder than before. Apparently, it was so loud that the sound reached Naut’s room through the kitchen ceiling, making him run down the stairs into the kitchen to see what on earth was going on. He saw me there, trying to grind Japanese rice snacks in a groaning antique coffee grinder.

‘Oh you dirty Senseo drinker,’ Naut said. You savage! You have no heart, you. I knew it.' He picked up the coffee grinder and ripped it from my hands with such force that the coffee-collecting drawer became detached from its slit and flew through the kitchen, the salty snacks crumbs mixed with mold were spread across our kitchen floor. ‘Never, I'm never making you real coffee again.’ He ran back up the stairs to his room and closed the door with a loud bang.

I tried to explain it to him - via text, Facebook, even voicemail. I told him about the mold, about the narrow slit and the conversation with the salesman. I even vacuumed all the peanut crumbs from the kitchen floor, but he still refuses to talk to me. He wrote on the bulletin board in the kitchen that he has taken the coffee grinder to keep it safe from me.

Recently, when he went home for the weekend, I wanted to search his room, but it turned out that all of a sudden he was keeping his door locked. Of all the spare keys, only his was missing from the box in the closet.

I’ve hung the coffee-collecting drawer on his door handle.

### **Koffiemolen**. *Ik*. (Dutch Original, 1st person)

In de keuken van mijn Utrechtse studentenhuis staan zeven koffiezetapparaten.

Dubbele punt:

- Twee filterkoffieapparaten (oud) (vies) (plakkerige stoflaag) (gebruikt niemand) (doen ze het nog?).

- Drie percolators (kleintjes) (voor op vakantie, voor op het campinggasje?) (alle drie brandschoon) (nooit gebruikt?).

- Een Philips *Senseo II* Senseoapparaat (uit de tijd dat er nog mooie mensen in de koffiepadreclames zaten) (gebruik ik).

- Een Krups *Fastspresso* Nespressoapparaat (*What else?*) (met van die cupjes) (gebruiken de anderen).

- en Een *echt* espressoapparaat (met jaren ’70 uiterlijk) (en een wijzertje dat aangeeft hoe hoog de druk is ofzo) (je moet de koffie in zo’n los handvat doen en precies hard genoeg aandrukken met een soort grote, glimmende schaakpion) (zegt Naut, die als enige weet hoe hij werkt).

Iedere maand probeert Naut het wel een keer. Dan zitten we ’s ochtends met z’n allen aan de keukentafel, en dan zit ik de krant te lezen, en dan pak ik zonder te kijken mijn kopje en dan neem ik een slokje, en dan is het opeens andere koffie.

Ik laat mijn krant zakken en dan is het gezicht van Naut daar: ‘Dat is schrikken dat verschil hè? Die rommel of een *espresso lungo* van Guatemalteekse arabicabonen. Dat verschil proef je wel even, hè?’

‘Ja,’ zeg ik dan, ‘dat verschil proef ik wel, maar ik proef gewoon geen *dertig* cent verschil. Snapje?’ en dan kijk hij me aan met een soort meewarig treurige blik. Alsof ik hem net verteld heb dat ik niet in God geloof, en hij mij in eeuwig vuur brandend voor zich ziet.

Om Naut vrolijk te maken, en hem te laten zien dat ik echt niet onverschillig sta tegenover zijn grote liefde, kocht ik een paar weken terug op een rommelmarkt in Rotterdam voor zeven euro een antiek uitziende en bij ronddraaien luid piep- en knarsende koffiemolen.

De verkoper vroeg er tien euro’s voor, maar toen ik zei dat ik maar zeven op zak had hij geknikt en ‘is ook goed’ gezegd.

Ze zeggen wel eens dat na goede onderhandelingen beide partijen het gevoel hebben de ander te hebben opgelicht. Ik denk dat dat toen heel erg aan de hand was, want de man maakte er geen probleem van dat ik met een twintig eurobiljet betaalde. Thuis, bij nadere inspectie van mijn aankoop, ontdekte ik dat dat het luide piep- en knarsen werd veroorzaakt door een plakkerige laag groene schimmel die het maalwerk van het molentje bekleedde. En dat het bijna onmogelijk zou zijn die schimmel te verwijderen, omdat de enige toegangsweg de smalle gleuf voor het koffieopvanglaatje was.

Die avond had ik mijn nicht Katinkel over de keukenvloer voor Japanse zoutjes en wijn en gezelligheid enzo. Toen ik terugkwam van het uit mijn kamer halen van een tweede fles Aldi-shiraz – we moesten nog uit – zag ik haar gebiologeerd naar mijn geflopte aankoop staren. Ze vroeg waarom ik een koffiemolen had. Kort vertelde ik over het piepen en knarsen, en over de schimmel die daarvan de oorzaak was.

Katinkel luisterde en draaide.

Het piep- en knarste inderdaad.

Katinkel pakte een zoutje. ‘Mag ik een pinda malen?’
‘Wat?’ vroeg ik.

Ze brak de pinda uit zijn Japanse jasje. ‘In je molen? Een pinda.’

‘Je doet maar.’

Onder luid gepiep- en knars draaide Katinkel de molen. Daarna keek ze in het laatje: fijn gemalen pindabrokjes vermengd met groene puntjes schimmel. En daarna brak ze weer een pinda vrij, en haalde ook die door de molen; daarna deed ze dat nog een keer. En daarna deed ze dat nog een keer, en daarna deed ze er twee. Daarna zelfs drie en vier. En toen zei ik dat ik ook wilde.

Vijf tegelijk gooide ik in de molen, zelfs zonder het jasje van ze af te pellen. Het paste maar net. Het molentje piep- en knarste verschrikkelijk, nog veel luider dan daarvoor. Blijkbaar zelfs zo luid dat het geluid door het plafond heen tot in de kamer van Naut reikte, die de trap af de keuken in kwam rennen om te kijken wat er in godsnaam aan de hand was. En daar zag hij mij die in een kreunende antieke koffiemolen handjes Japanse rijstzoutjes probeerde te vermalen.

‘O jij vuile Senseodrinker,’ zei Naut. ‘Barbaar! Jij hebt geen hart, jij. Ik wist het wel.’ Hij pakte de koffiemolen vast en trok hem met zoveel kracht uit mijn handen dat het koffie-opvanglaatje uit zijn gleuf losraakte en door de keuken vloog, de met schimmel vermengde zoutjeskruimels over onze keukenvloer uitzaaiend. ‘Nooit, nooit geef ik je meer echte koffie.’ Hij rende de trap op naar boven, naar zijn kamer; trok met een harde klap de deur dicht.

Ik heb het geprobeerd aan hem uit te leggen – via sms, Facebook, zelfs voicemail. Ik vertelde van de schimmel, van de smalle gleuf en het gesprek met de marktkoopman. Ik heb zelfs alle pindakruimels van de keukenvloer gestofzuigd, maar hij weigert nog steeds met me te praten. Op het mededelingenbord in de keuken schreef hij dat hij de koffiemolen tegen mij in bescherming heeft genomen.

Laatst, toen hij een weekend van huis was, wilde ik in zijn kamer op zoek te gaan, maar hij bleek zijn kamerdeur plots op slot te houden. Van de reservesleutels in het bakje in de kast, mist alleen de zijne.

Ik heb het opvanglaatje maar aan zijn deurklink gehangen.

### **Coffee grinder**. *She*. (English Translation, 3rd person)

There are seven coffee machines in the kitchen of her dorm in Utrecht.

Colon:

- Two filter coffee machines (old) (dirty) (sticky dust layer) (nobody ever uses it) (are they still working?).

- Three percolators (small ones) (for holidays, to put it on the camping stove?) (all three spotless) (never been used?).

- A Philips *Senseo II* Senseo machine (from the days when beautiful people were still playing in the coffee pads commercials) (She uses it).

- A Krups *Fastspresso* Nespresso machine (*What else?*) (using those little cups) (the others use it).

- and a *real* espresso machine (seventies look) (and a little pointer indicating the pressure or something) (you should do the coffee in a loose handle and press it with the perfect amount of force using a sort of big, shiny chess pawn) (according to Naut, the only one who knows how it works).

Naut is trying it once a month. Then they all sit together at the kitchen table in the morning, she's reading the newspaper, and then she grabs her cup without looking and she takes a sip, and then it suddenly turns out to be a different kind of coffee.

She drops her newspaper and then the face of Naut appears: ‘The difference is shocking, isn't? That stuff or an *espresso lungo* made from Guatemalan arabica beans. You can clearly taste the difference, right?’

‘Yes,’ she says then, ‘I taste the difference, but I just don't taste a difference of *thirty* cents. Get it?’ and then he looks at her with a kind of pitying sorrowful look. Like she just told him that she doesn't believe in God, and he picturing her burning in everlasting fire.

To make Naut happy, and to show him that she's really not indifferent to his great love, she bought an antique-looking and loud squeaking coffee grinder for seven euros at a flea market in Rotterdam a few weeks ago.
The salesman was asking ten euros for it, but when she told him she only had seven euros, he had nodded and agreed.

Sometimes they say that after proper negotiations both sides feel like they have been swindling the other person. Probably that was going on there, because it wasn't a problem that she paid with a twenty euro note. At home, a closer inspection of her purchase revealed that the loud squeaking and creaking was caused by a sticky layer of green mold on the mill's grinder. And that it would be almost impossible to remove that mold, because the only access route was the narrow slit in front of the drawer that absorbs the coffee.

That evening, her cousin Katinkel visited her for Japanese salty snacks and wine and having fun and everything. When she returned from her room to get a second bottle of Aldi-shiraz - they still had to go out - she saw her cousin staring mesmerized at her failed purchase. She asked why she owned a coffee grinder. She briefly told her about the squeaking and creaking, and about the mold that caused it.

Katinkel listened and turned the grinder.

It was squeaking and creaking indeed.

Katinkel took a salty snack. ‘May I grind a peanut?’

‘What?’

She peeled off the Japanese layer. ‘In your grinder? A peanut.’

‘Whatever you want.’

Katinkel turned the grinder with much squeaking and creaking. After that she looked in the drawer: finely ground peanut crumbs mixed with green dots of mold. And then she peeled off another peanut, and pulled this one through the grinder as well; and thereafter she did it again. And again, and then she used two peanuts at once. Even three and four peanuts after that. And then her cousin said she wanted to.

She threw five peanuts at the same time in the grinder, even without shelling. It just fit. The grinder squeaked and creaked horribly, much louder than before. Apparently, it was so loud that the sound reached Naut through the ceiling in his room. He ran down the stairs into the kitchen to see what on Earth was going on. And there he saw his roommate trying to grind Japanese rice snacks in a groaning antique coffee grinder.

‘Oh you dirty Senseo drinker,’ Naut said. 'Barbarian! You have no heart, you. I knew it.' He picked up the coffee grinder and ripped it from her hands with such force that the coffee absorbing drawer became detached from it's slit and flew through the kitchen, the salty snacks crumbs mixed with mold were spread across the kitchen floor. ‘Never, I will never give you real coffee any longer.’ He ran up the stairs to his room; he closed the door with a loud bang.

She tried to explain it to him - via text messaging, Facebook, even voicemail. She told him about the mold, about the narrow slit and the conversation with the salesman. She even vacuumed all peanut crumbs from the kitchen floor, but he still refuses to talk to her. He wrote on the bulletin board in the kitchen that he has taken the coffee grinder to protect it from her.

Lately, when he was away for the weekend, she wanted to search his room, but it turned out he was keeping his bedroom door locked suddenly. Of all spare keys, only his was missing from the box in the closet.

She just hung the coffee absorbing drawer on his door handle.

### **Koffiemolen**. *Zij.* (Dutch Original, 3rd person)

In de keuken van haar Utrechtse studentenhuis staan zeven koffiezetapparaten.

Dubbele punt:

- Twee filterkoffieapparaten (oud) (vies) (plakkerige stoflaag) (gebruikt niemand) (doen ze het nog?).

- Drie percolators (kleintjes) (voor op vakantie, voor op het campinggasje?) (alle drie brandschoon) (nooit gebruikt?).

- Een Philips *Senseo II* Senseoaparaat (uit de tijd dat er nog mooie mensen in de koffiepadreclames zaten) (gebruikt zij).

- Een Krups *Fastspresso* Nespressoapparaat (*What else?*) (met van die cupjes) (gebruiken de anderen).

- en Een *echt* espressoapparaat (met jaren ’70 uiterlijk) (en een wijzertje dat aangeeft hoe hoog de druk is ofzo) (je moet de koffie in zo’n los handvat doen en precies hard genoeg aandrukken met een soort grote, glimmende schaakpion) (zegt Naut, die als enigeweet hoe hij werkt).

Iedere maand probeert Naut het wel een keer. Dan zitten ze ’s ochtends met z’n allen aan de keukentafel, en dan zit zij de krant te lezen, en dan pakt ze zonder te kijken haar kopje en dan neemt ze een slokje en dan: is het opeens andere koffie.

Ze laat haar krant zakken en dan is het gezicht van Naut daar: ‘Dat is schrikken dat verschil hè? Die rommel of een *espresso lungo* van Guatemalteekse arabicabonen. Dat verschil proef je wel even, hè?’

‘Ja,’ zegt ze dan, ‘dat verschil proef ik wel, maar ik proef gewoon geen *dertig* cent verschil. Snapje?’ en dan kijk hij haar aan met een soort meewarig treurige blik. Alsof ze hem net verteld heeft dat ze niet in God gelooft, en hij haar in eeuwig vuur brandend voor zich ziet.

Om Naut vrolijk te maken, en hem te laten zien dat ze echt niet onverschillig staat tegenover zijn grote liefde, kocht ze een paar weken terug op een rommelmarkt in Rotterdam voor zeven euro een antiek uitziende en bij ronddraaien luid piep- en knarsende koffiemolen.

De verkoper vroeg er tien euro’s voor, maar toen ze zei dat ze maar zeven op zak had hij geknikt en ‘is ook goed’ gezegd.

Ze zeggen wel eens dat na goede onderhandelingen beide partijen het gevoel hebben de ander te hebben opgelicht. Vermoedelijk was dat toen heel erg aan de hand, want de man maakte er geen enkel probleem van dat ze met een twintig eurobiljet betaalde. Thuis, bij nadere inspectie van haar aankoop, ontdekte ze dat het luide piep- en knarsen werd veroorzaakt door een plakkerige laag groene schimmel die het maalwerk van het molentje bekleedde. En dat het bijna onmogelijk zou zijn die schimmel te verwijderen, omdat de enige toegangsweg de smalle gleuf voor het koffieopvanglaatje was.

Die avond had ze haar nicht Katinkel over de keukenvloer voor Japanse zoutjes en wijn en gezelligheid enzo. Toen ze terugkwam van het uit haar kamer halen van een nieuwe fles Aldi-shiraz – ze moesten nog uit – zag ze haar nicht gebiologeerd naar haar geflopte aankoop staren. Ze vroeg waarom ze een koffiemolen had. Kort vertelde ze over het piepen en knarsen, en over de schimmel die daarvan de oorzaak was.

Katinkel luisterde en draaide.

Het piep- en knarste inderdaad.

Katinkel pakte een zoutje. ‘Mag ik een pinda malen?’
‘Wat?’

Ze brak de pinda uit zijn Japanse jasje. ‘In je molen? Een pinda.’

‘Je doet maar.’

Onder luid gepiep- en knars draaide Katinkel de molen. Daarna keek ze in het laatje: fijn gemalen pindabrokjes vermengd met groene puntjes schimmel. En daarna brak ze weer een pinda vrij, en haalde ook die door de molen; daarna deed ze dat nog een keer. En daarna deed ze dat nog een keer, en daarna deed ze er twee. Daarna zelfs drie en vier. En toen zei haar nicht dat ze ook wilde.

Vijf tegelijk gooide ze in de molen, en zelfs zonder er het rijstjasje vanaf te pellen. Het paste maar net. Het molentje piep- en knarste verschrikkelijk, nog veel luider dan daarvoor. Blijkbaar zelfs zo luid dat het geluid door het plafond heen tot in de kamer van Naut reikte, die de trap af de keuken in kwam rennen om te kijken wat er in godsnaam aan de hand was. En daar zag hij zijn huisgenote die een kreunende antieke koffiemolen handjes Japanse rijstzoutjes probeerde te vermalen.

‘O jij vuile Senseodrinker,’ zei Naut. ‘Barbaar! Jij hebt geen hart, jij. Ik wist het wel.’ Hij pakte de koffiemolen vast en trok hem met zoveel kracht uit haar handen dat het koffie-opvanglaatje uit zijn gleuf losraakte en door de keuken vloog, de met schimmel vermengde zoutjeskruimels over de keukenvloer uitzaaiend. ‘Nooit, nooit geef ik je meer echte koffie.’ Hij rende de trap op naar boven, naar zijn kamer; trok met een harde klap de deur dicht.

Ze heeft het geprobeerd aan hem uit te leggen – via sms, Facebook, zelfs voicemail. Ze vertelde van de schimmel, van de smalle gleuf en het gesprek met de marktkoopman. Ze heeft zelfs alle pindakruimels van de keukenvloer gestofzuigd, maar hij weigert nog steeds met haar te praten. Op het mededelingenbord schreef hij dat hij de koffiemolen tegen haar in bescherming heeft genomen.

Laatst, toen hij een weekend van huis was, wilde ze in zijn kamer op zoek te gaan, maar hij bleek zijn kamerdeur plots op slot te houden. Van de reservesleutels in het bakje in de kast, mist alleen de zijne.

Ze heeft het opvanglaatje maar aan zijn deurklink gehangen.

## Story 3: Meesterwerk by Martin Rombouts

### Meesterwerk by Martin Rombouts (English Translation, only ‘he’-version)

He opened the window in the conservatory of his spacious country house by touch, with his eyes closed. He's seeing everything in red due to the blood in his eyelids. Would there be clouds? Will he recognize his feelings in the clouds? A breeze is blowing inside next to him. He sniffs. Could you smell weather? And would this be the smell of cumulus clouds? He covers his eyes with his hands and puts his feet firmer on the ground. Now he's seeing everything in black. He relaxes his shoulders then. He notices that they were tense. He breaths in. Breaths out. And when he's breathing in again, he does it: he puts his hands down and opens his eyes wide as he can.

It burns, he's only seeing white, but he continues. Until he sees everything, and closes the window again.

The phone's ringing and he feels guilty. Not because he doesn't answer the phone, but because he has been disappointed. Maybe he shouldn't have been expecting so much. But should he just take everything as it is? May he not hope?! May he not try to make the best out of each new day? He's furious and slams the window, closed by the curtain again. It's emitting a cloud of dust. He'll let Anastasia know next time. When would she come again? What day is it today actually?

The phone's ringing again.

- Hello, he says.

- Where are you?

- Well, he says, just where I am.

- Are you at home?

- Behind my desk.

- We had ...

- My fancy desk, not that ugly thing.

- ... We were going to meet. You wanted to talk to me today.

- Suusje, I wanted so much more for you. How could I know thay you would suddenly listen to me now?

- Aw man, not that again.

- Father, Suus. I'm your father, not your husband.

- Father. They say they havent't seen you here in weeks. That Johannes is making the decisions now.

- Johannes, he's a wise man.

- You hate Johannes.

- He could have been my son in law, Suus. Remember?

- That's ... Dad. What do you want? ... Has something happened? Are you on holiday or what...

- Suus, I became a painter.

Decisively, he hangs up the phone, and walks away from the desk. Towards the corner with the best light, close to the window. From there he takes a look at his creation displayed on the easel. He knows that the people outside think he's crazy, but whatever, let them stay outside. Their letters and numbers on paper, their culture of dialogue, their tax strategies, that's not what it's all about. This, what he's creating, that's what matters, this. White on white, on blank canvas. About the relief. Differences in height. Paint on paint on paint.

He bends over, brings his face close to the canvas until he's only seeing white. He closes his eyes and he sniffs. It smells good.

Then he turns his head and he's getting closer. He rubs the canvas with his cheek, with his beard, and using these hairs that he has never allowed himself before, he feels that it's fine. He's getting ready to add a new coat of paint.

## Story 4: Emotioneel by Martin Rombouts

### Emotioneel (English Translation, only ‘he’-version)

His grandmother was dying, and because his mother had criticized him earlier in the evening that he wasn't in contact with his feelings, he instructed his music player to play sad music only. His music player had a special setting for that: *SensMe Emotional*; and therefore it was easy to do. He could just go on thinking, while he was waiting for the confirmatory call.

Twenty, he believed, is actually a good age to have your grandparents dying. As being fifty is the right age to have your parents dying.

During the first ten years of your life, you depend on them; they depend on you during their last ten years.

During the second ten years, you envy them (they are grown up and can do everything they like; you can't); between their last twenty and ten years (when their eyelids start sagging) they envy you.

But the ten years in between you live in harmony. They associate their happiness with your success; you give them grandchildren in which they recognize their childhood photos.

Symmetry. Balance. Equality. His parents don't have to live past the age of seventy.

But what was he saying? He was in his room. He was lying on bed, looking at the moisture spots and cobwebs on the ceiling, while he was listening to the sad music as long as the wait lasted. That took longer than expected and when his mother called him it was already half past three in the morning. He was *Emotional* for at least 8 hours. He could even cry about it, and proudly weeping he thought: well done, you're in good contact with your feelings.

While the music player was still playing he fell asleep with his head on a wet pillow.

He can't tell you whether he was dreaming that night, and if so: what he was dreaming about. He doesn't remember.

But what he does know is that when he woke up the next morning, and the music player switched from *SensMe Emotional* to *SensMe Exuberant*, that his feelings didn't change back with it.
