# Supplementary material for: When Fiction Is Just as Real as Fact: No Differences in Reading Behavior between Stories Believed to be Based on True or Fictional Events
Source: Front Psychol. 2017 Sep 20;8:1618. doi: 10.3389/fpsyg.2017.01618 (PMC5613255; doi:10.3389/fpsyg.2017.01618)
Supplement: Supplementary file 2 [file DataSheet2.docx]

# S2: Story World Absorption scale

## English Translation

### Attention

1. While reading the story, I lost track of time.
2. During reading, I was focused on what happened in the story
3. I was immersed in the story during reading.
4. My attention was so focused on the story that I forgot about the surroundings.

### Emotional Engagement

1. I was able to understand the events in the story in a way similar to the way the characters understood them.
2. I could empathize with the characters.
3. I felt connected with the protagonist of this story.
4. I shared the emotions of the protagonist.
5. The story affected me emotionally.

### Transportation

1. I forgot my own problems and concerns during the story.
2. When I finished reading the story, it felt like I had travelled into the world in which the story was set.
3. While reading, it seemed as if I was inside the narrative world.
4. While reading, my body was in the room, but my mind was inside the world created by the story.
5. At times, the world of the story and reality seemed to overlap.

### Mental Imagery

1. While reading, I had an image of the main character in my mind.
2. While reading, I could see images of the situations being described.
3. At times, I could see the settings/environment in which the story unfolds in my mind.
4. At times, I had the feeling that I could see right through the eyes of the main character.

### Perspective:

1. At times, I had the feeling of seeing right through the eyes of the protagonist.
2. While listening to the story, I saw the situations which were described in my head as if I was an uninvolved observer.

## Dutch Original

### Attention

1. Tijdens het lezen van het verhaal vergat ik de tijd
2. Tijdens het lezen van het verhaal was ik geconcentreerd op wat er in het verhaal gebeurde
3. Ik voelde mij geabsorbeerd in het verhaal
4. Het verhaal pakte me zo dat ik me kon afsluiten voor wat er om mij heen gebeurde
5. Ik was zo geconcentreerd aan het lezen dat ik de wereld om mij heen even was vergeten

### Transportation

1. Tijdens het lezen van het verhaal leek het soms alsof ik zelf ook in de wereld van het verhaal was
2. Tijdens het lezen van het verhaal waren er momenten waarop de wereld van het verhaal leek te overlappen met mijn eigen wereld
3. De wereld van het verhaal voelde tijdens het lezen soms dichterbij dan de wereld om mij heen
4. Toen ik klaar was met lezen van het verhaal voelde het alsof ik net een uitstapje had gemaakt naar de wereld van het verhaal
5. Omdat al mijn aandacht uit ging naar het verhaal, leek het soms alsof ik niet meer los van het verhaal bestond

### Emotional Engagement

1. Ik kon me tijdens het lezen van het verhaal voorstellen hoe het zou zijn om in de schoenen van de hoofdpersoon te staan
2. Ik voelde met de hoofdpersoon in het verhaal mee
3. Ik voelde me verbonden met de hoofdpersoon in dit verhaal
4. Ik voelde mij hoe de hoofdpersoon zich voelde
5. Ik leefde mee met wat er gebeurde in het verhaal

### Mental Imagery

1. Tijdens het lezen van dit verhaal had ik een beeld van de hoofdpersoon voor mijn ogen
2. Tijdens het lezen van dit verhaal kon ik de situaties die beschreven werden voor me zien
3. Ik kon me voorstellen hoe de omgeving waarin het verhaal zich afspeelt eruit zag

### Perspective

1. Soms had ik het gevoel door de ogen van de hoofdpersoon te kunnen zien.
2. Tijdens het lezen zag ik situaties die beschreven werden voor me alsof ik een stille toeschouwer was.
