# Supplementary figures and images for: When Fiction Is Just as Real as Fact: No Differences in Reading Behavior between Stories Believed to be Based on True or Fictional Events
Source: Front Psychol. 2017 Sep 20;8:1618. doi: 10.3389/fpsyg.2017.01618 (PMC5613255; doi:10.3389/fpsyg.2017.01618)

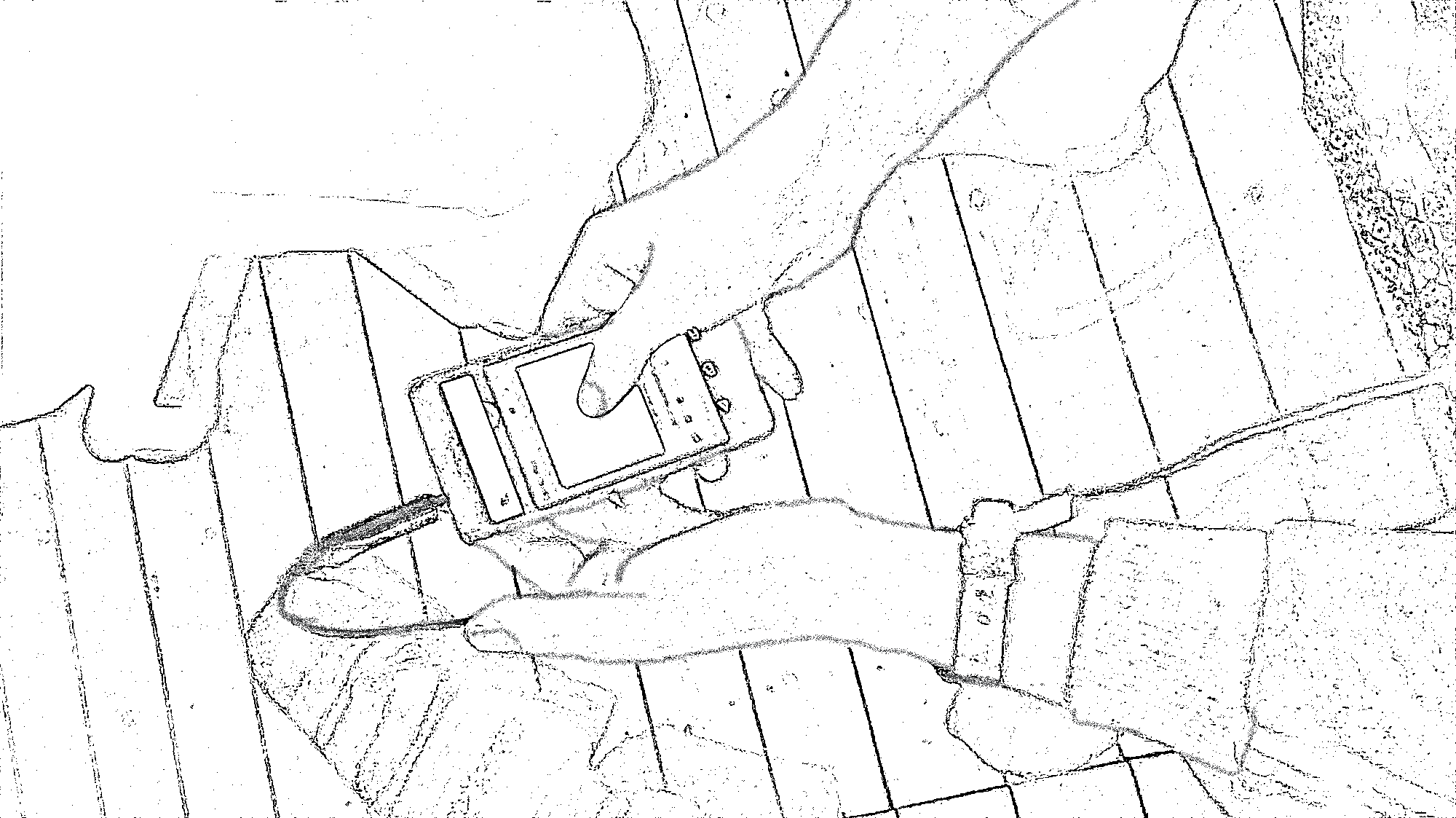

Supplement: Supplementary file 3 [file DataSheet3.ZIP › Emotioneel1_1stperson.png]

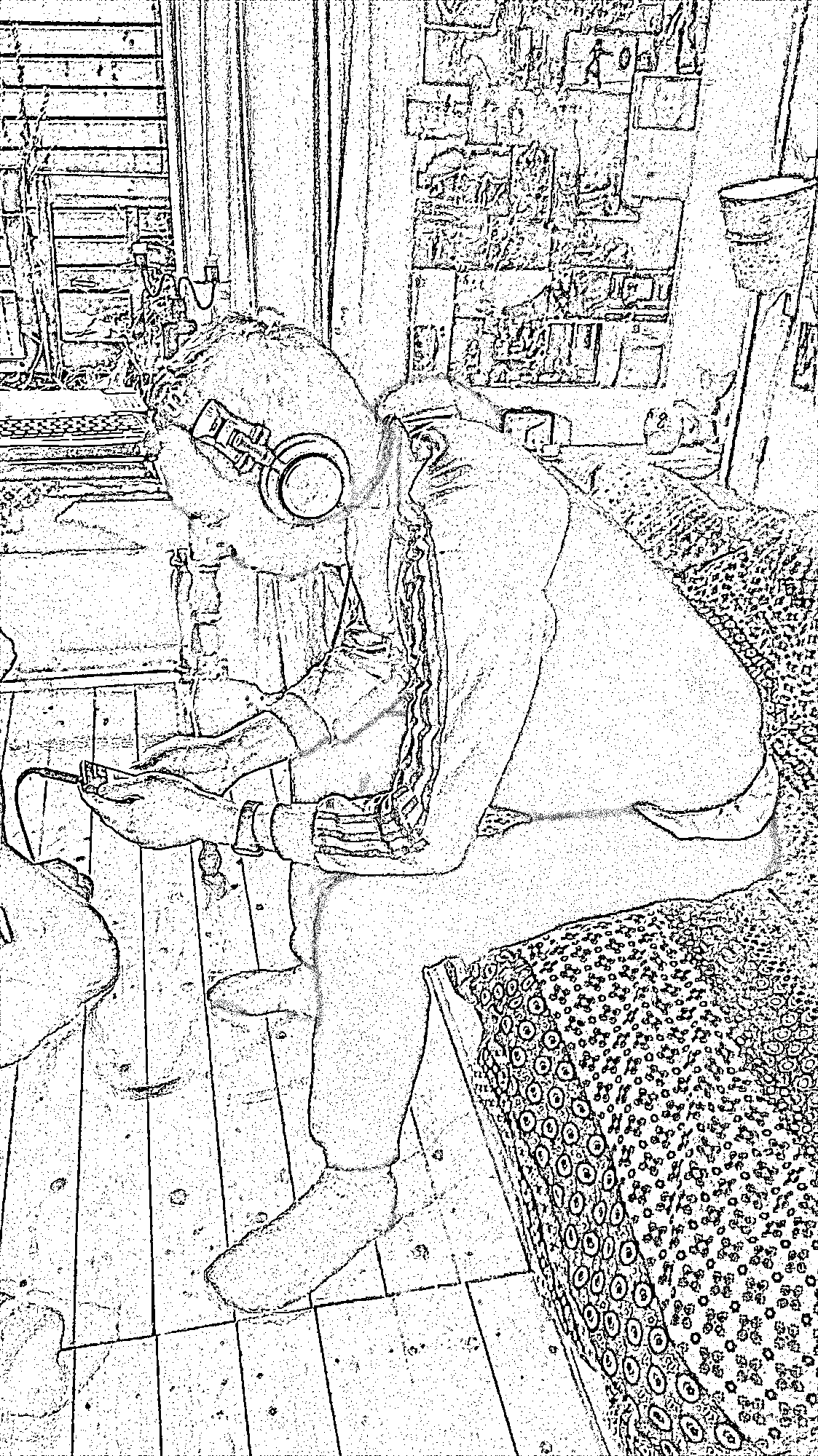

Supplement: Supplementary file 3 [file DataSheet3.ZIP › Emotioneel1_3rdperson.png]

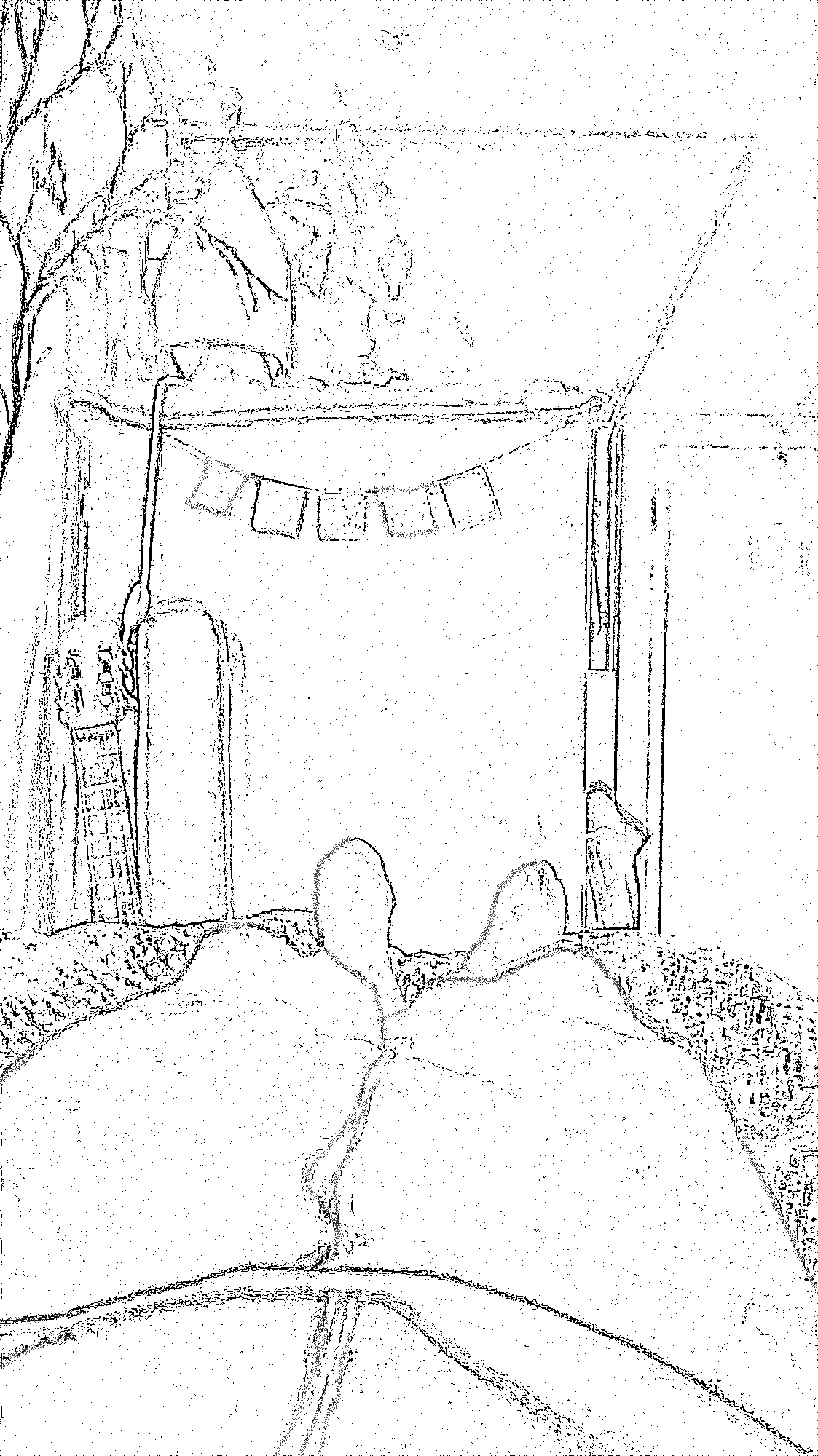

Supplement: Supplementary file 3 [file DataSheet3.ZIP › Emotioneel2_1stperson.png]

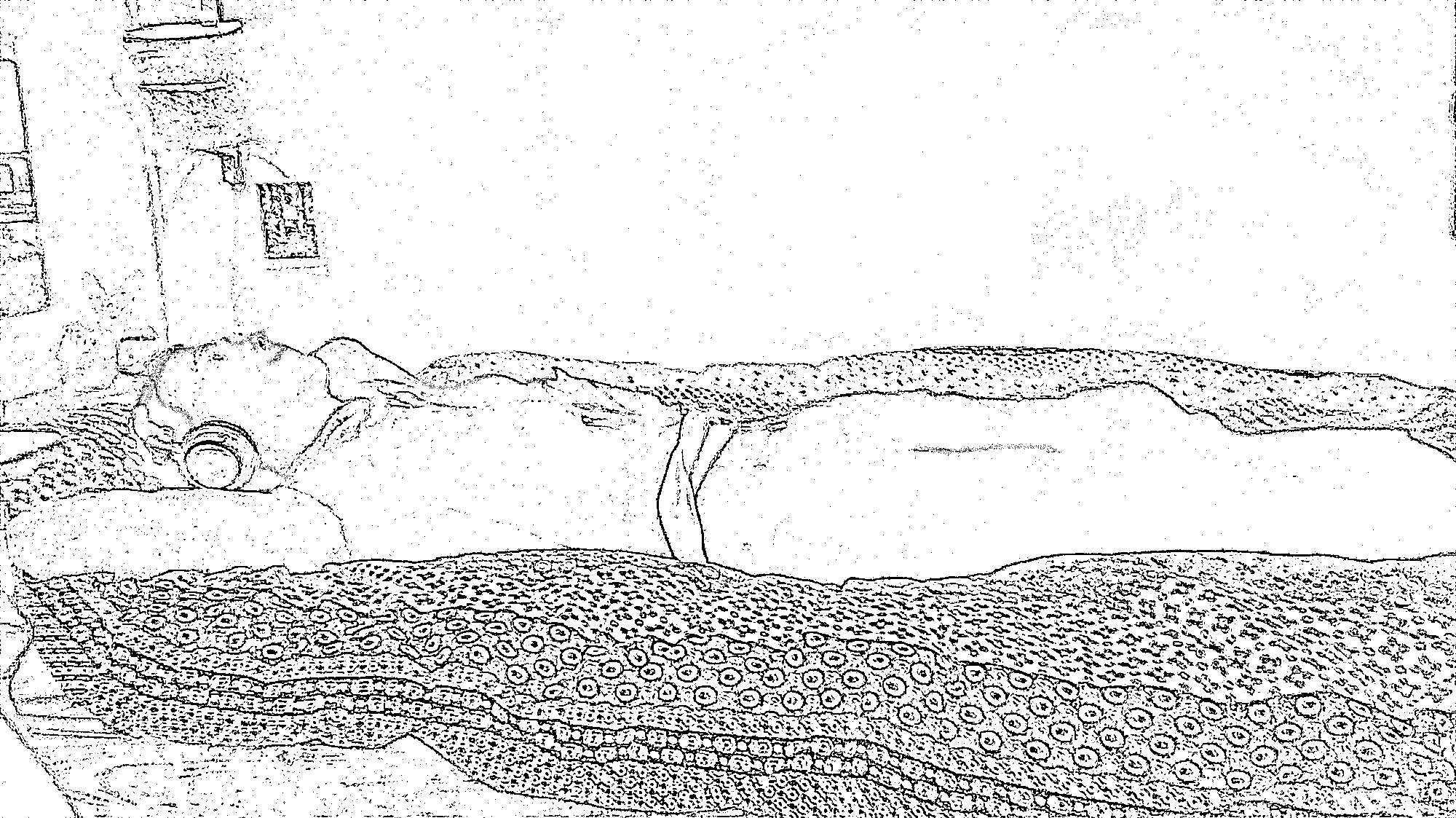

Supplement: Supplementary file 3 [file DataSheet3.ZIP › Emotioneel2_3rdperson.png]

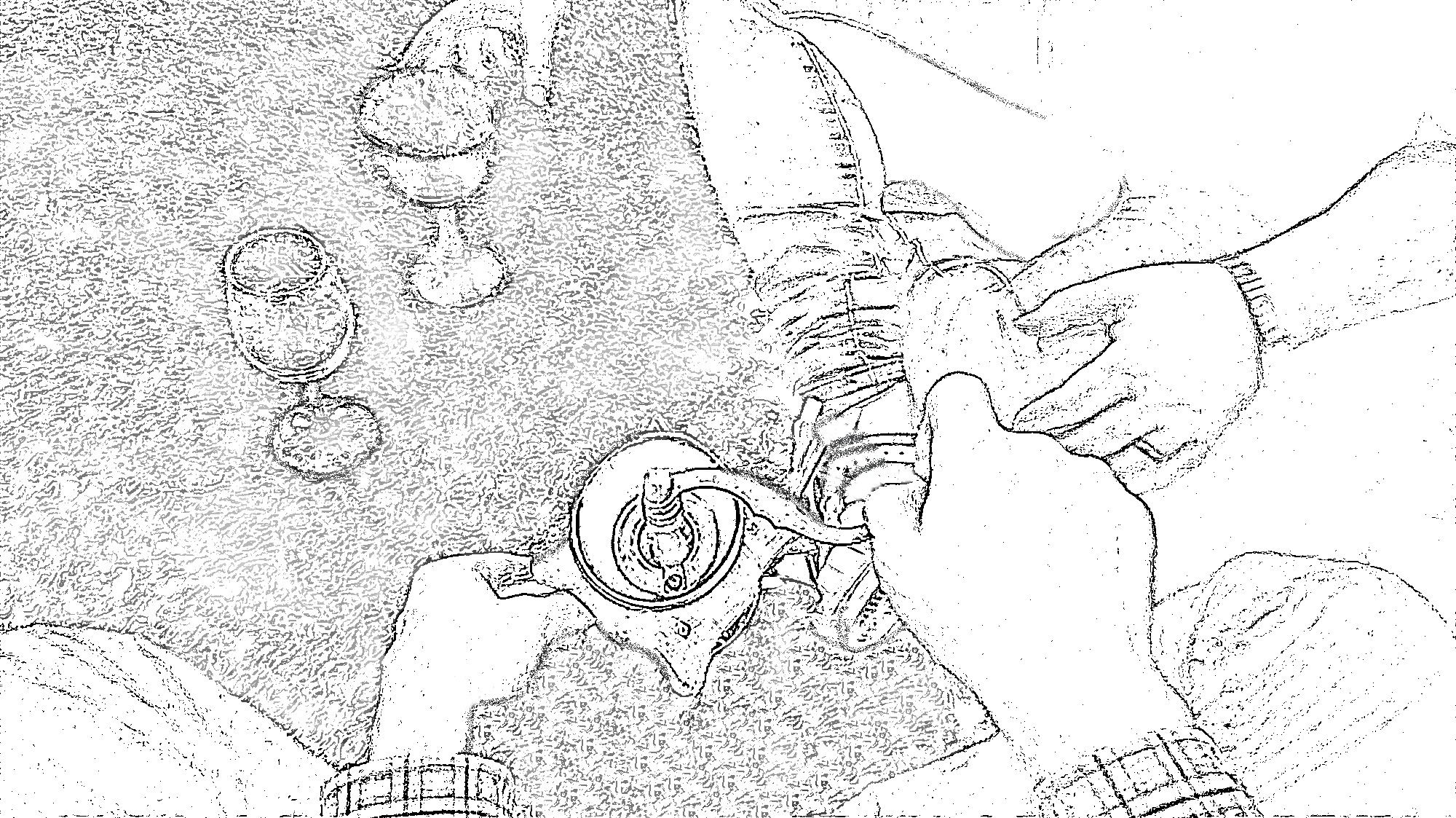

Supplement: Supplementary file 3 [file DataSheet3.ZIP › Koffiemolen1_1stperson.png]

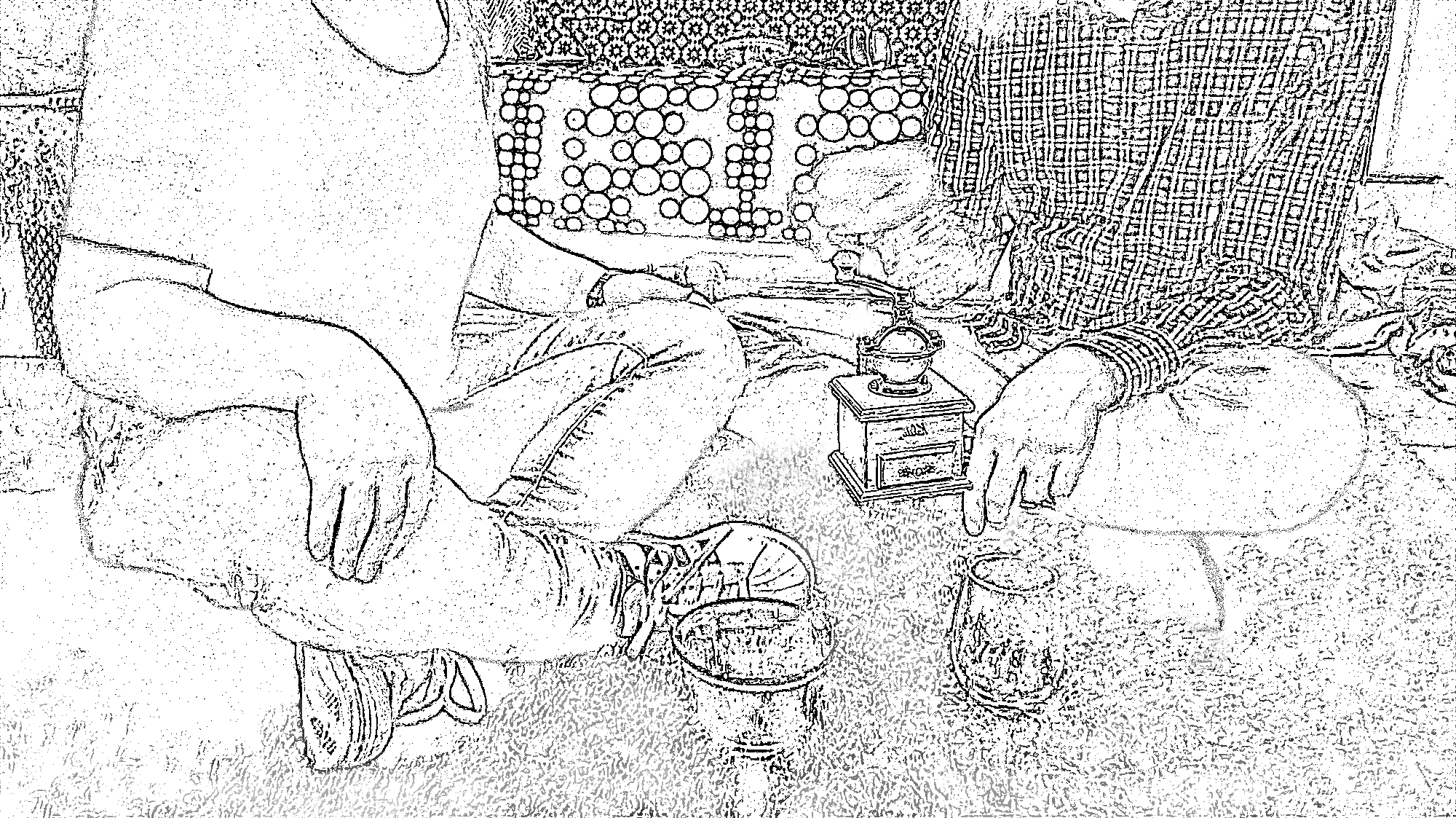

Supplement: Supplementary file 3 [file DataSheet3.ZIP › Koffiemolen1_3rdperson.png]

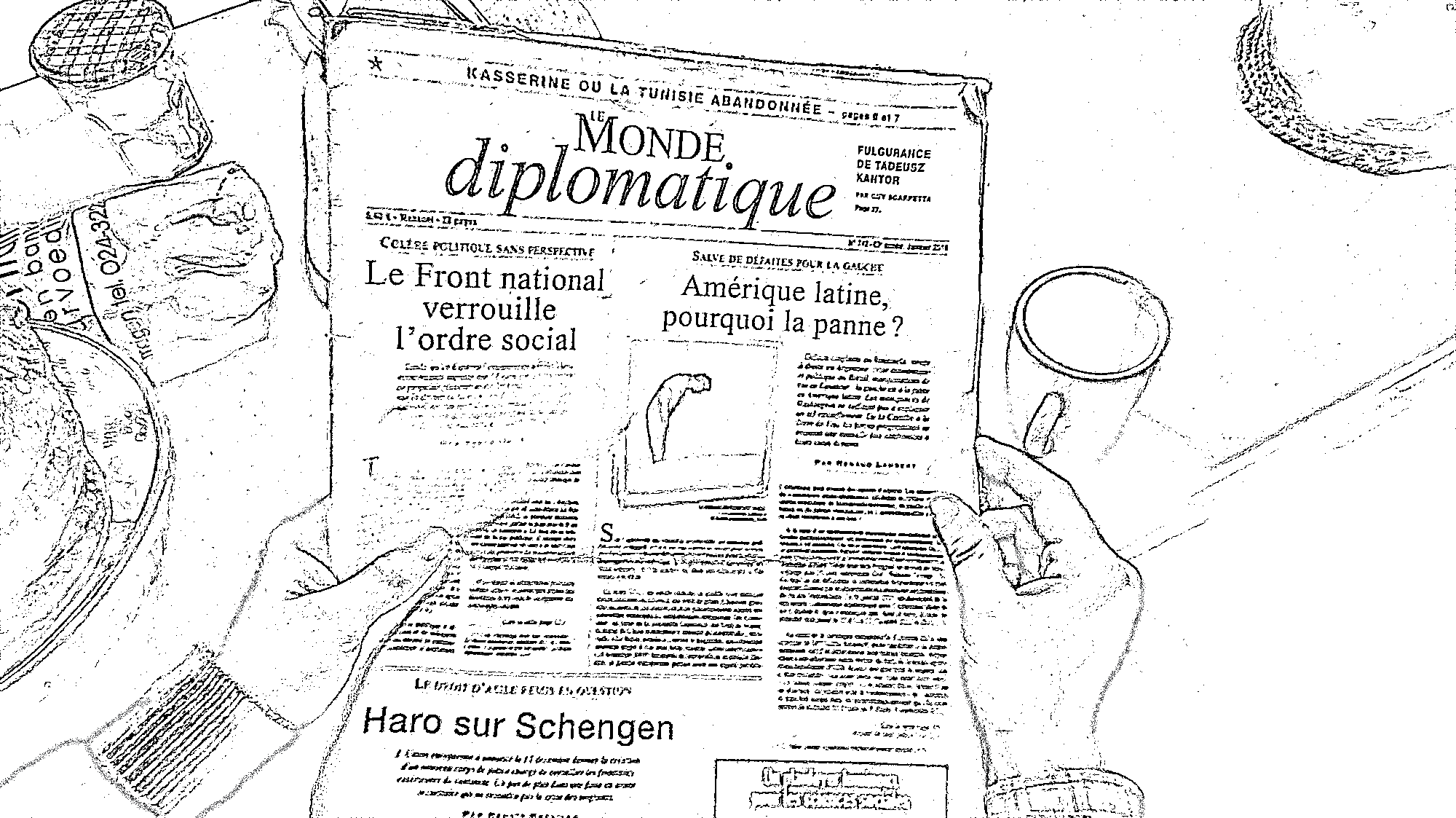

Supplement: Supplementary file 3 [file DataSheet3.ZIP › Koffiemolen2_1stperson.png]

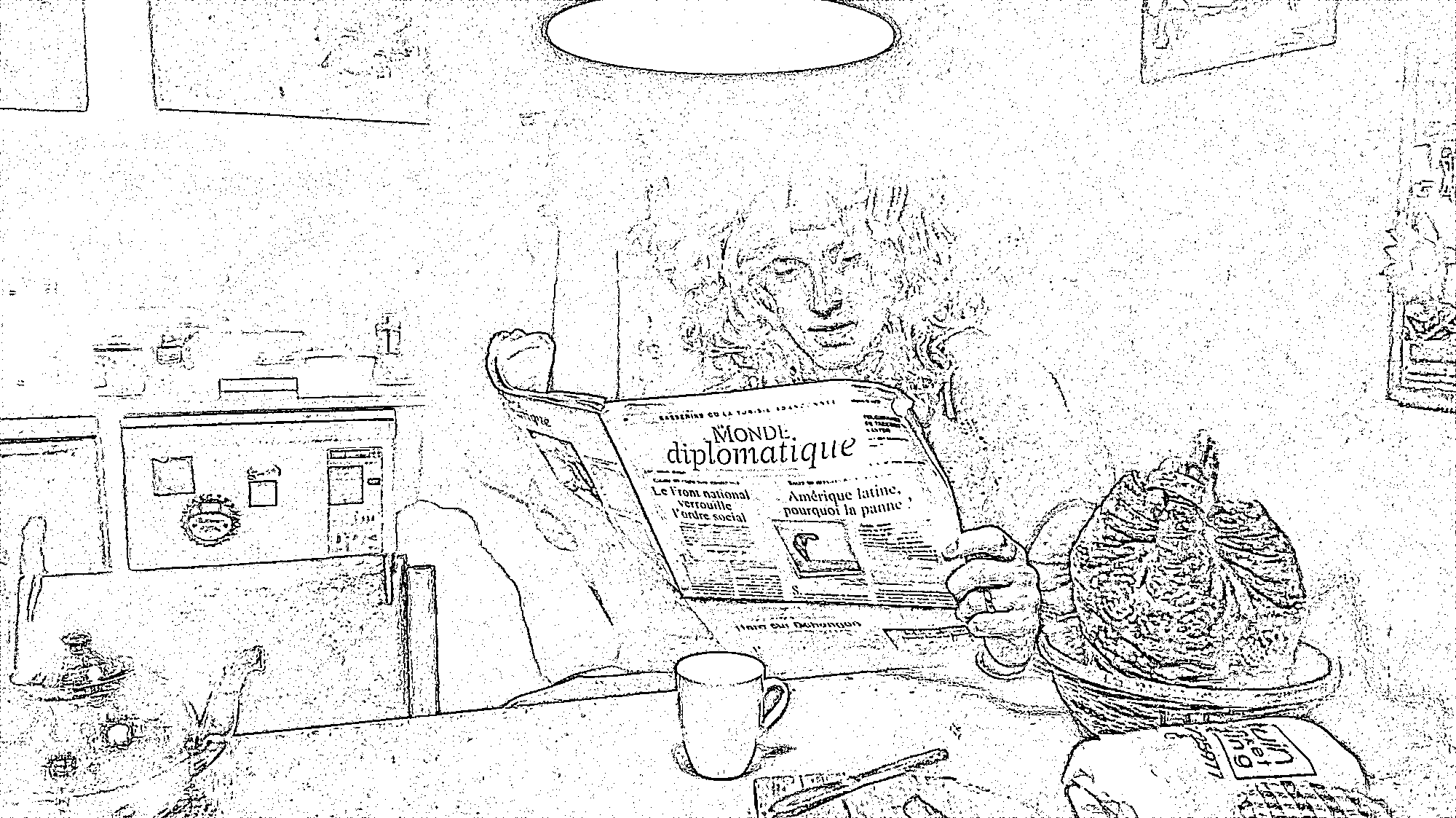

Supplement: Supplementary file 3 [file DataSheet3.ZIP › Koffiemolen2_3rdperson.png]

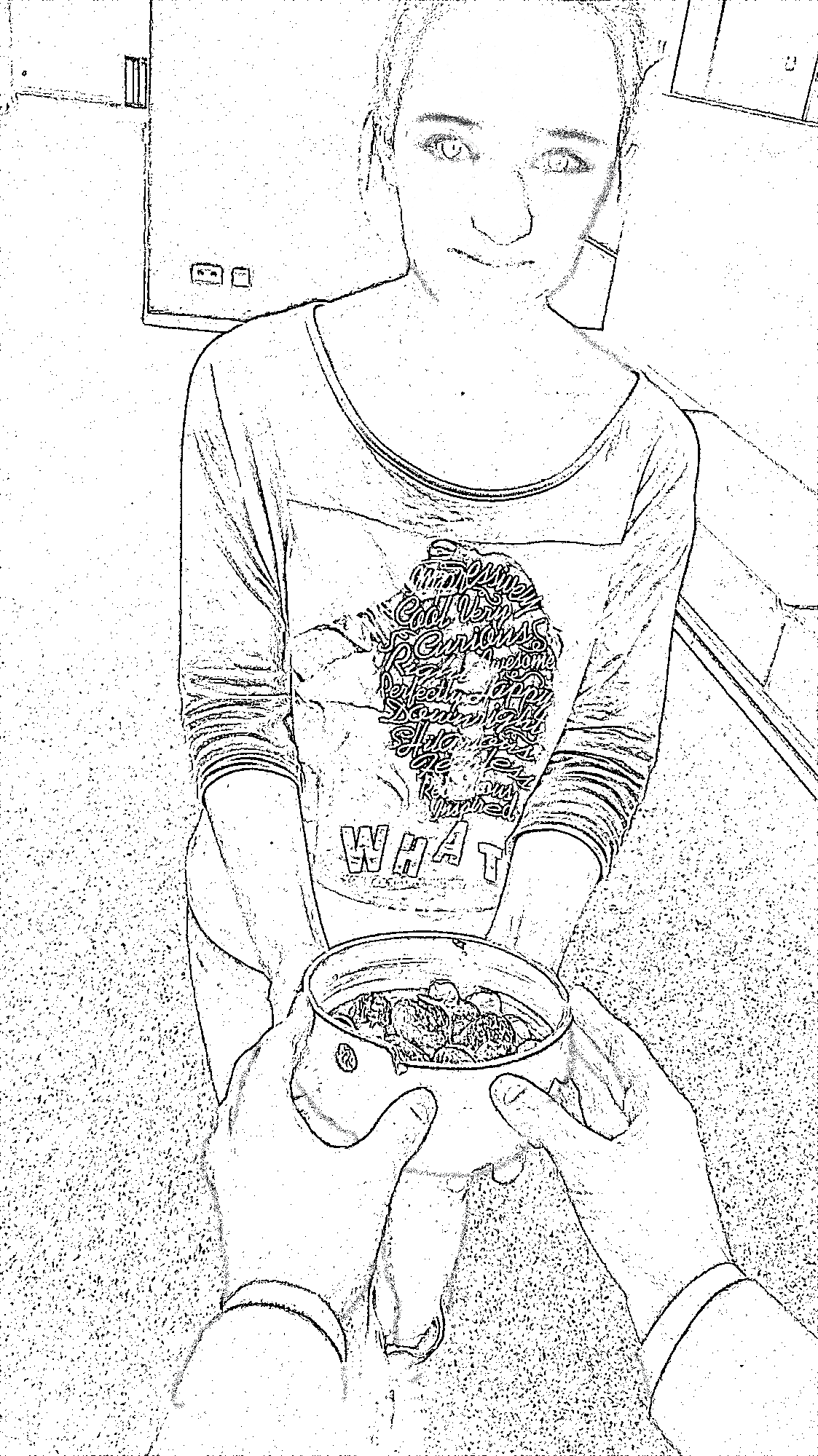

Supplement: Supplementary file 3 [file DataSheet3.ZIP › Matroesjka1_1stperson.png]

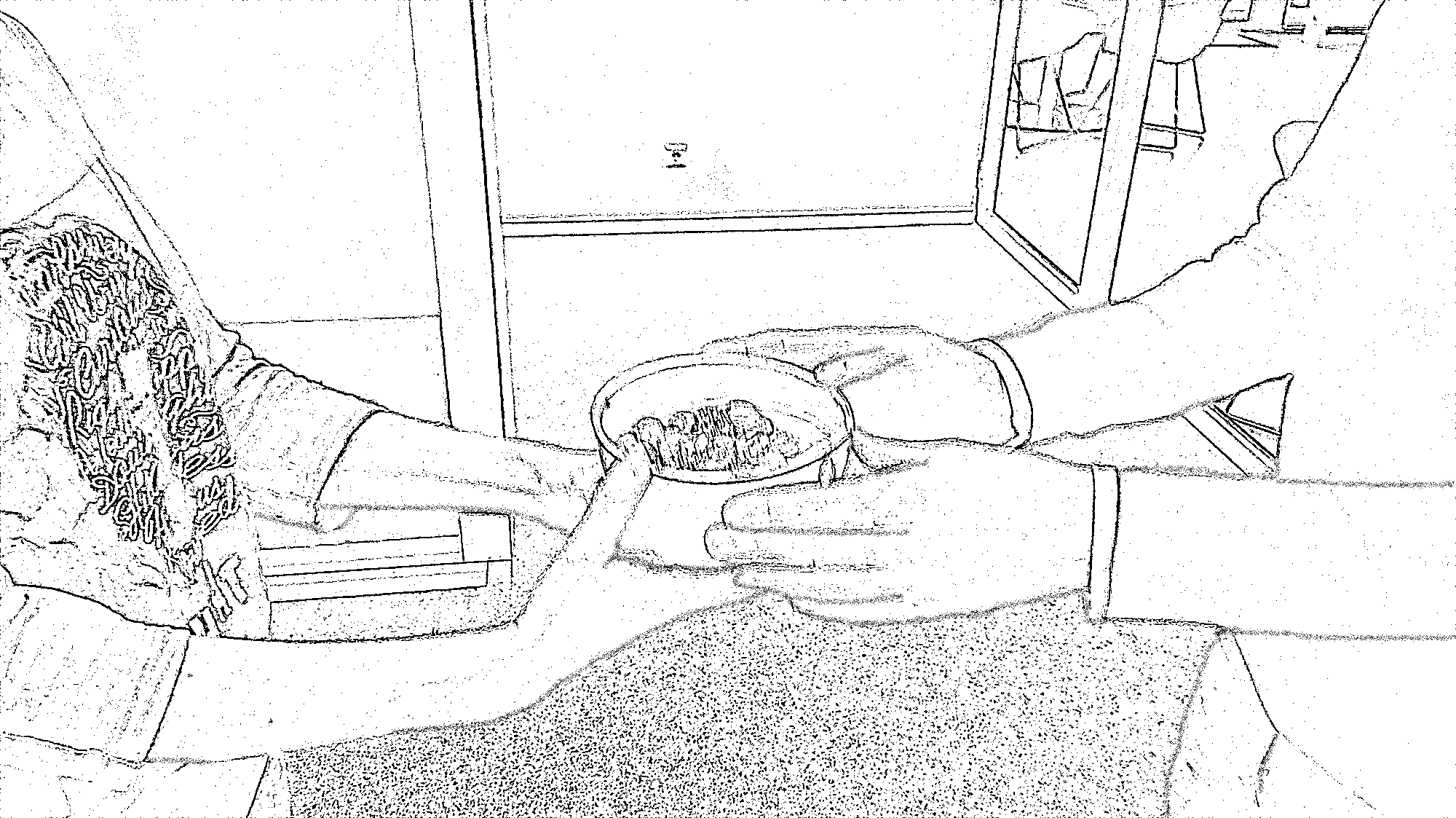

Supplement: Supplementary file 3 [file DataSheet3.ZIP › Matroesjka1_3rdperson.png]

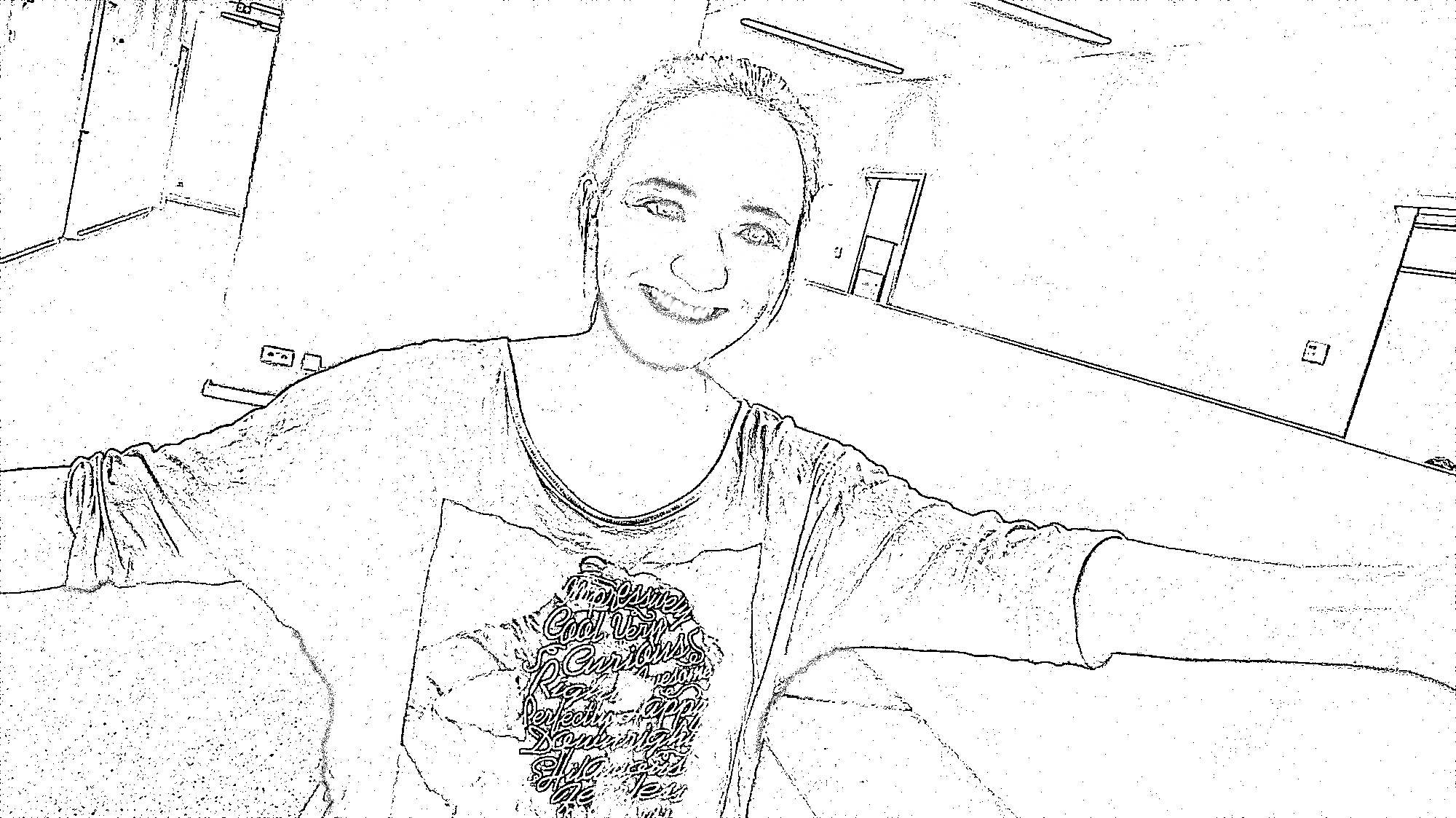

Supplement: Supplementary file 3 [file DataSheet3.ZIP › Matroesjka2_1stperson.png]

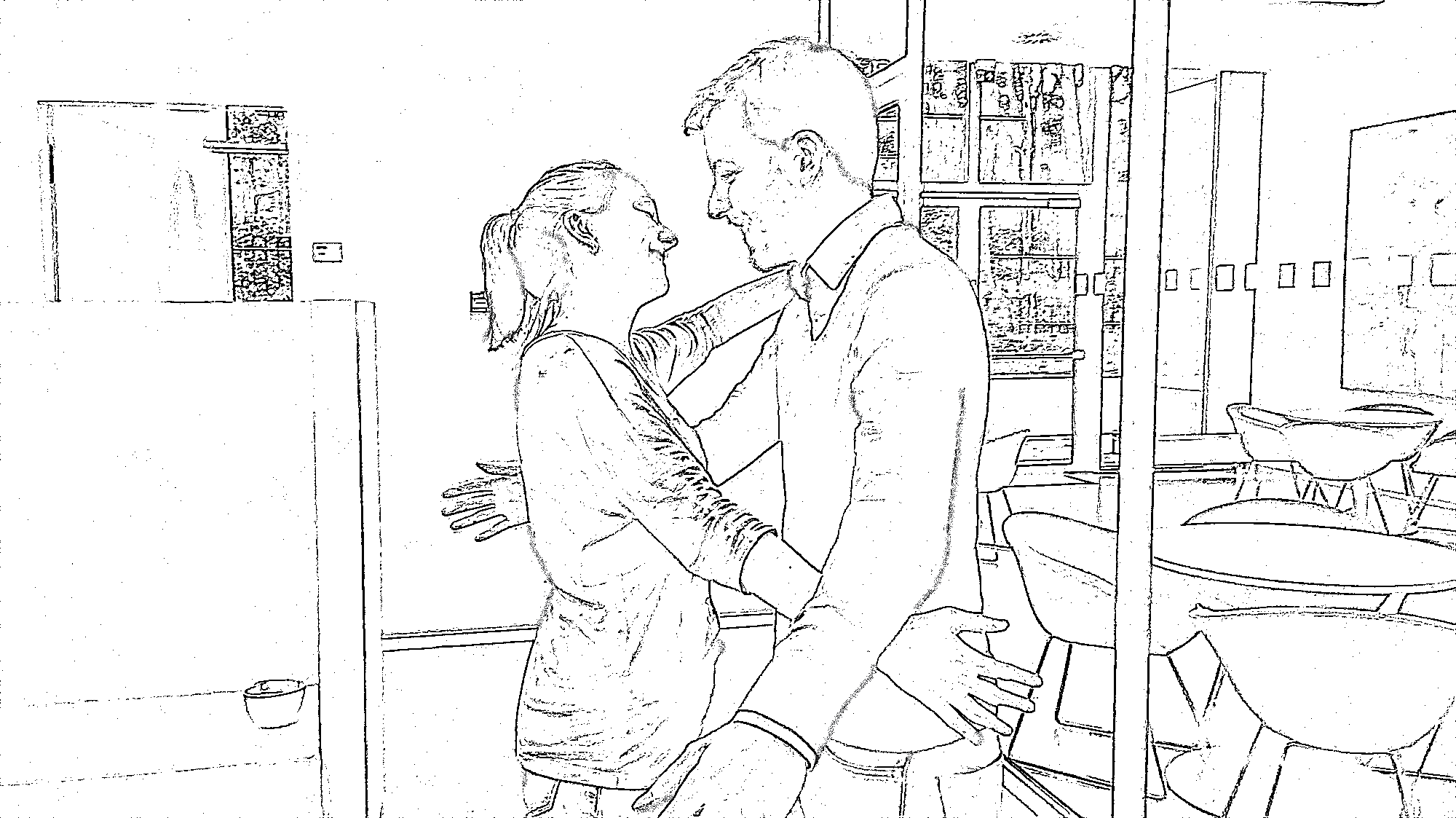

Supplement: Supplementary file 3 [file DataSheet3.ZIP › Matroesjka2_3rdperson.png]

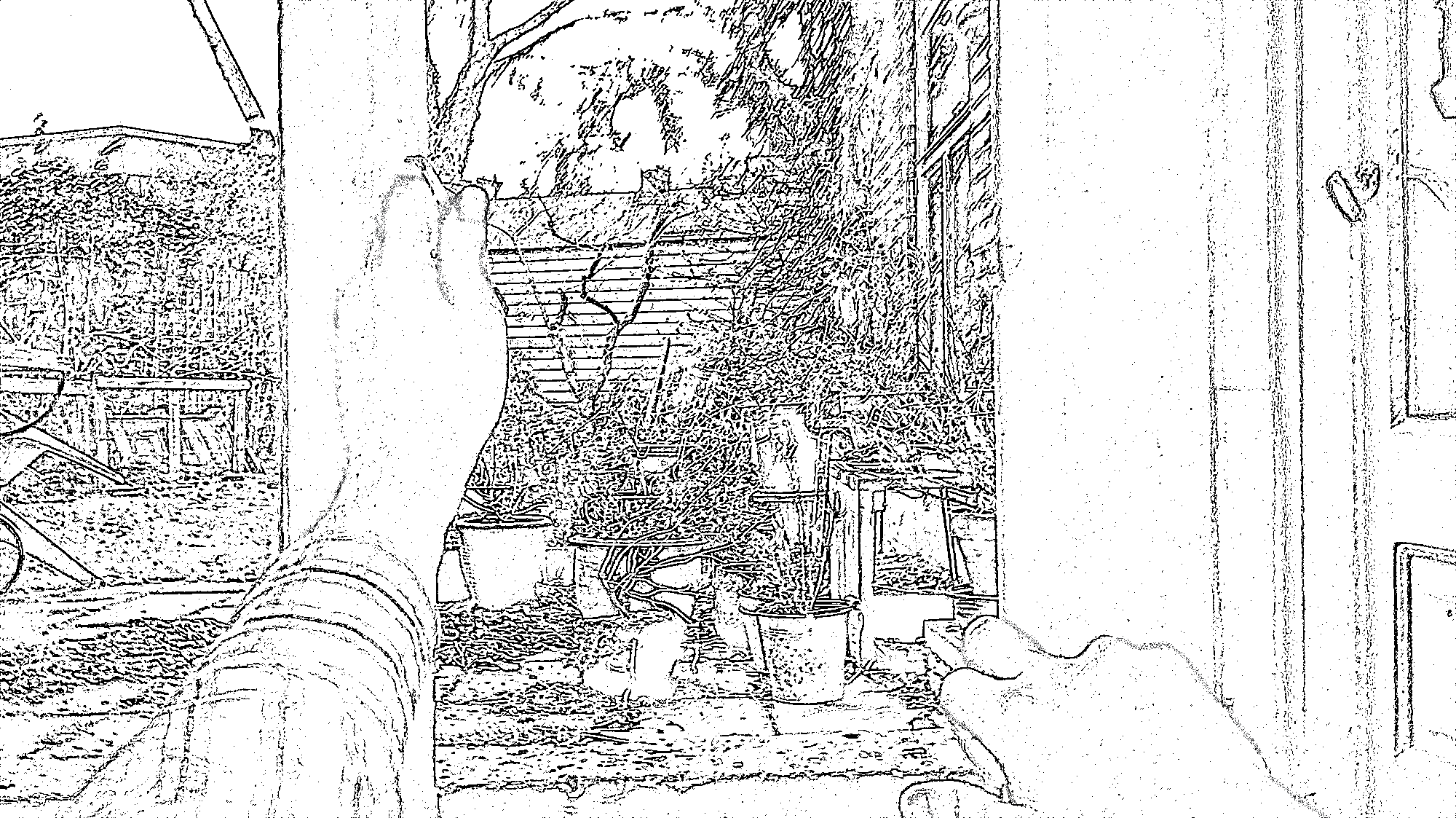

Supplement: Supplementary file 3 [file DataSheet3.ZIP › Meesterwerk1_1stperson.png]

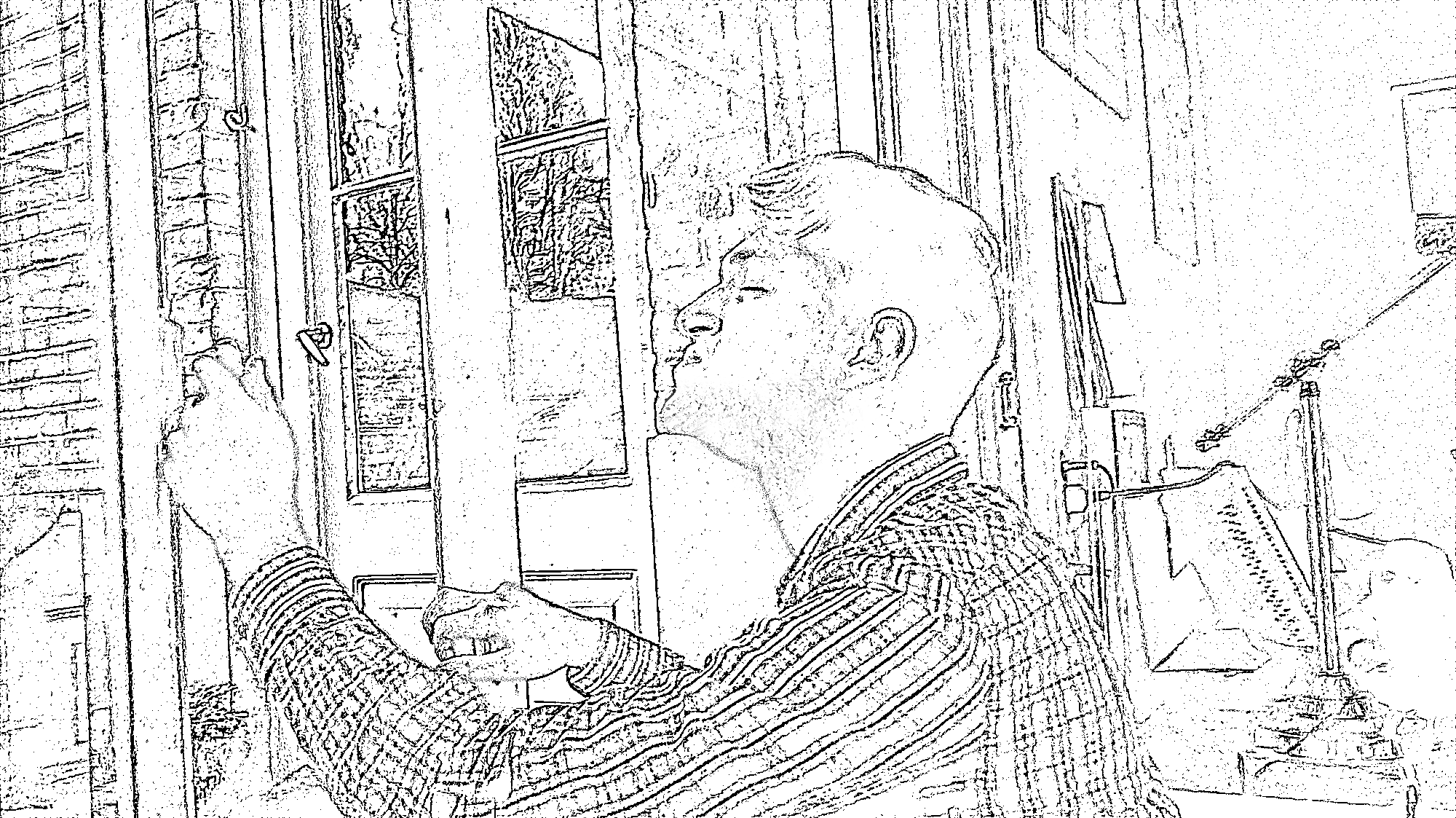

Supplement: Supplementary file 3 [file DataSheet3.ZIP › Meesterwerk1_3rdperson.png]

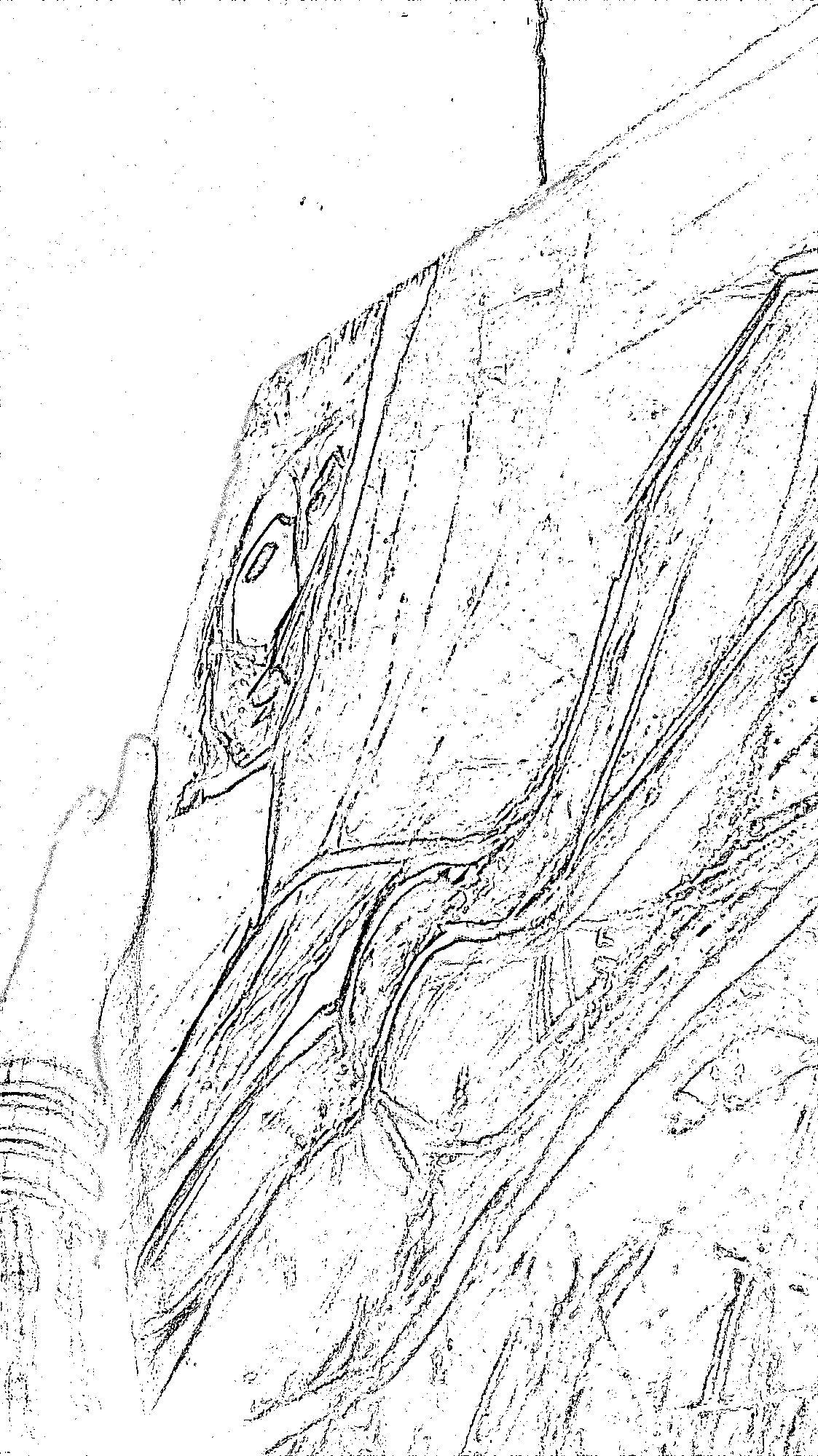

Supplement: Supplementary file 3 [file DataSheet3.ZIP › Meesterwerk2_1stperson.png]

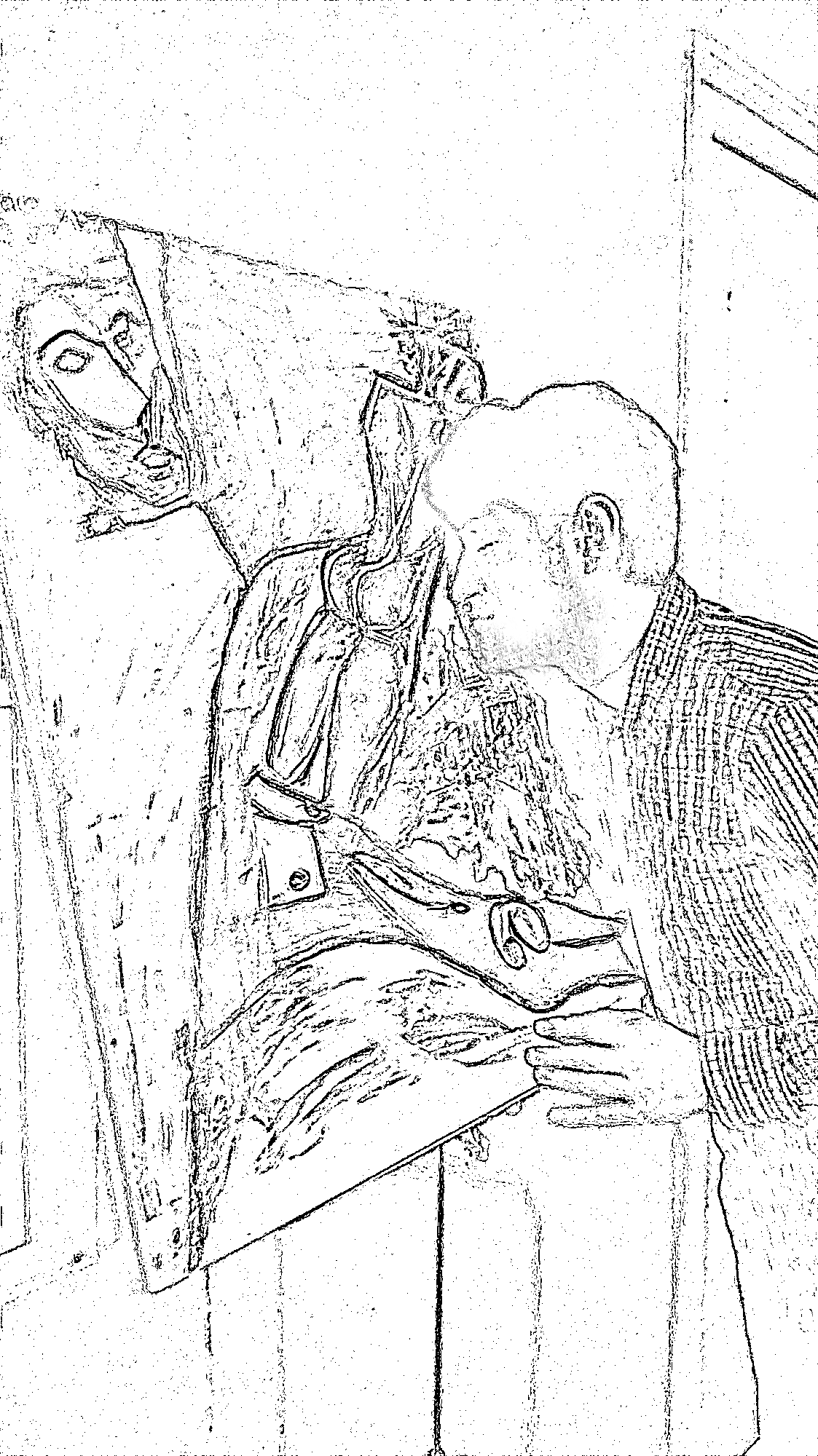

Supplement: Supplementary file 3 [file DataSheet3.ZIP › Meesterwerk2_3rdperson.png]
