# Supplementary material for: When Fiction Is Just as Real as Fact: No Differences in Reading Behavior between Stories Believed to be Based on True or Fictional Events
Source: Front Psychol. 2017 Sep 20;8:1618. doi: 10.3389/fpsyg.2017.01618 (PMC5613255; doi:10.3389/fpsyg.2017.01618)
Supplement: Supplementary file 4 [file DataSheet4.DOCX]

# S4: Statistical models

Contents

[Supplementary material 1: Statistical models 1](#_Toc456108715)

[Reading time 2](#_Toc456108716)

[Perspective taking 3](#_Toc456108717)

[Immersion questionnaire 5](#_Toc456108718)

[Appreciation measure 9](#_Toc456108719)

[Picture task 19](#_Toc456108720)

## Reading time

> fullmodelRT = lmer (ReadingTime ~ Perspective*Condition + P1 + P2 + Gender + Age + Education + Dutch + LikeFiction*Condition + LikeFact*Condition + (1|Story), data=odata, REML=TRUE)

> summary (fullmodelRT)

Linear mixed model fit by REML ['lmerMod']

Formula: ReadingTime ~ Perspective * Condition + P1 + P2 + Gender + Age +

Education + Dutch + LikeFiction * Condition + LikeFact * Condition + (1 | Story)

Data: odata

REML criterion at convergence: 21073.4

Scaled residuals:

Min 1Q Median 3Q Max

-3.0534 -0.4520 -0.1570 0.2217 12.6392

Random effects:

Groups Name Variance Std.Dev.

Story (Intercept) 2546 50.46

Residual 7466 86.40

Number of obs: 1796, groups: Story, 4

Fixed effects:

Estimate Std. Error t value

(Intercept) 200.3050 32.6842 6.129

Perspective3rd 9.1738 5.8509 1.568

ConditionFiction 2.5328 21.3943 0.118

P1 2.1431 1.4604 1.468

P2 2.3190 1.5172 1.528

Gender2 12.8101 4.7223 2.713

Age 0.2241 0.1272 1.762

Education -6.2683 3.3361 -1.879

DutchTRUE -28.5958 6.8145 -4.196

LikeFiction -3.3148 0.7145 -4.640

LikeFact 1.0655 0.8721 1.222

Perspective3rd:ConditionFiction -0.1443 8.2502 -0.017

ConditionFiction:LikeFiction 1.7761 0.9966 1.782

ConditionFiction:LikeFact -1.6406 1.2196 -1.345

> PValues_RT <- Anova(fullmodelRT, type = 3, test = 'F')

> PValues_RT

Analysis of Deviance Table (Type III Wald F tests with Kenward-Roger df)

Response: ReadingTime

F Df Df.res Pr(>F)

(Intercept) 37.5584 1 8.33 0.0002386 ***

Perspective 2.4583 1 1779.14 0.1170860

Condition 0.0140 1 1779.11 0.9057756

P1 2.1533 1 1779.34 0.1424392

P2 2.3359 1 1779.39 0.1265981

Gender 7.3584 1 1779.09 0.0067394 **

Age 3.1031 1 1779.02 0.0783140 .

Education 3.5304 1 1779.02 0.0604154 .

Dutch 17.6083 1 1779.12 2.848e-05 ***

LikeFiction 21.5258 1 1779.07 3.746e-06 ***

LikeFact 1.4926 1 1779.08 0.2219699

Perspective:Condition 0.0003 1 1779.35 0.9860452

Condition:LikeFiction 3.1757 1 1779.04 0.0749123 .

Condition:LikeFact 1.8095 1 1779.10 0.1787430

---

Signif. codes: 0 ‘***’ 0.001 ‘**’ 0.01 ‘*’ 0.05 ‘.’ 0.1 ‘ ’ 1

## Perspective taking

> fullmodelP1 = lmer (P1 ~ Perspective*Condition + Gender + Age + Education + Dutch +

LikeFiction*Condition + LikeFact*Condition + (1|Story), data=odata, REML=FALSE)

> summary (fullmodelP1)

Linear mixed model fit by maximum likelihood ['lmerMod']

Formula: P1 ~ Perspective * Condition + Gender + Age + Education + Dutch +

LikeFiction * Condition + LikeFact * Condition + (1 | Story)

Data: odata

AIC BIC logLik deviance df.resid

6872.6 6949.5 -3422.3 6844.6 1782

Scaled residuals:

Min 1Q Median 3Q Max

-2.25307 -0.75444 0.00836 0.76427 2.43829

Random effects:

Groups Name Variance Std.Dev.

Story (Intercept) 0.1185 0.3442

Residual 2.6283 1.6212

Number of obs: 1796, groups: Story, 4

Fixed effects:

Estimate Std. Error t value

(Intercept) 4.018699 0.406890 9.877

Perspective3rd -0.236968 0.109614 -2.162

ConditionFiction 0.364871 0.401262 0.909

Gender2 -0.264304 0.088212 -2.996

Age -0.009580 0.002375 -4.033

Education -0.039669 0.062552 -0.634

DutchTRUE -0.064242 0.127824 -0.503

LikeFiction 0.027227 0.013389 2.034

LikeFact 0.016266 0.016353 0.995

Perspective3rd:ConditionFiction 0.149042 0.154703 0.963

ConditionFiction:LikeFiction -0.028885 0.018684 -1.546

ConditionFiction:LikeFact 0.005346 0.022881 0.234

Correlation of Fixed Effects:

(Intr) Prspc3 CndtnF Gendr2 Age Eductn DtTRUE LkFctn LikFct Pr3:CF CndtnFctn:LkFctn

Perspctv3rd -0.125

ConditnFctn -0.472 0.106

Gender2 -0.110 -0.015 0.021

Age -0.240 0.033 0.026 -0.152

Education -0.440 0.003 -0.012 0.066 -0.113

DutchTRUE -0.235 0.047 -0.041 -0.011 -0.003 -0.006

LikeFiction -0.304 0.017 0.338 0.073 0.162 -0.149 -0.046

LikeFact -0.406 -0.065 0.477 0.042 -0.112 -0.050 -0.043 -0.230

Prspctv3:CF 0.094 -0.714 -0.193 0.017 -0.036 -0.009 -0.030 -0.010 0.044

CndtnFctn:LkFctn 0.219 -0.011 -0.489 0.004 -0.003 0.030 -0.004 -0.680 0.161 0.000

CondtnFctn:LkFct 0.335 0.042 -0.688 -0.027 -0.023 -0.006 0.059 0.153 -0.702 0.006 -0.238

>

> PValues_P1 <- Anova(fullmodelP1, type = 3, test = 'Chi')

> PValues_P1

Analysis of Deviance Table (Type III Wald chisquare tests)

Response: P1

Chisq Df Pr(>Chisq)

(Intercept) 97.5477 1 < 2.2e-16 ***

Perspective 4.6735 1 0.030631 *

Condition 0.8268 1 0.363187

Gender 8.9775 1 0.002733 **

Age 16.2662 1 5.504e-05 ***

Education 0.4022 1 0.525970

Dutch 0.2526 1 0.615260

LikeFiction 4.1352 1 0.042001 *

LikeFact 0.9894 1 0.319892

Perspective:Condition 0.9282 1 0.335342

Condition:LikeFiction 2.3901 1 0.122103

Condition:LikeFact 0.0546 1 0.815276

---

Signif. codes: 0 ‘***’ 0.001 ‘**’ 0.01 ‘*’ 0.05 ‘.’ 0.1 ‘ ’ 1

> fullmodelP2 = lmer (P2 ~ Perspective*Condition + Gender + Age + Education + Dutch +

LikeFiction*Condition + LikeFact*Condition + (1|Story), data=odata, REML=FALSE)

> summary (fullmodelP2)

Linear mixed model fit by maximum likelihood ['lmerMod']

Formula: P2 ~ Perspective * Condition + Gender + Age + Education + Dutch +

LikeFiction * Condition + LikeFact * Condition + (1 | Story)

Data: odata

AIC BIC logLik deviance df.resid

6735.7 6812.6 -3353.9 6707.7 1782

Scaled residuals:

Min 1Q Median 3Q Max

-2.7206 -0.6585 0.1321 0.7252 2.1508

Random effects:

Groups Name Variance Std.Dev.

Story (Intercept) 0.1155 0.3398

Residual 2.4352 1.5605

Number of obs: 1796, groups: Story, 4

Fixed effects:

Estimate Std. Error t value

(Intercept) 4.9086375 0.3934729 12.475

Perspective3rd -0.0612594 0.1055119 -0.581

ConditionFiction 0.0090534 0.3862436 0.023

Gender2 -0.3199946 0.0849102 -3.769

Age -0.0069184 0.0022863 -3.026

Education -0.0927683 0.0602105 -1.541

DutchTRUE -0.0978699 0.1230399 -0.795

LikeFiction 0.0155757 0.0128879 1.209

LikeFact 0.0217475 0.0157414 1.382

Perspective3rd:ConditionFiction 0.1212676 0.1489142 0.814

ConditionFiction:LikeFiction -0.0022513 0.0179844 -0.125

ConditionFiction:LikeFact -0.0004231 0.0220251 -0.019

Correlation of Fixed Effects:

(Intr) Prspc3 CndtnF Gendr2 Age Eductn DtTRUE LkFctn LikFct Pr3:CF CndtnFctn:LkFctn

Perspctv3rd -0.124

ConditnFctn -0.470 0.106

Gender2 -0.110 -0.015 0.021

Age -0.238 0.033 0.026 -0.152

Education -0.438 0.003 -0.012 0.066 -0.113

DutchTRUE -0.234 0.047 -0.041 -0.011 -0.003 -0.006

LikeFiction -0.302 0.017 0.338 0.073 0.162 -0.149 -0.046

LikeFact -0.404 -0.065 0.477 0.042 -0.112 -0.050 -0.043 -0.230

Prspctv3:CF 0.094 -0.714 -0.193 0.017 -0.036 -0.009 -0.030 -0.010 0.044

CndtnFctn:LkFctn 0.218 -0.011 -0.489 0.004 -0.003 0.030 -0.004 -0.680 0.161 0.000

CondtnFctn:LkFct 0.334 0.042 -0.688 -0.027 -0.023 -0.006 0.059 0.153 -0.702 0.006 -0.238

>

> PValues_P2 <- Anova(fullmodelP2, type = 3, test = 'Chi')

> PValues_P2

Analysis of Deviance Table (Type III Wald chisquare tests)

Response: P2

Chisq Df Pr(>Chisq)

(Intercept) 155.6296 1 < 2.2e-16 ***

Perspective 0.3371 1 0.5615148

Condition 0.0005 1 0.9812996

Gender 14.2025 1 0.0001642 ***

Age 9.1567 1 0.0024781 **

Education 2.3739 1 0.1233819

Dutch 0.6327 1 0.4263618

LikeFiction 1.4606 1 0.2268355

LikeFact 1.9087 1 0.1671105

Perspective:Condition 0.6632 1 0.4154470

Condition:LikeFiction 0.0157 1 0.9003810

Condition:LikeFact 0.0004 1 0.9846752

---

Signif. codes: 0 ‘***’ 0.001 ‘**’ 0.01 ‘*’ 0.05 ‘.’ 0.1 ‘ ’ 1

## Immersion questionnaire

> fullmodelATT = lmer (Attention ~ Perspective*Condition + P1 + P2 + Gender + Age + Education + Dutch + LikeFiction*Condition + LikeFact*Condition + (1|Story), data=odata, REML=FALSE)

> summary (fullmodelATT)

Linear mixed model fit by maximum likelihood ['lmerMod']

Formula: Attention ~ Perspective * Condition + P1 + P2 + Gender + Age +

Education + Dutch + LikeFiction * Condition + LikeFact * Condition + (1 | Story)

Data: odata

AIC BIC logLik deviance df.resid

5350.2 5438.1 -2659.1 5318.2 1780

Scaled residuals:

Min 1Q Median 3Q Max

-3.9910 -0.6967 0.0159 0.6488 4.2452

Random effects:

Groups Name Variance Std.Dev.

Story (Intercept) 0.0001547 0.01244

Residual 1.1310361 1.06350

Number of obs: 1796, groups: Story, 4

Fixed effects:

Estimate Std. Error t value

(Intercept) 1.658428 0.255609 6.488

Perspective3rd -0.121461 0.071817 -1.691

ConditionFiction 0.035586 0.262752 0.135

P1 0.355591 0.017860 19.910

P2 0.291034 0.018538 15.700

Gender2 -0.011080 0.058024 -0.191

Age -0.002378 0.001565 -1.519

Education -0.044703 0.041044 -1.089

DutchTRUE -0.024266 0.083676 -0.290

LikeFiction -0.015299 0.008782 -1.742

LikeFact 0.011319 0.010717 1.056

Perspective3rd:ConditionFiction 0.132857 0.100830 1.318

ConditionFiction:LikeFiction 0.006146 0.012258 0.501

ConditionFiction:LikeFact -0.013188 0.014982 -0.880

>

> PValues_ATT <- Anova(fullmodelATT, type = 3, test = 'Chi')

> PValues_ATT

Analysis of Deviance Table (Type III Wald chisquare tests)

Response: Attention

Chisq Df Pr(>Chisq)

(Intercept) 42.0961 1 8.69e-11 ***

Perspective 2.8603 1 0.09079 .

Condition 0.0183 1 0.89227

P1 396.3916 1 < 2.2e-16 ***

P2 246.4784 1 < 2.2e-16 ***

Gender 0.0365 1 0.84857

Age 2.3081 1 0.12870

Education 1.1862 1 0.27609

Dutch 0.0841 1 0.77182

LikeFiction 3.0346 1 0.08150 .

LikeFact 1.1155 1 0.29088

Perspective:Condition 1.7362 1 0.18762

Condition:LikeFiction 0.2514 1 0.61608

Condition:LikeFact 0.7749 1 0.37872

---

Signif. codes: 0 ‘***’ 0.001 ‘**’ 0.01 ‘*’ 0.05 ‘.’ 0.1 ‘ ’ 1

| > fullmodelTRA = lmer (Transportation ~ Perspective*Condition + P1 + P2 + Gender + Age +  Education + Dutch + LikeFiction*Condition + LikeFact*Condition + (1\|Story), data=odata, REML=FALSE)  > summary (fullmodelTRA)  Linear mixed model fit by maximum likelihood ['lmerMod']  Formula: Transportation ~ Perspective * Condition + P1 + P2 + Gender +  Age + Education + Dutch + LikeFiction * Condition + LikeFact * Condition + (1 \| Story)  Data: odata  AIC BIC logLik deviance df.resid  4867.3 4955.1 -2417.6 4835.3 1780  Scaled residuals:  Min 1Q Median 3Q Max  -3.4224 -0.6123 -0.0211 0.6455 3.8222  Random effects:  Groups Name Variance Std.Dev.  Story (Intercept) 0.004976 0.07054  Residual 0.862008 0.92844  Number of obs: 1796, groups: Story, 4  Fixed effects:  Estimate Std. Error t value  (Intercept) 1.058719 0.225966 4.685  Perspective3rd -0.014964 0.062819 -0.238  ConditionFiction 0.092955 0.229739 0.405  P1 0.420756 0.015661 26.866  P2 0.268723 0.016265 16.521  Gender2 0.016834 0.050718 0.332  Age -0.005678 0.001367 -4.154  Education -0.022948 0.035843 -0.640  DutchTRUE -0.119167 0.073172 -1.629  LikeFiction -0.009652 0.007674 -1.258  LikeFact 0.010472 0.009367 1.118  Perspective3rd:ConditionFiction 0.030290 0.088464 0.342  ConditionFiction:LikeFiction 0.002888 0.010707 0.270  ConditionFiction:LikeFact -0.010395 0.013098 -0.794  >  > PValues_TRA <- Anova(fullmodelTRA, type = 3, test = 'Chi')  > PValues_TRA  Analysis of Deviance Table (Type III Wald chisquare tests)  Response: Transportation  Chisq Df Pr(>Chisq)  (Intercept) 21.9522 1 2.795e-06 ***  Perspective 0.0567 1 0.8117  Condition 0.1637 1 0.6858  P1 721.8010 1 < 2.2e-16 ***  P2 272.9599 1 < 2.2e-16 ***  Gender 0.1102 1 0.7399  Age 17.2573 1 3.264e-05 ***  Education 0.4099 1 0.5220  Dutch 2.6523 1 0.1034  LikeFiction 1.5820 1 0.2085  LikeFact 1.2499 1 0.2636  Perspective:Condition 0.1172 1 0.7321  Condition:LikeFiction 0.0727 1 0.7874  Condition:LikeFact 0.6299 1 0.4274  ---  Signif. codes: 0 ‘***’ 0.001 ‘**’ 0.01 ‘*’ 0.05 ‘.’ 0.1 ‘ ’ 1  > fullmodelEMO = lmer (EmotionalEngagement ~ Perspective*Condition + P1 + P2 + Gender + Age +  Education + Dutch + LikeFiction*Condition + LikeFact*Condition + (1\|Story), data=odata, REML=FALSE)  > summary (fullmodelEMO)  Linear mixed model fit by maximum likelihood ['lmerMod']  Formula: EmotionalEngagement ~ Perspective * Condition + P1 + P2 + Gender +  Age + Education + Dutch + LikeFiction * Condition + LikeFact * Condition + (1 \| Story)  Data: odata  AIC BIC logLik deviance df.resid  4955.3 5043.2 -2461.7 4923.3 1780  Scaled residuals:  Min 1Q Median 3Q Max  -3.9680 -0.6486 0.0028 0.6618 3.7911  Random effects:  Groups Name Variance Std.Dev.  Story (Intercept) 0.01497 0.1223  Residual 0.90360 0.9506  Number of obs: 1796, groups: Story, 4  Fixed effects:  Estimate Std. Error t value  (Intercept) 1.204e+00 2.366e-01 5.090  Perspective3rd -1.348e-01 6.435e-02 -2.094  ConditionFiction 3.158e-02 2.353e-01 0.134  P1 4.641e-01 1.605e-02 28.908  P2 2.483e-01 1.668e-02 14.892  Gender2 8.140e-03 5.194e-02 0.157  Age -4.112e-03 1.399e-03 -2.939  Education 3.023e-02 3.670e-02 0.824  DutchTRUE -5.133e-02 7.495e-02 -0.685  LikeFiction -1.381e-02 7.859e-03 -1.757  LikeFact 5.399e-03 9.593e-03 0.563  Perspective3rd:ConditionFiction 8.369e-02 9.069e-02 0.923  ConditionFiction:LikeFiction -6.539e-05 1.096e-02 -0.006  ConditionFiction:LikeFact -1.616e-03 1.341e-02 -0.120  >  > PValues_EMO <- Anova(fullmodelEMO, type = 3, test = 'Chi')  > PValues_EMO  Analysis of Deviance Table (Type III Wald chisquare tests)  Response: EmotionalEngagement  Chisq Df Pr(>Chisq)  (Intercept) 25.9047 1 3.587e-07 ***  Perspective 4.3860 1 0.036236 *  Condition 0.0180 1 0.893252  P1 835.6737 1 < 2.2e-16 ***  P2 221.7786 1 < 2.2e-16 ***  Gender 0.0246 1 0.875479  Age 8.6358 1 0.003296 **  Education 0.6784 1 0.410135  Dutch 0.4690 1 0.493430  LikeFiction 3.0863 1 0.078956 .  LikeFact 0.3167 1 0.573580  Perspective:Condition 0.8517 1 0.356077  Condition:LikeFiction 0.0000 1 0.995242  Condition:LikeFact 0.0145 1 0.904102  ---  Signif. codes: 0 ‘***’ 0.001 ‘**’ 0.01 ‘*’ 0.05 ‘.’ 0.1 ‘ ’ 1  > fullmodelIMA = lmer (MentalImagery ~ Perspective*Condition + P1 + P2 + Gender + Age +  Education + Dutch + LikeFiction*Condition + LikeFact*Condition + (1\|Story), data=odata, REML=FALSE)  > summary (fullmodelIMA)  Linear mixed model fit by maximum likelihood ['lmerMod']  Formula: MentalImagery ~ Perspective * Condition + P1 + P2 + Gender +  Age + Education + Dutch + LikeFiction * Condition + LikeFact * Condition + (1 \| Story)  Data: odata  AIC BIC logLik deviance df.resid  4819.1 4907.0 -2393.6 4787.1 1780  Scaled residuals:  Min 1Q Median 3Q Max  -3.4443 -0.6631 0.0440 0.6115 3.9070  Random effects:  Groups Name Variance Std.Dev.  Story (Intercept) 0.0006234 0.02497  Residual 0.8410820 0.91711  Number of obs: 1796, groups: Story, 4  Fixed effects:  Estimate Std. Error t value  (Intercept) 1.859795 0.220742 8.425  Perspective3rd 0.023162 0.061965 0.374  ConditionFiction 0.105179 0.226682 0.464  P1 0.297065 0.015420 19.264  P2 0.383391 0.016008 23.950  Gender2 -0.165178 0.050054 -3.300  Age -0.004570 0.001350 -3.386  Education -0.034838 0.035397 -0.984  DutchTRUE 0.038908 0.072192 0.539  LikeFiction -0.001228 0.007575 -0.162  LikeFact 0.013374 0.009245 1.447  Perspective3rd:ConditionFiction -0.065576 0.087073 -0.753  ConditionFiction:LikeFiction 0.008683 0.010572 0.821  ConditionFiction:LikeFact -0.010360 0.012925 -0.802  >  > PValues_IMA <- Anova(fullmodelIMA, type = 3, test = 'Chi')  > PValues_IMA  Analysis of Deviance Table (Type III Wald chisquare tests)  Response: MentalImagery  Chisq Df Pr(>Chisq)  (Intercept) 70.9837 1 < 2.2e-16 ***  Perspective 0.1397 1 0.7085652  Condition 0.2153 1 0.6426532  P1 371.1147 1 < 2.2e-16 ***  P2 573.6130 1 < 2.2e-16 ***  Gender 10.8899 1 0.0009669 ***  Age 11.4617 1 0.0007105 ***  Education 0.9687 1 0.3250164  Dutch 0.2905 1 0.5899141  LikeFiction 0.0263 1 0.8712337  LikeFact 2.0928 1 0.1479953  Perspective:Condition 0.5672 1 0.4513759  Condition:LikeFiction 0.6745 1 0.4114881  Condition:LikeFact 0.6426 1 0.4227880  ---  Signif. codes: 0 ‘***’ 0.001 ‘**’ 0.01 ‘*’ 0.05 ‘.’ 0.1 ‘ ’ 1 |
| --- |

## Appreciation measure

> fullmodelinteresting = lmer (interesting ~ Perspective*Condition + P1 + P2 + Gender + Age +

Education + Dutch + LikeFiction*Condition + LikeFact*Condition + (1|Story), data=appdata, REML=FALSE)

> summary (fullmodelinteresting)

Linear mixed model fit by maximum likelihood ['lmerMod']

Formula: interesting ~ Perspective * Condition + P1 + P2 + Gender + Age +

Education + Dutch + LikeFiction * Condition + LikeFact * Condition + (1 | Story)

Data: appdata

AIC BIC logLik deviance df.resid

6042.3 6129.8 -3005.1 6010.3 1745

Scaled residuals:

Min 1Q Median 3Q Max

-3.6457 -0.7005 0.0480 0.7310 3.0090

Random effects:

Groups Name Variance Std.Dev.

Story (Intercept) 0.03144 0.1773

Residual 1.76856 1.3299

Number of obs: 1761, groups: Story, 4

Fixed effects:

Estimate Std. Error t value

(Intercept) 1.635963 0.316542 5.168

Perspective3rd -0.133663 0.090777 -1.472

ConditionFiction -0.225504 0.270875 -0.833

P1 0.321662 0.022694 14.174

P2 0.276133 0.023740 11.632

Gender2 -0.021931 0.073179 -0.300

Age -0.003797 0.001980 -1.918

Education 0.062535 0.051466 1.215

DutchTRUE -0.105460 0.106780 -0.988

LikeFiction -0.004765 0.010513 -0.453

LikeFact 0.005274 0.012171 0.433

Perspective3rd:ConditionFiction 0.020167 0.128049 0.157

ConditionFiction:LikeFiction 0.018406 0.014586 1.262

ConditionFiction:LikeFact -0.004677 0.016857 -0.277

>

> PValues_interesting <- Anova(fullmodelinteresting, type = 3, test = 'Chi')

> PValues_interesting

Analysis of Deviance Table (Type III Wald chisquare tests)

Response: interesting

Chisq Df Pr(>Chisq)

(Intercept) 26.7106 1 2.363e-07 ***

Perspective 2.1681 1 0.14090

Condition 0.6931 1 0.40512

P1 200.9047 1 < 2.2e-16 ***

P2 135.2975 1 < 2.2e-16 ***

Gender 0.0898 1 0.76442

Age 3.6778 1 0.05514 .

Education 1.4764 1 0.22434

Dutch 0.9754 1 0.32333

LikeFiction 0.2054 1 0.65039

LikeFact 0.1877 1 0.66480

Perspective:Condition 0.0248 1 0.87486

Condition:LikeFiction 1.5923 1 0.20699

Condition:LikeFact 0.0770 1 0.78146

---

Signif. codes: 0 ‘***’ 0.001 ‘**’ 0.01 ‘*’ 0.05 ‘.’ 0.1 ‘ ’ 1

> fullmodelwellwritten = lmer (wellwritten ~ Perspective*Condition + P1 + P2 + Gender + Age +

Education + Dutch + LikeFiction*Condition + LikeFact*Condition + (1|Story), data=appdata, REML=FALSE)

> summary (fullmodelwellwritten)

Linear mixed model fit by maximum likelihood ['lmerMod']

Formula: wellwritten ~ Perspective * Condition + P1 + P2 + Gender + Age +

Education + Dutch + LikeFiction * Condition + LikeFact * Condition + (1 | Story)

Data: appdata

AIC BIC logLik deviance df.resid

6255.0 6342.6 -3111.5 6223.0 1745

Scaled residuals:

Min 1Q Median 3Q Max

-3.5270 -0.7143 0.0517 0.7190 3.4920

Random effects:

Groups Name Variance Std.Dev.

Story (Intercept) 0.02047 0.1431

Residual 1.99778 1.4134

Number of obs: 1761, groups: Story, 4

Fixed effects:

Estimate Std. Error t value

(Intercept) 2.181391 0.330773 6.595

Perspective3rd -0.156617 0.096461 -1.624

ConditionFiction -0.196162 0.287874 -0.681

P1 0.303226 0.024106 12.579

P2 0.302750 0.025217 12.006

Gender2 -0.037282 0.077768 -0.479

Age -0.002939 0.002104 -1.397

Education -0.014294 0.054698 -0.261

DutchTRUE -0.117442 0.113468 -1.035

LikeFiction -0.024671 0.011173 -2.208

LikeFact 0.009735 0.012934 0.753

Perspective3rd:ConditionFiction -0.097103 0.136021 -0.714

ConditionFiction:LikeFiction 0.027149 0.015501 1.751

ConditionFiction:LikeFact -0.011813 0.017915 -0.659

>

> PValues_wellwritten <- Anova(fullmodelwellwritten, type = 3, test = 'Chi')

> PValues_wellwritten

Analysis of Deviance Table (Type III Wald chisquare tests)

Response: wellwritten

Chisq Df Pr(>Chisq)

(Intercept) 43.4917 1 4.258e-11 ***

Perspective 2.6362 1 0.10445

Condition 0.4643 1 0.49561

P1 158.2313 1 < 2.2e-16 ***

P2 144.1336 1 < 2.2e-16 ***

Gender 0.2298 1 0.63166

Age 1.9517 1 0.16241

Education 0.0683 1 0.79385

Dutch 1.0713 1 0.30066

LikeFiction 4.8755 1 0.02724 *

LikeFact 0.5665 1 0.45165

Perspective:Condition 0.5096 1 0.47530

Condition:LikeFiction 3.0674 1 0.07988 .

Condition:LikeFact 0.4348 1 0.50965

---

Signif. codes: 0 ‘***’ 0.001 ‘**’ 0.01 ‘*’ 0.05 ‘.’ 0.1 ‘ ’ 1

> fullmodelliterary = lmer (literary ~ Perspective*Condition + P1 + P2 + Gender + Age + Education +

Dutch + LikeFiction*Condition + LikeFact*Condition + (1|Story), data=appdata, REML=FALSE)

> summary (fullmodelliterary)

Linear mixed model fit by maximum likelihood ['lmerMod']

Formula: literary ~ Perspective * Condition + P1 + P2 + Gender + Age +

Education + Dutch + LikeFiction * Condition + LikeFact * Condition + (1 | Story)

Data: appdata

AIC BIC logLik deviance df.resid

5959.1 6046.7 -2963.6 5927.1 1745

Scaled residuals:

Min 1Q Median 3Q Max

-2.8358 -0.6962 0.0039 0.7029 4.1423

Random effects:

Groups Name Variance Std.Dev.

Story (Intercept) 0.06503 0.255

Residual 1.68426 1.298

Number of obs: 1761, groups: Story, 4

Fixed effects:

Estimate Std. Error t value

(Intercept) 1.436256 0.322807 4.449

Perspective3rd 0.034164 0.088602 0.386

ConditionFiction 0.031592 0.264356 0.120

P1 0.259304 0.022157 11.703

P2 0.261532 0.023178 11.284

Gender2 0.043770 0.071421 0.613

Age -0.005578 0.001932 -2.887

Education 0.058374 0.050226 1.162

DutchTRUE -0.046590 0.104220 -0.447

LikeFiction -0.025044 0.010261 -2.441

LikeFact 0.017642 0.011879 1.485

Perspective3rd:ConditionFiction -0.215929 0.125018 -1.727

ConditionFiction:LikeFiction 0.033860 0.014236 2.378

ConditionFiction:LikeFact -0.028562 0.016452 -1.736

>

> PValues_literary <- Anova(fullmodelliterary, type = 3, test = 'Chi')

> PValues_literary

Analysis of Deviance Table (Type III Wald chisquare tests)

Response: literary

Chisq Df Pr(>Chisq)

(Intercept) 19.7960 1 8.616e-06 ***

Perspective 0.1487 1 0.699802

Condition 0.0143 1 0.904874

P1 136.9580 1 < 2.2e-16 ***

P2 127.3221 1 < 2.2e-16 ***

Gender 0.3756 1 0.539980

Age 8.3358 1 0.003887 **

Education 1.3507 1 0.245147

Dutch 0.1998 1 0.654847

LikeFiction 5.9577 1 0.014653 *

LikeFact 2.2057 1 0.137498

Perspective:Condition 2.9832 1 0.084135 .

Condition:LikeFiction 5.6572 1 0.017384 *

Condition:LikeFact 3.0140 1 0.082550 .

---

Signif. codes: 0 ‘***’ 0.001 ‘**’ 0.01 ‘*’ 0.05 ‘.’ 0.1 ‘ ’ 1

> fullmodeleasytounderstand = lmer (easytounderstand ~ Perspective*Condition + P1 + P2 + Gender +

Age + Education + Dutch + LikeFiction*Condition + LikeFact*Condition + (1|Story), data=appdata, REML=FALSE)

> summary (fullmodeleasytounderstand)

Linear mixed model fit by maximum likelihood ['lmerMod']

Formula: easytounderstand ~ Perspective * Condition + P1 + P2 + Gender +

Age + Education + Dutch + LikeFiction * Condition + LikeFact * Condition + (1 | Story)

Data: appdata

AIC BIC logLik deviance df.resid

6550.4 6638.0 -3259.2 6518.4 1745

Scaled residuals:

Min 1Q Median 3Q Max

-3.0420 -0.7187 0.1181 0.7430 2.9303

Random effects:

Groups Name Variance Std.Dev.

Story (Intercept) 0.146 0.3821

Residual 2.354 1.5342

Number of obs: 1761, groups: Story, 4

Fixed effects:

Estimate Std. Error t value

(Intercept) 3.548271 0.399280 8.887

Perspective3rd -0.142510 0.104751 -1.360

ConditionFiction -0.580225 0.312528 -1.857

P1 0.179857 0.026199 6.865

P2 0.140608 0.027405 5.131

Gender2 -0.200035 0.084436 -2.369

Age 0.010976 0.002284 4.805

Education -0.166411 0.059378 -2.803

DutchTRUE -0.066842 0.123215 -0.542

LikeFiction 0.014821 0.012130 1.222

LikeFact 0.003231 0.014044 0.230

Perspective3rd:ConditionFiction -0.187622 0.147820 -1.269

ConditionFiction:LikeFiction 0.022480 0.016830 1.336

ConditionFiction:LikeFact 0.017273 0.019450 0.888

>

> PValues_easytounderstand <- Anova(fullmodeleasytounderstand, type = 3, test = 'Chi')

> PValues_easytounderstand

Analysis of Deviance Table (Type III Wald chisquare tests)

Response: easytounderstand

Chisq Df Pr(>Chisq)

(Intercept) 78.9729 1 < 2.2e-16 ***

Perspective 1.8508 1 0.17369

Condition 3.4468 1 0.06337 .

P1 47.1294 1 6.645e-12 ***

P2 26.3238 1 2.887e-07 ***

Gender 5.6125 1 0.01783 *

Age 23.0886 1 1.547e-06 ***

Education 7.8544 1 0.00507 **

Dutch 0.2943 1 0.58749

LikeFiction 1.4928 1 0.22178

LikeFact 0.0529 1 0.81803

Perspective:Condition 1.6110 1 0.20435

Condition:LikeFiction 1.7840 1 0.18165

Condition:LikeFact 0.7887 1 0.37449

---

Signif. codes: 0 ‘***’ 0.001 ‘**’ 0.01 ‘*’ 0.05 ‘.’ 0.1 ‘ ’ 1

> fullmodelaccessible = lmer (accessible ~ Perspective*Condition + P1 + P2 + Gender + Age + Education

+ Dutch + LikeFiction*Condition + LikeFact*Condition + (1|Story), data=appdata, REML=FALSE)

> summary (fullmodelaccessible)

Linear mixed model fit by maximum likelihood ['lmerMod']

Formula: accessible ~ Perspective * Condition + P1 + P2 + Gender + Age +

Education + Dutch + LikeFiction * Condition + LikeFact * Condition + (1 | Story)

Data: appdata

AIC BIC logLik deviance df.resid

6292.4 6380.0 -3130.2 6260.4 1745

Scaled residuals:

Min 1Q Median 3Q Max

-3.3882 -0.7023 0.0631 0.7038 2.8125

Random effects:

Groups Name Variance Std.Dev.

Story (Intercept) 0.08672 0.2945

Residual 2.03475 1.4264

Number of obs: 1761, groups: Story, 4

Fixed effects:

Estimate Std. Error t value

(Intercept) 2.642548 0.357673 7.388

Perspective3rd -0.096266 0.097387 -0.988

ConditionFiction -0.277276 0.290565 -0.954

P1 0.223828 0.024355 9.190

P2 0.216163 0.025477 8.485

Gender2 -0.095381 0.078502 -1.215

Age 0.005031 0.002124 2.369

Education -0.080298 0.055206 -1.455

DutchTRUE 0.031408 0.114553 0.274

LikeFiction 0.002605 0.011278 0.231

LikeFact 0.002194 0.013057 0.168

Perspective3rd:ConditionFiction -0.088898 0.137417 -0.647

ConditionFiction:LikeFiction 0.013211 0.015647 0.844

ConditionFiction:LikeFact 0.009180 0.018083 0.508

>

> PValues_accessible <- Anova(fullmodelaccessible, type = 3, test = 'Chi')

> PValues_accessible

Analysis of Deviance Table (Type III Wald chisquare tests)

Response: accessible

Chisq Df Pr(>Chisq)

(Intercept) 54.5849 1 1.489e-13 ***

Perspective 0.9771 1 0.32291

Condition 0.9106 1 0.33995

P1 84.4611 1 < 2.2e-16 ***

P2 71.9910 1 < 2.2e-16 ***

Gender 1.4763 1 0.22436

Age 5.6118 1 0.01784 *

Education 2.1157 1 0.14580

Dutch 0.0752 1 0.78394

LikeFiction 0.0534 1 0.81730

LikeFact 0.0282 1 0.86655

Perspective:Condition 0.4185 1 0.51768

Condition:LikeFiction 0.7128 1 0.39852

Condition:LikeFact 0.2577 1 0.61168

---

Signif. codes: 0 ‘***’ 0.001 ‘**’ 0.01 ‘*’ 0.05 ‘.’ 0.1 ‘ ’ 1

> fullmodelthrilling = lmer (thrilling ~ Perspective*Condition + P1 + P2 + Gender + Age + Education +

Dutch + LikeFiction*Condition + LikeFact*Condition + (1|Story), data=appdata, REML=FALSE)

> summary (fullmodelthrilling)

Linear mixed model fit by maximum likelihood ['lmerMod']

Formula: thrilling ~ Perspective * Condition + P1 + P2 + Gender + Age +

Education + Dutch + LikeFiction * Condition + LikeFact * Condition + (1 | Story)

Data: appdata

AIC BIC logLik deviance df.resid

6003.0 6090.6 -2985.5 5971.0 1745

Scaled residuals:

Min 1Q Median 3Q Max

-2.7800 -0.7056 -0.0694 0.6898 3.0866

Random effects:

Groups Name Variance Std.Dev.

Story (Intercept) 0.0293 0.1712

Residual 1.7297 1.3152

Number of obs: 1761, groups: Story, 4

Fixed effects:

Estimate Std. Error t value

(Intercept) 0.897386 0.312469 2.872

Perspective3rd -0.082347 0.089774 -0.917

ConditionFiction 0.156778 0.267885 0.585

P1 0.266465 0.022442 11.873

P2 0.223699 0.023477 9.528

Gender2 -0.049711 0.072371 -0.687

Age 0.008955 0.001958 4.573

Education 0.012261 0.050898 0.241

DutchTRUE -0.242789 0.105600 -2.299

LikeFiction -0.017339 0.010397 -1.668

LikeFact 0.012456 0.012037 1.035

Perspective3rd:ConditionFiction -0.068840 0.126632 -0.544

ConditionFiction:LikeFiction 0.010385 0.014425 0.720

ConditionFiction:LikeFact -0.017436 0.016671 -1.046

>

> PValues_thrilling <- Anova(fullmodelthrilling, type = 3, test = 'Chi')

> PValues_thrilling

Analysis of Deviance Table (Type III Wald chisquare tests)

Response: thrilling

Chisq Df Pr(>Chisq)

(Intercept) 8.2479 1 0.00408 **

Perspective 0.8414 1 0.35900

Condition 0.3425 1 0.55838

P1 140.9753 1 < 2.2e-16 ***

P2 90.7922 1 < 2.2e-16 ***

Gender 0.4718 1 0.49216

Age 20.9163 1 4.798e-06 ***

Education 0.0580 1 0.80964

Dutch 5.2860 1 0.02150 *

LikeFiction 2.7810 1 0.09539 .

LikeFact 1.0709 1 0.30074

Perspective:Condition 0.2955 1 0.58670

Condition:LikeFiction 0.5183 1 0.47157

Condition:LikeFact 1.0939 1 0.29562

---

Signif. codes: 0 ‘***’ 0.001 ‘**’ 0.01 ‘*’ 0.05 ‘.’ 0.1 ‘ ’ 1

> fullmodelbeautiful = lmer (beautiful ~ Perspective*Condition + P1 + P2 + Gender + Age + Education +

Dutch + LikeFiction*Condition + LikeFact*Condition + (1|Story), data=appdata, REML=FALSE)

> summary (fullmodelbeautiful)

Linear mixed model fit by maximum likelihood ['lmerMod']

Formula: beautiful ~ Perspective * Condition + P1 + P2 + Gender + Age +

Education + Dutch + LikeFiction * Condition + LikeFact * Condition + (1 | Story)

Data: appdata

AIC BIC logLik deviance df.resid

5991.1 6078.7 -2979.6 5959.1 1745

Scaled residuals:

Min 1Q Median 3Q Max

-3.2196 -0.6697 0.0328 0.7139 3.4825

Random effects:

Groups Name Variance Std.Dev.

Story (Intercept) 0.06159 0.2482

Residual 1.71542 1.3097

Number of obs: 1761, groups: Story, 4

Fixed effects:

Estimate Std. Error t value

(Intercept) 1.863444 0.323990 5.752

Perspective3rd -0.078080 0.089417 -0.873

ConditionFiction -0.404024 0.266789 -1.514

P1 0.317732 0.022360 14.210

P2 0.265540 0.023391 11.352

Gender2 0.002549 0.072078 0.035

Age -0.007820 0.001950 -4.011

Education 0.048145 0.050689 0.950

DutchTRUE -0.053948 0.105178 -0.513

LikeFiction -0.009852 0.010355 -0.951

LikeFact 0.004717 0.011988 0.393

Perspective3rd:ConditionFiction 0.021498 0.126165 0.170

ConditionFiction:LikeFiction 0.013657 0.014367 0.951

ConditionFiction:LikeFact 0.006848 0.016603 0.412

>

> PValues_beautiful <- Anova(fullmodelbeautiful, type = 3, test = 'Chi')

> PValues_beautiful

Analysis of Deviance Table (Type III Wald chisquare tests)

Response: beautiful

Chisq Df Pr(>Chisq)

(Intercept) 33.0803 1 8.843e-09 ***

Perspective 0.7625 1 0.3825

Condition 2.2934 1 0.1299

P1 201.9104 1 < 2.2e-16 ***

P2 128.8789 1 < 2.2e-16 ***

Gender 0.0013 1 0.9718

Age 16.0850 1 6.056e-05 ***

Education 0.9021 1 0.3422

Dutch 0.2631 1 0.6080

LikeFiction 0.9052 1 0.3414

LikeFact 0.1548 1 0.6940

Perspective:Condition 0.0290 1 0.8647

Condition:LikeFiction 0.9037 1 0.3418

Condition:LikeFact 0.1701 1 0.6800

---

Signif. codes: 0 ‘***’ 0.001 ‘**’ 0.01 ‘*’ 0.05 ‘.’ 0.1 ‘ ’ 1

> fullmodelfascinating = lmer (fascinating ~ Perspective*Condition + P1 + P2 + Gender + Age +Education + Dutch + LikeFiction*Condition + LikeFact*Condition + (1|Story), data=appdata, REML=FALSE)

> summary (fullmodelfascinating)

Linear mixed model fit by maximum likelihood ['lmerMod']

Formula: fascinating ~ Perspective * Condition + P1 + P2 + Gender + Age +

Education + Dutch + LikeFiction * Condition + LikeFact * Condition + (1 | Story)

Data: appdata

AIC BIC logLik deviance df.resid

5873.1 5960.7 -2920.6 5841.1 1745

Scaled residuals:

Min 1Q Median 3Q Max

-3.3646 -0.6603 0.0401 0.7162 3.2433

Random effects:

Groups Name Variance Std.Dev.

Story (Intercept) 0.01321 0.115

Residual 1.60898 1.268

Number of obs: 1761, groups: Story, 4

Fixed effects:

Estimate Std. Error t value

(Intercept) 1.123813 0.295457 3.804

Perspective3rd -0.154940 0.086558 -1.790

ConditionFiction -0.005754 0.258337 -0.022

P1 0.337296 0.021627 15.596

P2 0.313879 0.022625 13.873

Gender2 -0.059409 0.069787 -0.851

Age -0.002117 0.001888 -1.121

Education 0.078237 0.049086 1.594

DutchTRUE 0.015425 0.101820 0.151

LikeFiction -0.013436 0.010027 -1.340

LikeFact 0.017124 0.011607 1.475

Perspective3rd:ConditionFiction 0.014889 0.122036 0.122

ConditionFiction:LikeFiction 0.019940 0.013911 1.433

ConditionFiction:LikeFact -0.020876 0.016076 -1.299

>

> PValues_fascinating <- Anova(fullmodelfascinating, type = 3, test = 'Chi')

> PValues_fascinating

Analysis of Deviance Table (Type III Wald chisquare tests)

Response: fascinating

Chisq Df Pr(>Chisq)

(Intercept) 14.4677 1 0.0001426 ***

Perspective 3.2041 1 0.0734523 .

Condition 0.0005 1 0.9822307

P1 243.2412 1 < 2.2e-16 ***

P2 192.4683 1 < 2.2e-16 ***

Gender 0.7247 1 0.3946093

Age 1.2573 1 0.2621671

Education 2.5404 1 0.1109645

Dutch 0.0230 1 0.8795858

LikeFiction 1.7957 1 0.1802307

LikeFact 2.1767 1 0.1401185

Perspective:Condition 0.0149 1 0.9028924

Condition:LikeFiction 2.0547 1 0.1517376

Condition:LikeFact 1.6862 1 0.1941026

---

Signif. codes: 0 ‘***’ 0.001 ‘**’ 0.01 ‘*’ 0.05 ‘.’ 0.1 ‘ ’ 1

> fullmodelemotional = lmer (emotional ~ Perspective*Condition + P1 + P2 + Gender + Age +

Education + Dutch + LikeFiction*Condition + LikeFact*Condition + (1|Story), data=appdata, REML=FALSE)

> summary (fullmodelemotional)

Linear mixed model fit by maximum likelihood ['lmerMod']

Formula: emotional ~ Perspective * Condition + P1 + P2 + Gender + Age +

Education + Dutch + LikeFiction * Condition + LikeFact * Condition + (1 | Story)

Data: appdata

AIC BIC logLik deviance df.resid

6109.9 6197.5 -3038.9 6077.9 1745

Scaled residuals:

Min 1Q Median 3Q Max

-3.5716 -0.6937 0.0106 0.6957 3.5251

Random effects:

Groups Name Variance Std.Dev.

Story (Intercept) 0.1776 0.4215

Residual 1.8311 1.3532

Number of obs: 1761, groups: Story, 4

Fixed effects:

Estimate Std. Error t value

(Intercept) 1.908083 0.374204 5.099

Perspective3rd -0.063049 0.092393 -0.682

ConditionFiction -0.461946 0.275650 -1.676

P1 0.309544 0.023110 13.395

P2 0.237151 0.024174 9.810

Gender2 0.206295 0.074474 2.770

Age -0.002288 0.002015 -1.135

Education -0.018919 0.052371 -0.361

DutchTRUE -0.132745 0.108678 -1.221

LikeFiction -0.011919 0.010699 -1.114

LikeFact 0.003708 0.012387 0.299

Perspective3rd:ConditionFiction 0.006666 0.130388 0.051

ConditionFiction:LikeFiction 0.036119 0.014844 2.433

ConditionFiction:LikeFact -0.008706 0.017155 -0.508

>

> PValues_emotional <- Anova(fullmodelemotional, type = 3, test = 'Chi')

> PValues_emotional

Analysis of Deviance Table (Type III Wald chisquare tests)

Response: emotional

Chisq Df Pr(>Chisq)

(Intercept) 26.0002 1 3.414e-07 ***

Perspective 0.4657 1 0.494983

Condition 2.8085 1 0.093769 .

P1 179.4172 1 < 2.2e-16 ***

P2 96.2419 1 < 2.2e-16 ***

Gender 7.6732 1 0.005605 **

Age 1.2893 1 0.256173

Education 0.1305 1 0.717912

Dutch 1.4919 1 0.221915

LikeFiction 1.2410 1 0.265270

LikeFact 0.0896 1 0.764684

Perspective:Condition 0.0026 1 0.959230

Condition:LikeFiction 5.9204 1 0.014967 *

Condition:LikeFact 0.2576 1 0.611795

---

Signif. codes: 0 ‘***’ 0.001 ‘**’ 0.01 ‘*’ 0.05 ‘.’ 0.1 ‘ ’ 1

> fullmodelsad = lmer (sad ~ Perspective*Condition + P1 + P2 + Gender + Age + Education + Dutch +

LikeFiction*Condition + LikeFact*Condition + (1|Story), data=appdata, REML=FALSE)

> summary (fullmodelsad)

Linear mixed model fit by maximum likelihood ['lmerMod']

Formula: sad ~ Perspective * Condition + P1 + P2 + Gender + Age + Education +

Dutch + LikeFiction * Condition + LikeFact * Condition + (1 | Story)

Data: appdata

AIC BIC logLik deviance df.resid

6535.9 6623.5 -3251.9 6503.9 1744

Scaled residuals:

Min 1Q Median 3Q Max

-2.2432 -0.7558 -0.1472 0.7192 3.1935

Random effects:

Groups Name Variance Std.Dev.

Story (Intercept) 0.01507 0.1227

Residual 2.35012 1.5330

Number of obs: 1760, groups: Story, 4

Fixed effects:

Estimate Std. Error t value

(Intercept) 4.509328 0.355805 12.674

Perspective3rd 0.311099 0.104598 2.974

ConditionFiction -0.022316 0.313393 -0.071

P1 -0.242003 0.026143 -9.257

P2 -0.111137 0.027334 -4.066

Gender2 0.057797 0.084368 0.685

Age -0.001246 0.002282 -0.546

Education 0.007119 0.059407 0.120

DutchTRUE 0.090027 0.123056 0.732

LikeFiction -0.006425 0.012117 -0.530

LikeFact -0.005558 0.014026 -0.396

Perspective3rd:ConditionFiction -0.314324 0.147468 -2.131

ConditionFiction:LikeFiction -0.003217 0.016824 -0.191

ConditionFiction:LikeFact 0.022890 0.019450 1.177

>

> PValues_sad <- Anova(fullmodelsad, type = 3, test = 'Chi')

> PValues_sad

Analysis of Deviance Table (Type III Wald chisquare tests)

Response: sad

Chisq Df Pr(>Chisq)

(Intercept) 160.6200 1 < 2.2e-16 ***

Perspective 8.8462 1 0.002937 **

Condition 0.0051 1 0.943232

P1 85.6907 1 < 2.2e-16 ***

P2 16.5312 1 4.786e-05 ***

Gender 0.4693 1 0.493309

Age 0.2983 1 0.584934

Education 0.0144 1 0.904620

Dutch 0.5352 1 0.464417

LikeFiction 0.2812 1 0.595947

LikeFact 0.1570 1 0.691910

Perspective:Condition 4.5431 1 0.033051 *

Condition:LikeFiction 0.0366 1 0.848349

Condition:LikeFact 1.3850 1 0.239253

---

Signif. codes: 0 ‘***’ 0.001 ‘**’ 0.01 ‘*’ 0.05 ‘.’ 0.1 ‘ ’ 1

## Picture task

> fullmodelacc1 = lmer (First_person_corr ~ Perspective*Condition + P1 + P2 + Gender + Age + Education + Dutch + LikeFiction*Condition + LikeFact*Condition + (1|Story), data=PTaccdata, REML=TRUE)

> summary (fullmodelacc1)

Linear mixed model fit by REML ['lmerMod']

Formula: First_person_corr ~ Perspective * Condition + P1 + P2 + Gender +

Age + Education + Dutch + LikeFiction * Condition + LikeFact * Condition + (1 | Story)

Data: PTaccdata

REML criterion at convergence: 5731.7

Scaled residuals:

Min 1Q Median 3Q Max

-2.5754 -0.7042 0.5635 0.8367 1.3934

Random effects:

Groups Name Variance Std.Dev.

Story (Intercept) 0.02365 0.1538

Residual 1.64111 1.2811

Number of obs: 1702, groups: Story, 4

Fixed effects:

Estimate Std. Error t value

(Intercept) 0.838188 0.350795 2.389

Perspective3rd -0.019144 0.088713 -0.216

ConditionFiction -0.207698 0.390766 -0.532

P1 0.069478 0.022158 3.135

P2 -0.007801 0.023269 -0.335

Gender2 -0.061245 0.071955 -0.851

Age -0.004100 0.001941 -2.112

Education -0.004918 0.051136 -0.096

DutchTRUE -0.027456 0.106214 -0.258

LikeFiction 0.012182 0.032645 0.373

LikeFact -0.002351 0.041946 -0.056

Perspective3rd:ConditionFiction -0.065348 0.125488 -0.521

ConditionFiction:LikeFiction 0.047057 0.045416 1.036

ConditionFiction:LikeFact 0.024826 0.058864 0.422

> PValues_acc1 <- Anova(fullmodelacc1, type = 3, test = 'F')

> PValues_acc1

Analysis of Deviance Table (Type III Wald F tests with Kenward-Roger df)

Response: First_person_corr

F Df Df.res Pr(>F)

(Intercept) 5.7081 1 628.63 0.017180 *

Perspective 0.0465 1 1687.17 0.829270

Condition 0.2823 1 1686.41 0.595253

P1 9.8005 1 1687.64 0.001775 **

P2 0.1121 1 1687.90 0.737819

Gender 0.7241 1 1686.22 0.394930

Age 4.4616 1 1685.54 0.034812 *

Education 0.0092 1 1685.26 0.923397

Dutch 0.0668 1 1686.84 0.796144

LikeFiction 0.1392 1 1686.13 0.709141

LikeFact 0.0031 1 1686.34 0.955315

Perspective:Condition 0.2704 1 1687.76 0.603156

Condition:LikeFiction 1.0732 1 1685.67 0.300364

Condition:LikeFact 0.1777 1 1686.56 0.673374

---

Signif. codes: 0 ‘***’ 0.001 ‘**’ 0.01 ‘*’ 0.05 ‘.’ 0.1 ‘ ’ 1

> fullmodelacc2 = lmer (Third_person_corr ~ Perspective*Condition + P1 + P2 + Gender + Age + Education + Dutch + LikeFiction*Condition + LikeFact*Condition + (1|Story), data=PTaccdata, REML=TRUE)

> summary (fullmodelacc2)

Linear mixed model fit by REML ['lmerMod']

Formula: Third_person_corr ~ Perspective * Condition + P1 + P2 + Gender +

Age + Education + Dutch + LikeFiction * Condition + LikeFact * Condition + (1 | Story)

Data: PTaccdata

REML criterion at convergence: 5015

Scaled residuals:

Min 1Q Median 3Q Max

-3.7644 -0.6435 0.2844 0.6965 1.4897

Random effects:

Groups Name Variance Std.Dev.

Story (Intercept) 0.1389 0.3727

Residual 1.0694 1.0341

Number of obs: 1702, groups: Story, 4

Fixed effects:

Estimate Std. Error t value

(Intercept) 4.713e-01 3.333e-01 1.414

Perspective3rd -1.554e-02 7.164e-02 -0.217

ConditionFiction 1.874e-01 3.155e-01 0.594

P1 4.992e-02 1.791e-02 2.788

P2 4.817e-02 1.880e-02 2.562

Gender2 -9.378e-02 5.809e-02 -1.614

Age -3.938e-05 1.567e-03 -0.025

Education 3.962e-02 4.128e-02 0.960

DutchTRUE 2.067e-01 8.576e-02 2.410

LikeFiction 1.005e-02 2.636e-02 0.382

LikeFact 2.089e-02 3.387e-02 0.617

Perspective3rd:ConditionFiction -7.471e-02 1.014e-01 -0.737

ConditionFiction:LikeFiction -4.705e-03 3.667e-02 -0.128

ConditionFiction:LikeFact -7.975e-03 4.753e-02 -0.168

> PValues_acc2 <- Anova(fullmodelacc2, type = 3, test = 'F')

> PValues_acc2

Analysis of Deviance Table (Type III Wald F tests with Kenward-Roger df)

Response: Third_person_corr

F Df Df.res Pr(>F)

(Intercept) 2.0000 1 29.31 0.167836

Perspective 0.0470 1 1685.35 0.828314

Condition 0.3527 1 1685.20 0.552688

P1 7.7702 1 1685.95 0.005371 **

P2 6.5623 1 1685.85 0.010502 *

Gender 2.6057 1 1685.17 0.106666

Age 0.0006 1 1685.07 0.979952

Education 0.9214 1 1685.03 0.337245

Dutch 5.8086 1 1685.28 0.016054 *

LikeFiction 0.1455 1 1685.16 0.702925

LikeFact 0.3805 1 1685.19 0.537438

Perspective:Condition 0.5426 1 1685.90 0.461457

Condition:LikeFiction 0.0165 1 1685.09 0.897920

Condition:LikeFact 0.0282 1 1685.23 0.866769

---

Signif. codes: 0 ‘***’ 0.001 ‘**’ 0.01 ‘*’ 0.05 ‘.’ 0.1 ‘ ’ 1

> fullmodelRTs = lmer (First_person_RT ~ First_person_corr + Perspective*Condition + P1 + P2 + Gender + Age + Education + Dutch + LikeFiction*Condition + LikeFact*Condition + (1|Story), data=PTaccdata, REML=TRUE)

> summary (fullmodelRTs)

Linear mixed model fit by REML ['lmerMod']

Formula: First_person_RT ~ First_person_corr + Perspective * Condition +

P1 + P2 + Gender + Age + Education + Dutch + LikeFiction * Condition + LikeFact * Condition + (1 | Story)

Data: PTaccdata

REML criterion at convergence: 30250.2

Scaled residuals:

Min 1Q Median 3Q Max

-2.1606 -0.6327 -0.2274 0.3488 5.7367

Random effects:

Groups Name Variance Std.Dev.

Story (Intercept) 23841 154.4

Residual 3359983 1833.0

Number of obs: 1702, groups: Story, 4

Fixed effects:

Estimate Std. Error t value

(Intercept) 1981.400 496.618 3.990

First_person_corr -139.901 34.803 -4.020

Perspective3rd 131.217 126.897 1.034

ConditionFiction -737.940 559.074 -1.320

P1 27.745 31.766 0.873

P2 56.831 33.270 1.708

Gender2 -166.792 102.964 -1.620

Age 34.529 2.781 12.418

Education -57.461 73.167 -0.785

DutchTRUE -19.221 151.941 -0.127

LikeFiction -38.538 46.706 -0.825

LikeFact 9.075 60.008 0.151

Perspective3rd:ConditionFiction 20.520 179.414 0.114

ConditionFiction:LikeFiction -36.997 65.000 -0.569

ConditionFiction:LikeFact 157.699 84.214 1.873

> PValues_RT1<- Anova(fullmodelRTs, type = 3, test = 'F')

> PValues_RT1

Analysis of Deviance Table (Type III Wald F tests with Kenward-Roger df)

Response: First_person_RT

F Df Df.res Pr(>F)

(Intercept) 15.9140 1 1092.7 7.070e-05 ***

First_person_corr 16.0925 1 1685.3 6.296e-05 ***

Perspective 1.0671 1 1686.9 0.30175

Condition 1.7401 1 1686.3 0.18730

P1 0.7583 1 1679.9 0.38397

P2 2.9040 1 1683.8 0.08854 .

Gender 2.6216 1 1685.9 0.10561

Age 154.1457 1 1685.0 < 2.2e-16 ***

Education 0.6166 1 1684.5 0.43241

Dutch 0.0160 1 1686.7 0.89943

LikeFiction 0.6802 1 1685.8 0.40963

LikeFact 0.0228 1 1686.1 0.87988

Perspective:Condition 0.0130 1 1682.2 0.90920

Condition:LikeFiction 0.3238 1 1685.1 0.56940

Condition:LikeFact 3.5020 1 1686.4 0.06147 .

---

Signif. codes: 0 ‘***’ 0.001 ‘**’ 0.01 ‘*’ 0.05 ‘.’ 0.1 ‘ ’ 1

> fullmodelRTs3 = lmer (Third_person_RT ~ Third_person_corr + Perspective*Condition + P1 + P2 + Gender + Age + Education + Dutch + LikeFiction*Condition + LikeFact*Condition + (1|Story), data=PTaccdata, REML=TRUE)

> summary (fullmodelRTs3)

Linear mixed model fit by REML ['lmerMod']

Formula: Third_person_RT ~ Third_person_corr + Perspective * Condition +

P1 + P2 + Gender + Age + Education + Dutch + LikeFiction * Condition + LikeFact * Condition + (1 | Story)

Data: PTaccdata

REML criterion at convergence: 29977.7

Scaled residuals:

Min 1Q Median 3Q Max

-2.0201 -0.5871 -0.2195 0.3194 9.7221

Random effects:

Groups Name Variance Std.Dev.

Story (Intercept) 232305 482

Residual 2848500 1688

Number of obs: 1702, groups: Story, 4

Fixed effects:

Estimate Std. Error t value

(Intercept) 2123.681 511.645 4.151

Third_person_corr -35.187 39.704 -0.886

Perspective3rd 133.470 116.918 1.142

ConditionFiction -441.805 514.975 -0.858

P1 -13.133 29.287 -0.448

P2 45.259 30.742 1.472

Gender2 -229.198 94.887 -2.415

Age 30.377 2.557 11.879

Education -140.481 67.390 -2.085

DutchTRUE 220.652 140.211 1.574

LikeFiction -170.470 43.017 -3.963

LikeFact 117.513 55.278 2.126

Perspective3rd:ConditionFiction -196.422 165.502 -1.187

ConditionFiction:LikeFiction 21.909 59.840 0.366

ConditionFiction:LikeFact 83.888 77.569 1.081

> PValues_RT3<- Anova(fullmodelRTs3, type = 3, test = 'F')

> PValues_RT3

Analysis of Deviance Table (Type III Wald F tests with Kenward-Roger df)

Response: Third_person_RT

F Df Df.res Pr(>F)

(Intercept) 17.2279 1 56.16 0.0001136 ***

Third_person_corr 0.7825 1 1686.02 0.3765003

Perspective 1.3029 1 1684.54 0.2538468

Condition 0.7359 1 1684.29 0.3910892

P1 0.2009 1 1685.72 0.6540520

P2 2.1659 1 1685.60 0.1412873

Gender 5.8341 1 1684.23 0.0158245 *

Age 141.1113 1 1684.11 < 2.2e-16 ***

Education 4.3455 1 1684.05 0.0372580 *

Dutch 2.4761 1 1684.43 0.1157741

LikeFiction 15.7025 1 1684.24 7.722e-05 ***

LikeFact 4.5187 1 1684.30 0.0336722 *

Perspective:Condition 1.4077 1 1685.37 0.2356113

Condition:LikeFiction 0.1340 1 1684.14 0.7143246

Condition:LikeFact 1.1694 1 1684.36 0.2796832

---

Signif. codes: 0 ‘***’ 0.001 ‘**’ 0.01 ‘*’ 0.05 ‘.’ 0.1 ‘ ’ 1
