# Supplementary material for: When Fiction Is Just as Real as Fact: No Differences in Reading Behavior between Stories Believed to be Based on True or Fictional Events
Source: Front Psychol. 2017 Sep 20;8:1618. doi: 10.3389/fpsyg.2017.01618 (PMC5613255; doi:10.3389/fpsyg.2017.01618)
Supplement: Supplementary file 5 [file DataSheet5.DOCX]

# S5: Statistical models *Reference group*

Contents

[Supplementary material 1: Statistical models 1](#_Toc456108715)

[Reading time 2](#_Toc456108716)

[Perspective taking 3](#_Toc456108717)

[Immersion questionnaire 5](#_Toc456108718)

[Appreciation measure 9](#_Toc456108719)

[Picture task 19](#_Toc456108720)

## Reading time

> fullmodelRT = lmer (ReadingTime ~ Perspective*Condition + P1 + P2 + Gender +

LikeFiction*Condition + LikeFact*Condition + (1|Story), data=cdata, REML=TRUE)

> summary (fullmodelRT)

Linear mixed model fit by REML ['lmerMod']

Formula: ReadingTime ~ Perspective * Condition + P1 + P2 + Gender + LikeFiction *

Condition + LikeFact * Condition + (1 | Story)

Data: cdata

REML criterion at convergence: 920.7

Scaled residuals:

Min 1Q Median 3Q Max

-1.77790 -0.56832 -0.04731 0.51973 1.89968

Random effects:

Groups Name Variance Std.Dev.

Story (Intercept) 3.396e+10 184290

Residual 4.217e+09 64940

Number of obs: 46, groups: Story, 4

Fixed effects:

Estimate Std. Error t value

(Intercept) 326259 153092 2.131

Perspective3rd 15146 27317 0.554

ConditionFiction -132419 164780 -0.804

P1 -1986 8687 -0.229

P2 7555 7575 0.997

Gender2 32286 24053 1.342

LikeFiction -29656 11546 -2.568

LikeFact 1071 17838 0.060

Perspective3rd:ConditionFiction -9535 43255 -0.220

ConditionFiction:LikeFiction 21185 18100 1.170

ConditionFiction:LikeFact 10106 22425 0.451

> PValues_RT <- Anova(fullmodelRT, type = 3, test = 'F')

> PValues_RT

Analysis of Deviance Table (Type III Wald F tests with Kenward-Roger df)

Response: ReadingTime

F Df Df.res Pr(>F)

(Intercept) 4.5374 1 17.324 0.04778 *

Perspective 0.3072 1 32.025 0.58322

Condition 0.6421 1 32.249 0.42881

P1 0.0522 1 32.054 0.82069

P2 0.9937 1 32.050 0.32631

Gender 1.8004 1 32.031 0.18909

LikeFiction 6.5919 1 32.033 0.01512 *

LikeFact 0.0036 1 32.048 0.95251

Perspective:Condition 0.0485 1 32.041 0.82701

Condition:LikeFiction 1.3658 1 32.129 0.25114

Condition:LikeFact 0.2026 1 32.109 0.65565

---

Signif. codes: 0 ‘***’ 0.001 ‘**’ 0.01 ‘*’ 0.05 ‘.’ 0.1 ‘ ’ 1

## Perspective taking

> fullmodelP1 = lmer (P1 ~ Perspective*Condition + Gender + LikeFiction*Condition +

LikeFact*Condition + (1|Story), data=cdata, REML=FALSE)

> summary (fullmodelP1)

Linear mixed model fit by maximum likelihood ['lmerMod']

Formula: P1 ~ Perspective * Condition + Gender + LikeFiction * Condition + LikeFact * Condition + (1 | Story)

Data: cdata

AIC BIC logLik deviance df.resid

167.4 187.5 -72.7 145.4 35

Scaled residuals:

Min 1Q Median 3Q Max

-2.3474 -0.7884 0.3213 0.6340 1.6978

Random effects:

Groups Name Variance Std.Dev.

Story (Intercept) 0.000 0.000

Residual 1.381 1.175

Number of obs: 46, groups: Story, 4

Fixed effects:

Estimate Std. Error t value

(Intercept) 3.40090 2.01742 1.686

Perspective3rd 0.02753 0.48428 0.057

ConditionFiction 1.02499 2.66747 0.384

Gender2 -0.21433 0.42558 -0.504

LikeFiction 0.24579 0.20202 1.217

LikeFact -0.09857 0.31293 -0.315

Perspective3rd:ConditionFiction 0.34490 0.75606 0.456

ConditionFiction:LikeFiction -0.35291 0.30468 -1.158

ConditionFiction:LikeFact 0.15781 0.38545 0.409

>

> PValues_P1 <- Anova(fullmodelP1, type = 3, test = 'Chi')

> PValues_P1

Analysis of Deviance Table (Type III Wald chisquare tests)

Response: P1

Chisq Df Pr(>Chisq)

(Intercept) 2.8418 1 0.09184 .

Perspective 0.0032 1 0.95467

Condition 0.1477 1 0.70079

Gender 0.2536 1 0.61454

LikeFiction 1.4804 1 0.22372

LikeFact 0.0992 1 0.75276

Perspective:Condition 0.2081 1 0.64825

Condition:LikeFiction 1.3416 1 0.24674

Condition:LikeFact 0.1676 1 0.68224

---

Signif. codes: 0 ‘***’ 0.001 ‘**’ 0.01 ‘*’ 0.05 ‘.’ 0.1 ‘ ’ 1

> fullmodelP2 = lmer (P2 ~ Perspective*Condition + Gender + LikeFiction*Condition +

LikeFact*Condition + (1|Story), data=cdata, REML=FALSE)

> summary (fullmodelP2)

Linear mixed model fit by maximum likelihood ['lmerMod']

Formula: P2 ~ Perspective * Condition + Gender + LikeFiction * Condition + LikeFact * Condition + (1 | Story)

Data: cdata

AIC BIC logLik deviance df.resid

180.3 200.4 -79.1 158.3 35

Scaled residuals:

Min 1Q Median 3Q Max

-3.025 -0.622 0.228 0.617 2.173

Random effects:

Groups Name Variance Std.Dev.

Story (Intercept) 6.243e-19 7.901e-10

Residual 1.828e+00 1.352e+00

Number of obs: 46, groups: Story, 4

Fixed effects:

Estimate Std. Error t value

(Intercept) 4.76366 2.32080 2.053

Perspective3rd 0.29608 0.55710 0.532

ConditionFiction 0.02767 3.06860 0.009

Gender2 0.14775 0.48958 0.302

LikeFiction 0.20949 0.23239 0.902

LikeFact -0.20554 0.35999 -0.571

Perspective3rd:ConditionFiction -0.79511 0.86975 -0.914

ConditionFiction:LikeFiction 0.10480 0.35050 0.299

ConditionFiction:LikeFact -0.09381 0.44341 -0.212

>

> PValues_P2 <- Anova(fullmodelP2, type = 3, test = 'Chi')

> PValues_P2

Analysis of Deviance Table (Type III Wald chisquare tests)

Response: P2

Chisq Df Pr(>Chisq)

(Intercept) 4.2131 1 0.04011 *

Perspective 0.2825 1 0.59510

Condition 0.0001 1 0.99281

Gender 0.0911 1 0.76282

LikeFiction 0.8126 1 0.36734

LikeFact 0.3260 1 0.56802

Perspective:Condition 0.8357 1 0.36062

Condition:LikeFiction 0.0894 1 0.76494

Condition:LikeFact 0.0448 1 0.83245

---

Signif. codes: 0 ‘***’ 0.001 ‘**’ 0.01 ‘*’ 0.05 ‘.’ 0.1 ‘ ’ 1

## Immersion questionnaire

> fullmodelATT = lmer (Attention ~ Perspective*Condition + P1 + P2 +Gender +

LikeFiction*Condition + LikeFact*Condition + (1|Story), data=cdata, REML=FALSE)

> summary (fullmodelATT)

Linear mixed model fit by maximum likelihood ['lmerMod']

Formula: Attention ~ Perspective * Condition + P1 + P2 + Gender + LikeFiction *

Condition + LikeFact * Condition + (1 | Story)

Data: cdata

AIC BIC logLik deviance df.resid

125.9 149.7 -49.9 99.9 33

Scaled residuals:

Min 1Q Median 3Q Max

-2.00098 -0.68550 0.00688 0.68957 2.16092

Random effects:

Groups Name Variance Std.Dev.

Story (Intercept) 0.05508 0.2347

Residual 0.47766 0.6911

Number of obs: 46, groups: Story, 4

Fixed effects:

Estimate Std. Error t value

(Intercept) 4.76656 1.28431 3.711

Perspective3rd 0.22493 0.28853 0.780

ConditionFiction -1.88024 1.65145 -1.139

P1 0.17967 0.09113 1.972

P2 0.14444 0.07946 1.818

Gender2 0.10121 0.25383 0.399

LikeFiction -0.07310 0.12193 -0.600

LikeFact -0.29623 0.18737 -1.581

Perspective3rd:ConditionFiction -0.76053 0.45557 -1.669

ConditionFiction:LikeFiction 0.27194 0.18691 1.455

ConditionFiction:LikeFact 0.18172 0.23248 0.782

>

> PValues_ATT <- Anova(fullmodelATT, type = 3, test = 'Chi')

> PValues_ATT

Analysis of Deviance Table (Type III Wald chisquare tests)

Response: Attention

Chisq Df Pr(>Chisq)

(Intercept) 13.7743 1 0.0002061 ***

Perspective 0.6078 1 0.4356352

Condition 1.2963 1 0.2548964

P1 3.8870 1 0.0486605 *

P2 3.3047 1 0.0690836 .

Gender 0.1590 1 0.6901083

LikeFiction 0.3595 1 0.5488104

LikeFact 2.4996 1 0.1138787

Perspective:Condition 2.7869 1 0.0950361 .

Condition:LikeFiction 2.1168 1 0.1456949

Condition:LikeFact 0.6110 1 0.4344029

---

Signif. codes: 0 ‘***’ 0.001 ‘**’ 0.01 ‘*’ 0.05 ‘.’ 0.1 ‘ ’ 1

> fullmodelTRA = lmer (Transportation ~ Perspective*Condition + P1 + P2 + Gender +

LikeFiction*Condition + LikeFact*Condition + (1|Story), data=cdata, REML=FALSE)

> summary (fullmodelTRA)

Linear mixed model fit by maximum likelihood ['lmerMod']

Formula: Transportation ~ Perspective * Condition + P1 + P2 + Gender +

LikeFiction * Condition + LikeFact * Condition + (1 | Story)

Data: cdata

AIC BIC logLik deviance df.resid

132.0 155.8 -53.0 106.0 33

Scaled residuals:

Min 1Q Median 3Q Max

-2.06115 -0.72749 0.04994 0.71426 2.65532

Random effects:

Groups Name Variance Std.Dev.

Story (Intercept) 0.0253 0.1591

Residual 0.5665 0.7526

Number of obs: 46, groups: Story, 4

Fixed effects:

Estimate Std. Error t value

(Intercept) 2.29271 1.38446 1.656

Perspective3rd 0.09366 0.31293 0.299

ConditionFiction -0.52212 1.75721 -0.297

P1 0.21112 0.09859 2.141

P2 0.18110 0.08588 2.109

Gender2 0.02890 0.27532 0.105

LikeFiction -0.10462 0.13239 -0.790

LikeFact 0.02852 0.20279 0.141

Perspective3rd:ConditionFiction -0.70002 0.49392 -1.417

ConditionFiction:LikeFiction 0.10227 0.20127 0.508

ConditionFiction:LikeFact 0.06273 0.25057 0.250

>

> PValues_TRA <- Anova(fullmodelTRA, type = 3, test = 'Chi')

> PValues_TRA

Analysis of Deviance Table (Type III Wald chisquare tests)

Response: Transportation

Chisq Df Pr(>Chisq)

(Intercept) 2.7424 1 0.09771 .

Perspective 0.0896 1 0.76472

Condition 0.0883 1 0.76637

P1 4.5857 1 0.03224 *

P2 4.4468 1 0.03497 *

Gender 0.0110 1 0.91641

LikeFiction 0.6244 1 0.42940

LikeFact 0.0198 1 0.88817

Perspective:Condition 2.0087 1 0.15640

Condition:LikeFiction 0.2582 1 0.61136

Condition:LikeFact 0.0627 1 0.80233

---

Signif. codes: 0 ‘***’ 0.001 ‘**’ 0.01 ‘*’ 0.05 ‘.’ 0.1 ‘ ’ 1

> fullmodelEMO = lmer (EmotionalEngagement ~ Perspective*Condition + P1 + P2 + Gender +

LikeFiction*Condition + LikeFact*Condition + (1|Story), data=cdata, REML=FALSE)

> summary (fullmodelEMO)

Linear mixed model fit by maximum likelihood ['lmerMod']

Formula: EmotionalEngagement ~ Perspective * Condition + P1 + P2 + Gender +

LikeFiction * Condition + LikeFact * Condition + (1 | Story)

Data: cdata

AIC BIC logLik deviance df.resid

111.3 135.0 -42.6 85.3 33

Scaled residuals:

Min 1Q Median 3Q Max

-2.51599 -0.64488 -0.00651 0.65687 2.24353

Random effects:

Groups Name Variance Std.Dev.

Story (Intercept) 0.0000 0.0000

Residual 0.3737 0.6113

Number of obs: 46, groups: Story, 4

Fixed effects:

Estimate Std. Error t value

(Intercept) 3.26386 1.11184 2.936

Perspective3rd 0.35153 0.25268 1.391

ConditionFiction 1.03204 1.38982 0.743

P1 0.39035 0.07939 4.917

P2 -0.04910 0.06901 -0.711

Gender2 0.36397 0.22245 1.636

LikeFiction -0.05840 0.10717 -0.545

LikeFact -0.07295 0.16340 -0.446

Perspective3rd:ConditionFiction -1.54690 0.39898 -3.877

ConditionFiction:LikeFiction 0.29479 0.16142 1.826

ConditionFiction:LikeFact -0.28408 0.20109 -1.413

>

> PValues_EMO <- Anova(fullmodelEMO, type = 3, test = 'Chi')

> PValues_EMO

Analysis of Deviance Table (Type III Wald chisquare tests)

Response: EmotionalEngagement

Chisq Df Pr(>Chisq)

(Intercept) 8.6174 1 0.0033296 **

Perspective 1.9354 1 0.1641712

Condition 0.5514 1 0.4577394

P1 24.1739 1 8.802e-07 ***

P2 0.5061 1 0.4768126

Gender 2.6771 1 0.1018011

LikeFiction 0.2970 1 0.5857866

LikeFact 0.1993 1 0.6552766

Perspective:Condition 15.0320 1 0.0001057 ***

Condition:LikeFiction 3.3350 1 0.0678198 .

Condition:LikeFact 1.9957 1 0.1577426

---

Signif. codes: 0 ‘***’ 0.001 ‘**’ 0.01 ‘*’ 0.05 ‘.’ 0.1 ‘ ’ 1

> fullmodelIMA = lmer (MentalImagery ~ Perspective*Condition + P1 + P2 +Gender +

LikeFiction*Condition + LikeFact*Condition + (1|Story), data=cdata, REML=FALSE)

> summary (fullmodelIMA)

Linear mixed model fit by maximum likelihood ['lmerMod']

Formula: MentalImagery ~ Perspective * Condition + P1 + P2 + Gender +

LikeFiction * Condition + LikeFact * Condition + (1 | Story)

Data: cdata

AIC BIC logLik deviance df.resid

126.9 150.7 -50.4 100.9 33

Scaled residuals:

Min 1Q Median 3Q Max

-1.7853 -0.6515 -0.1708 0.7684 2.0749

Random effects:

Groups Name Variance Std.Dev.

Story (Intercept) 0.05432 0.2331

Residual 0.48905 0.6993

Number of obs: 46, groups: Story, 4

Fixed effects:

Estimate Std. Error t value

(Intercept) 3.04183 1.29892 2.342

Perspective3rd 0.60791 0.29190 2.083

ConditionFiction -2.93967 1.66930 -1.761

P1 0.21652 0.09218 2.349

P2 0.32387 0.08038 4.029

Gender2 0.28963 0.25680 1.128

LikeFiction -0.05198 0.12336 -0.421

LikeFact -0.18068 0.18954 -0.953

Perspective3rd:ConditionFiction -1.23953 0.46088 -2.689

ConditionFiction:LikeFiction 0.26009 0.18903 1.376

ConditionFiction:LikeFact 0.47056 0.23512 2.001

>

> PValues_IMA <- Anova(fullmodelIMA, type = 3, test = 'Chi')

> PValues_IMA

Analysis of Deviance Table (Type III Wald chisquare tests)

Response: MentalImagery

Chisq Df Pr(>Chisq)

(Intercept) 5.4841 1 0.019190 *

Perspective 4.3372 1 0.037288 *

Condition 3.1012 1 0.078236 .

P1 5.5169 1 0.018834 *

P2 16.2362 1 5.592e-05 ***

Gender 1.2721 1 0.259381

LikeFiction 0.1775 1 0.673490

LikeFact 0.9087 1 0.340461

Perspective:Condition 7.2333 1 0.007157 **

Condition:LikeFiction 1.8931 1 0.168852

Condition:LikeFact 4.0053 1 0.045357 *

---

Signif. codes: 0 ‘***’ 0.001 ‘**’ 0.01 ‘*’ 0.05 ‘.’ 0.1 ‘ ’ 1

## Appreciation measure

fullmodelinteresting = lmer (interesting ~ Perspective*Condition + P1 + P2 +Gender + LikeFiction*Condition + LikeFact*Condition + (1|Story), data=cdata, REML=FALSE)

> summary (fullmodelinteresting)

Linear mixed model fit by maximum likelihood ['lmerMod']

Formula: interesting ~ Perspective * Condition + P1 + P2 + Gender + LikeFiction *

Condition + LikeFact * Condition + (1 | Story)

Data: cdata

AIC BIC logLik deviance df.resid

167.9 191.7 -71.0 141.9 33

Scaled residuals:

Min 1Q Median 3Q Max

-1.9024 -0.5663 -0.2269 0.5222 2.4728

Random effects:

Groups Name Variance Std.Dev.

Story (Intercept) 0.2698 0.5194

Residual 1.1447 1.0699

Number of obs: 46, groups: Story, 4

Fixed effects:

Estimate Std. Error t value

(Intercept) 4.672930 2.008589 2.326

Perspective3rd -1.102967 0.447941 -2.462

ConditionFiction 0.608669 2.607137 0.234

P1 0.075400 0.141791 0.532

P2 0.191040 0.123670 1.545

Gender2 -0.325353 0.394141 -0.826

LikeFiction -0.009232 0.189230 -0.049

LikeFact -0.161218 0.291421 -0.553

Perspective3rd:ConditionFiction -0.327833 0.707761 -0.463

ConditionFiction:LikeFiction 0.183525 0.292160 0.628

ConditionFiction:LikeFact -0.165634 0.362996 -0.456

> PValues_interesting <- Anova(fullmodelinteresting, type = 3, test = 'Chi')

> PValues_interesting

Analysis of Deviance Table (Type III Wald chisquare tests)

Response: interesting

Chisq Df Pr(>Chisq)

(Intercept) 5.4125 1 0.01999 *

Perspective 6.0629 1 0.01380 *

Condition 0.0545 1 0.81540

P1 0.2828 1 0.59488

P2 2.3863 1 0.12240

Gender 0.6814 1 0.40910

LikeFiction 0.0024 1 0.96109

LikeFact 0.3060 1 0.58012

Perspective:Condition 0.2146 1 0.64322

Condition:LikeFiction 0.3946 1 0.52990

Condition:LikeFact 0.2082 1 0.64818

---

Signif. codes: 0 ‘***’ 0.001 ‘**’ 0.01 ‘*’ 0.05 ‘.’ 0.1 ‘ ’ 1

> fullmodelwellwritten = lmer (wellwritten ~ Perspective*Condition + P1 + P2+ Gender + LikeFiction*Condition + LikeFact*Condition + (1|Story), data=cdata, REML=FALSE)

> summary (fullmodelwellwritten)

Linear mixed model fit by maximum likelihood ['lmerMod']

Formula: wellwritten ~ Perspective * Condition + P1 + P2 + Gender + LikeFiction *

Condition + LikeFact * Condition + (1 | Story)

Data: cdata

AIC BIC logLik deviance df.resid

166.5 190.3 -70.3 140.5 33

Scaled residuals:

Min 1Q Median 3Q Max

-3.4585 -0.4201 0.0912 0.4916 2.0145

Random effects:

Groups Name Variance Std.Dev.

Story (Intercept) 0.06703 0.2589

Residual 1.19047 1.0911

Number of obs: 46, groups: Story, 4

Fixed effects:

Estimate Std. Error t value

(Intercept) 6.865437 2.011280 3.413

Perspective3rd -0.502357 0.454079 -1.106

ConditionFiction -2.330794 2.560057 -0.910

P1 0.001489 0.143131 0.010

P2 0.224658 0.124718 1.801

Gender2 0.709164 0.399489 1.775

LikeFiction -0.100856 0.192046 -0.525

LikeFact -0.542069 0.294389 -1.841

Perspective3rd:ConditionFiction -0.738862 0.716722 -1.031

ConditionFiction:LikeFiction 0.202386 0.292479 0.692

ConditionFiction:LikeFact 0.495916 0.364057 1.362

>

> PValues_wellwritten <- Anova(fullmodelwellwritten, type = 3, test = 'Chi')

> PValues_wellwritten

Analysis of Deviance Table (Type III Wald chisquare tests)

Response: wellwritten

Chisq Df Pr(>Chisq)

(Intercept) 11.6518 1 0.0006414 ***

Perspective 1.2239 1 0.2685878

Condition 0.8289 1 0.3625873

P1 0.0001 1 0.9917015

P2 3.2448 1 0.0716505 .

Gender 3.1513 1 0.0758685 .

LikeFiction 0.2758 1 0.5994678

LikeFact 3.3905 1 0.0655719 .

Perspective:Condition 1.0627 1 0.3025922

Condition:LikeFiction 0.4788 1 0.4889567

Condition:LikeFact 1.8556 1 0.1731372

---

Signif. codes: 0 ‘***’ 0.001 ‘**’ 0.01 ‘*’ 0.05 ‘.’ 0.1 ‘ ’ 1

> fullmodelliterary = lmer (literary ~ Perspective*Condition + P1 + P2+ Gender + LikeFiction*Condition + LikeFact*Condition + (1|Story), data=cdata, REML=FALSE)

> summary (fullmodelliterary)

Linear mixed model fit by maximum likelihood ['lmerMod']

Formula: literary ~ Perspective * Condition + P1 + P2 + Gender + LikeFiction *

Condition + LikeFact * Condition + (1 | Story)

Data: cdata

AIC BIC logLik deviance df.resid

165.6 189.4 -69.8 139.6 33

Scaled residuals:

Min 1Q Median 3Q Max

-2.0319 -0.6026 -0.0957 0.5778 2.6489

Random effects:

Groups Name Variance Std.Dev.

Story (Intercept) 0.4729 0.6877

Residual 1.0417 1.0206

Number of obs: 46, groups: Story, 4

Fixed effects:

Estimate Std. Error t value

(Intercept) 4.67137 1.93921 2.409

Perspective3rd -0.86901 0.42816 -2.030

ConditionFiction -2.42949 2.52580 -0.962

P1 0.26024 0.13577 1.917

P2 0.20416 0.11842 1.724

Gender2 0.20558 0.37682 0.546

LikeFiction -0.23626 0.18088 -1.306

LikeFact -0.21190 0.27895 -0.760

Perspective3rd:ConditionFiction 0.20244 0.67699 0.299

ConditionFiction:LikeFiction 0.50396 0.28087 1.794

ConditionFiction:LikeFact -0.07013 0.34863 -0.201

>

> PValues_literary <- Anova(fullmodelliterary, type = 3, test = 'Chi')

> PValues_literary

Analysis of Deviance Table (Type III Wald chisquare tests)

Response: literary

Chisq Df Pr(>Chisq)

(Intercept) 5.8028 1 0.01600 *

Perspective 4.1195 1 0.04239 *

Condition 0.9252 1 0.33612

P1 3.6740 1 0.05527 .

P2 2.9725 1 0.08469 .

Gender 0.2977 1 0.58536

LikeFiction 1.7061 1 0.19149

LikeFact 0.5771 1 0.44747

Perspective:Condition 0.0894 1 0.76492

Condition:LikeFiction 3.2195 1 0.07277 .

Condition:LikeFact 0.0405 1 0.84057

---

Signif. codes: 0 ‘***’ 0.001 ‘**’ 0.01 ‘*’ 0.05 ‘.’ 0.1 ‘ ’ 1

> fullmodeleasytounderstand = lmer (easytounderstand ~ Perspective*Condition + P1 + P2+ Gender + LikeFiction*Condition + LikeFact*Condition + (1|Story), data=cdata, REML=FALSE)

> summary (fullmodeleasytounderstand)

Linear mixed model fit by maximum likelihood ['lmerMod']

Formula: easytounderstand ~ Perspective * Condition + P1 + P2 + Gender +

LikeFiction * Condition + LikeFact * Condition + (1 | Story)

Data: cdata

AIC BIC logLik deviance df.resid

165.7 189.4 -69.8 139.7 33

Scaled residuals:

Min 1Q Median 3Q Max

-1.93418 -0.77229 -0.02553 0.66390 1.79823

Random effects:

Groups Name Variance Std.Dev.

Story (Intercept) 0.1202 0.3467

Residual 1.1388 1.0671

Number of obs: 46, groups: Story, 4

Fixed effects:

Estimate Std. Error t value

(Intercept) 4.13718 1.98082 2.089

Perspective3rd -0.36622 0.44532 -0.822

ConditionFiction 1.19126 2.54374 0.468

P1 -0.05292 0.14061 -0.376

P2 0.08737 0.12260 0.713

Gender2 0.13049 0.39177 0.333

LikeFiction 0.33273 0.18821 1.768

LikeFact -0.25240 0.28913 -0.873

Perspective3rd:ConditionFiction -0.74453 0.70310 -1.059

ConditionFiction:LikeFiction -0.34014 0.28826 -1.180

ConditionFiction:LikeFact 0.23460 0.35857 0.654

>

> PValues_easytounderstand <- Anova(fullmodeleasytounderstand, type = 3, test = 'Chi')

> PValues_easytounderstand

Analysis of Deviance Table (Type III Wald chisquare tests)

Response: easytounderstand

Chisq Df Pr(>Chisq)

(Intercept) 4.3623 1 0.03674 *

Perspective 0.6763 1 0.41086

Condition 0.2193 1 0.63956

P1 0.1417 1 0.70664

P2 0.5078 1 0.47608

Gender 0.1109 1 0.73908

LikeFiction 3.1256 1 0.07707 .

LikeFact 0.7621 1 0.38267

Perspective:Condition 1.1213 1 0.28964

Condition:LikeFiction 1.3923 1 0.23801

Condition:LikeFact 0.4281 1 0.51294

---

Signif. codes: 0 ‘***’ 0.001 ‘**’ 0.01 ‘*’ 0.05 ‘.’ 0.1 ‘ ’ 1

> fullmodelaccessible = lmer (accessible ~ Perspective*Condition + P1 + P2+ Gender + LikeFiction*Condition + LikeFact*Condition + (1|Story), data=cdata, REML=FALSE)

> summary (fullmodelaccessible)

Linear mixed model fit by maximum likelihood ['lmerMod']

Formula: accessible ~ Perspective * Condition + P1 + P2 + Gender + LikeFiction *

Condition + LikeFact * Condition + (1 | Story)

Data: cdata

AIC BIC logLik deviance df.resid

153.9 177.7 -63.9 127.9 33

Scaled residuals:

Min 1Q Median 3Q Max

-2.3130 -0.4067 0.1930 0.5050 1.7920

Random effects:

Groups Name Variance Std.Dev.

Story (Intercept) 0.0571 0.2390

Residual 0.9004 0.9489

Number of obs: 46, groups: Story, 4

Fixed effects:

Estimate Std. Error t value

(Intercept) 2.85315 1.75120 1.629

Perspective3rd 0.47469 0.39510 1.201

ConditionFiction -0.55315 2.23252 -0.248

P1 0.31487 0.12458 2.527

P2 0.18680 0.10856 1.721

Gender2 0.39222 0.34759 1.128

LikeFiction 0.01500 0.16707 0.090

LikeFact -0.12090 0.25621 -0.472

Perspective3rd:ConditionFiction -1.30776 0.62364 -2.097

ConditionFiction:LikeFiction 0.05143 0.25470 0.202

ConditionFiction:LikeFact 0.24803 0.31700 0.782

> PValues_accessible <- Anova(fullmodelaccessible, type = 3, test = 'Chi')

> PValues_accessible

Analysis of Deviance Table (Type III Wald chisquare tests)

Response: accessible

Chisq Df Pr(>Chisq)

(Intercept) 2.6545 1 0.10326

Perspective 1.4435 1 0.22958

Condition 0.0614 1 0.80431

P1 6.3885 1 0.01149 *

P2 2.9608 1 0.08531 .

Gender 1.2733 1 0.25915

LikeFiction 0.0081 1 0.92848

LikeFact 0.2227 1 0.63702

Perspective:Condition 4.3973 1 0.03600 *

Condition:LikeFiction 0.0408 1 0.83998

Condition:LikeFact 0.6122 1 0.43395

---

Signif. codes: 0 ‘***’ 0.001 ‘**’ 0.01 ‘*’ 0.05 ‘.’ 0.1 ‘ ’ 1

> fullmodelthrilling = lmer (thrilling ~ Perspective*Condition + P1 + P2 + Gender + LikeFiction*Condition + LikeFact*Condition + (1|Story), data=cdata, REML=FALSE)

> summary (fullmodelthrilling)

Linear mixed model fit by maximum likelihood ['lmerMod']

Formula: thrilling ~ Perspective * Condition + P1 + P2 + Gender + LikeFiction *

Condition + LikeFact * Condition + (1 | Story)

Data: cdata

AIC BIC logLik deviance df.resid

173.2 196.9 -73.6 147.2 33

Scaled residuals:

Min 1Q Median 3Q Max

-1.8756 -0.8081 -0.1163 0.7301 2.0819

Random effects:

Groups Name Variance Std.Dev.

Story (Intercept) 0.1029 0.3208

Residual 1.3596 1.1660

Number of obs: 46, groups: Story, 4

Fixed effects:

Estimate Std. Error t value

(Intercept) 3.90980 2.15602 1.813

Perspective3rd -0.57812 0.48589 -1.190

ConditionFiction -2.87221 2.75535 -1.042

P1 0.02371 0.15328 0.155

P2 0.17292 0.13360 1.294

Gender2 -0.06534 0.42746 -0.153

LikeFiction -0.40547 0.20542 -1.974

LikeFact 0.14296 0.31521 0.454

Perspective3rd:ConditionFiction 0.77989 0.76700 1.017

ConditionFiction:LikeFiction 0.51364 0.31364 1.638

ConditionFiction:LikeFact -0.07994 0.39029 -0.205

>

> PValues_thrilling <- Anova(fullmodelthrilling, type = 3, test = 'Chi')

> PValues_thrilling

Analysis of Deviance Table (Type III Wald chisquare tests)

Response: thrilling

Chisq Df Pr(>Chisq)

(Intercept) 3.2886 1 0.06976 .

Perspective 1.4157 1 0.23412

Condition 1.0866 1 0.29722

P1 0.0239 1 0.87709

P2 1.6752 1 0.19556

Gender 0.0234 1 0.87852

LikeFiction 3.8960 1 0.04840 *

LikeFact 0.2057 1 0.65017

Perspective:Condition 1.0339 1 0.30925

Condition:LikeFiction 2.6820 1 0.10149

Condition:LikeFact 0.0420 1 0.83770

---

Signif. codes: 0 ‘***’ 0.001 ‘**’ 0.01 ‘*’ 0.05 ‘.’ 0.1 ‘ ’ 1

> fullmodelbeautiful = lmer (beautiful ~ Perspective*Condition + P1 + P2+ Gender + LikeFiction*Condition + LikeFact*Condition + (1|Story), data=cdata, REML=FALSE)

> summary (fullmodelbeautiful)

Linear mixed model fit by maximum likelihood ['lmerMod']

Formula: beautiful ~ Perspective * Condition + P1 + P2 + Gender + LikeFiction *

Condition + LikeFact * Condition + (1 | Story)

Data: cdata

AIC BIC logLik deviance df.resid

157.1 180.8 -65.5 131.1 33

Scaled residuals:

Min 1Q Median 3Q Max

-2.24380 -0.72369 -0.08082 0.95190 1.77392

Random effects:

Groups Name Variance Std.Dev.

Story (Intercept) 0.5317 0.7292

Residual 0.8438 0.9186

Number of obs: 46, groups: Story, 4

Fixed effects:

Estimate Std. Error t value

(Intercept) 2.27983 1.75877 1.296

Perspective3rd -0.67745 0.38562 -1.757

ConditionFiction 0.16548 2.28719 0.072

P1 0.29071 0.12236 2.376

P2 0.21729 0.10672 2.036

Gender2 0.03260 0.33942 0.096

LikeFiction 0.11949 0.16292 0.733

LikeFact -0.04801 0.25137 -0.191

Perspective3rd:ConditionFiction -0.17410 0.60993 -0.285

ConditionFiction:LikeFiction 0.15279 0.25357 0.602

ConditionFiction:LikeFact -0.07556 0.31460 -0.240

> PValues_beautiful <- Anova(fullmodelbeautiful, type = 3, test = 'Chi')

> PValues_beautiful

Analysis of Deviance Table (Type III Wald chisquare tests)

Response: beautiful

Chisq Df Pr(>Chisq)

(Intercept) 1.6803 1 0.19488

Perspective 3.0863 1 0.07896 .

Condition 0.0052 1 0.94232

P1 5.6444 1 0.01751 *

P2 4.1451 1 0.04175 *

Gender 0.0092 1 0.92347

LikeFiction 0.5379 1 0.46329

LikeFact 0.0365 1 0.84855

Perspective:Condition 0.0815 1 0.77531

Condition:LikeFiction 0.3631 1 0.54681

Condition:LikeFact 0.0577 1 0.81019

---

Signif. codes: 0 ‘***’ 0.001 ‘**’ 0.01 ‘*’ 0.05 ‘.’ 0.1 ‘ ’ 1

> fullmodelfascinating = lmer (fascinating ~ Perspective*Condition + P1 + P2+ Gender + LikeFiction*Condition + LikeFact*Condition + (1|Story), data=cdata, REML=FALSE)

> summary (fullmodelfascinating)

Linear mixed model fit by maximum likelihood ['lmerMod']

Formula: fascinating ~ Perspective * Condition + P1 + P2 + Gender + LikeFiction *

Condition + LikeFact * Condition + (1 | Story)

Data: cdata

AIC BIC logLik deviance df.resid

169.6 193.3 -71.8 143.6 33

Scaled residuals:

Min 1Q Median 3Q Max

-2.5756 -0.6354 -0.1111 0.6722 1.8921

Random effects:

Groups Name Variance Std.Dev.

Story (Intercept) 0.3879 0.6228

Residual 1.1591 1.0766

Number of obs: 46, groups: Story, 4

Fixed effects:

Estimate Std. Error t value

(Intercept) 4.07521 2.03326 2.004

Perspective3rd -0.06492 0.45125 -0.144

ConditionFiction -3.53627 2.64646 -1.336

P1 0.24207 0.14298 1.693

P2 0.13045 0.12471 1.046

Gender2 -0.18849 0.39710 -0.475

LikeFiction -0.01704 0.19063 -0.089

LikeFact -0.17295 0.29382 -0.589

Perspective3rd:ConditionFiction -1.89381 0.71328 -2.655

ConditionFiction:LikeFiction 0.43159 0.29527 1.462

ConditionFiction:LikeFact 0.43695 0.36666 1.192

>

> PValues_fascinating <- Anova(fullmodelfascinating, type = 3, test = 'Chi')

> PValues_fascinating

Analysis of Deviance Table (Type III Wald chisquare tests)

Response: fascinating

Chisq Df Pr(>Chisq)

(Intercept) 4.0171 1 0.045041 *

Perspective 0.0207 1 0.885609

Condition 1.7855 1 0.181476

P1 2.8662 1 0.090457 .

P2 1.0941 1 0.295570

Gender 0.2253 1 0.635019

LikeFiction 0.0080 1 0.928773

LikeFact 0.3465 1 0.556100

Perspective:Condition 7.0494 1 0.007929 **

Condition:LikeFiction 2.1365 1 0.143829

Condition:LikeFact 1.4202 1 0.233376

---

Signif. codes: 0 ‘***’ 0.001 ‘**’ 0.01 ‘*’ 0.05 ‘.’ 0.1 ‘ ’ 1

> fullmodelemotional = lmer (emotional ~ Perspective*Condition + P1 + P2+ Gender + LikeFiction*Condition + LikeFact*Condition + (1|Story), data=cdata, REML=FALSE)

> summary (fullmodelemotional)

Linear mixed model fit by maximum likelihood ['lmerMod']

Formula: emotional ~ Perspective * Condition + P1 + P2 + Gender + LikeFiction *

Condition + LikeFact * Condition + (1 | Story)

Data: cdata

AIC BIC logLik deviance df.resid

165.9 189.7 -70.0 139.9 33

Scaled residuals:

Min 1Q Median 3Q Max

-2.01943 -0.66703 0.03529 0.66614 1.93740

Random effects:

Groups Name Variance Std.Dev.

Story (Intercept) 0.7826 0.8846

Residual 1.0065 1.0032

Number of obs: 46, groups: Story, 4

Fixed effects:

Estimate Std. Error t value

(Intercept) 2.6169 1.9322 1.354

Perspective3rd 0.6786 0.4213 1.611

ConditionFiction 1.6863 2.5063 0.673

P1 0.2199 0.1337 1.644

P2 0.1046 0.1166 0.896

Gender2 0.1693 0.3709 0.456

LikeFiction -0.2036 0.1780 -1.143

LikeFact 0.2026 0.2747 0.737

Perspective3rd:ConditionFiction -1.6589 0.6665 -2.489

ConditionFiction:LikeFiction 0.5452 0.2774 1.966

ConditionFiction:LikeFact -0.6666 0.3441 -1.937

>

> PValues_emotional <- Anova(fullmodelemotional, type = 3, test = 'Chi')

> PValues_emotional

Analysis of Deviance Table (Type III Wald chisquare tests)

Response: emotional

Chisq Df Pr(>Chisq)

(Intercept) 1.8344 1 0.17560

Perspective 2.5942 1 0.10726

Condition 0.4527 1 0.50106

P1 2.7030 1 0.10016

P2 0.8036 1 0.37003

Gender 0.2084 1 0.64805

LikeFiction 1.3076 1 0.25284

LikeFact 0.5436 1 0.46093

Perspective:Condition 6.1945 1 0.01281 *

Condition:LikeFiction 3.8631 1 0.04936 *

Condition:LikeFact 3.7535 1 0.05270 .

---

Signif. codes: 0 ‘***’ 0.001 ‘**’ 0.01 ‘*’ 0.05 ‘.’ 0.1 ‘ ’ 1

> fullmodelsad = lmer (sad ~ Perspective*Condition + P1 + P2+ Gender + LikeFiction*Condition + LikeFact*Condition + (1|Story), data=cdata, REML=FALSE)

> summary (fullmodelsad)

Linear mixed model fit by maximum likelihood ['lmerMod']

Formula: sad ~ Perspective * Condition + P1 + P2 + Gender + LikeFiction *

Condition + LikeFact * Condition + (1 | Story)

Data: cdata

AIC BIC logLik deviance df.resid

169.1 192.9 -71.6 143.1 33

Scaled residuals:

Min 1Q Median 3Q Max

-1.5608 -0.8059 -0.1714 0.5552 2.4961

Random effects:

Groups Name Variance Std.Dev.

Story (Intercept) 0.000 0.000

Residual 1.314 1.146

Number of obs: 46, groups: Story, 4

Fixed effects:

Estimate Std. Error t value

(Intercept) 0.15030 2.08483 0.072

Perspective3rd 0.65812 0.47381 1.389

ConditionFiction 1.70892 2.60606 0.656

P1 -0.26152 0.14887 -1.757

P2 0.09019 0.12941 0.697

Gender2 -0.81853 0.41712 -1.962

LikeFiction 0.07677 0.20095 0.382

LikeFact 0.56322 0.30639 1.838

Perspective3rd:ConditionFiction 0.50834 0.74814 0.680

ConditionFiction:LikeFiction -0.22273 0.30268 -0.736

ConditionFiction:LikeFact -0.19079 0.37706 -0.506

>

> PValues_sad <- Anova(fullmodelsad, type = 3, test = 'Chi')

> PValues_sad

Analysis of Deviance Table (Type III Wald chisquare tests)

Response: sad

Chisq Df Pr(>Chisq)

(Intercept) 0.0052 1 0.94253

Perspective 1.9293 1 0.16483

Condition 0.4300 1 0.51199

P1 3.0860 1 0.07897 .

P2 0.4857 1 0.48586

Gender 3.8508 1 0.04972 *

LikeFiction 0.1459 1 0.70244

LikeFact 3.3792 1 0.06602 .

Perspective:Condition 0.4617 1 0.49683

Condition:LikeFiction 0.5415 1 0.46181

Condition:LikeFact 0.2560 1 0.61287

---

Signif. codes: 0 ‘***’ 0.001 ‘**’ 0.01 ‘*’ 0.05 ‘.’ 0.1 ‘ ’ 1

## Picture task

> fullmodelacc1 = lmer (First_person_corr ~ Perspective*Condition + P1 + P2 + Gender +

LikeFiction*Condition + LikeFact*Condition + (1|Story), data=cdata, REML=TRUE)

> summary (fullmodelacc1)

Linear mixed model fit by REML ['lmerMod']

Formula: First_person_corr ~ Perspective * Condition + P1 + P2 + Gender +

LikeFiction * Condition + LikeFact * Condition + (1 | Story)

Data: cdata

REML criterion at convergence: 129.9

Scaled residuals:

Min 1Q Median 3Q Max

-1.9613 -0.7823 0.2494 0.6418 1.6376

Random effects:

Groups Name Variance Std.Dev.

Story (Intercept) 0.0367 0.1916

Residual 0.9102 0.9541

Number of obs: 46, groups: Story, 4

Fixed effects:

Estimate Std. Error t value

(Intercept) 0.936870 1.753503 0.534

Perspective3rd -0.493897 0.396528 -1.246

ConditionFiction -3.072956 2.223032 -1.382

P1 0.098244 0.124897 0.787

P2 -0.096507 0.108789 -0.887

Gender2 -0.703593 0.348880 -2.017

LikeFiction 0.236346 0.167780 1.409

LikeFact -0.036819 0.256919 -0.143

Perspective3rd:ConditionFiction 0.008389 0.625858 0.013

ConditionFiction:LikeFiction 0.366981 0.254895 1.440

ConditionFiction:LikeFact 0.121346 0.317350 0.382

>

> PValues_acc1 <- Anova(fullmodelacc1, type = 3, test = 'F')

> PValues_acc1

Analysis of Deviance Table (Type III Wald F tests with Kenward-Roger df)

Response: First_person_corr

F Df Df.res Pr(>F)

(Intercept) 0.2663 1 34.786 0.60909

Perspective 1.4781 1 33.691 0.23252

Condition 1.6380 1 33.328 0.20944

P1 0.5806 1 34.312 0.45128

P2 0.7229 1 34.576 0.40105

Gender 3.9063 1 33.608 0.05636 .

LikeFiction 1.9403 1 33.181 0.17290

LikeFact 0.0193 1 34.214 0.89020

Perspective:Condition 0.0002 1 33.699 0.98959

Condition:LikeFiction 1.9171 1 34.916 0.17496

Condition:LikeFact 0.1352 1 34.850 0.71531

---

Signif. codes: 0 ‘***’ 0.001 ‘**’ 0.01 ‘*’ 0.05 ‘.’ 0.1 ‘ ’ 1

>

> fullmodelacc2 = lmer (Third_person_corr ~ Perspective*Condition + P1 + P2 + Gender +

LikeFiction*Condition + LikeFact*Condition + (1|Story), data=cdata, REML=TRUE)

> summary (fullmodelacc2)

Linear mixed model fit by REML ['lmerMod']

Formula: Third_person_corr ~ Perspective * Condition + P1 + P2 + Gender +

LikeFiction * Condition + LikeFact * Condition + (1 | Story)

Data: cdata

REML criterion at convergence: 111

Scaled residuals:

Min 1Q Median 3Q Max

-1.9311 -0.7051 0.1518 0.7299 1.2812

Random effects:

Groups Name Variance Std.Dev.

Story (Intercept) 0.1497 0.3869

Residual 0.4873 0.6980

Number of obs: 46, groups: Story, 4

Fixed effects:

Estimate Std. Error t value

(Intercept) 3.08887 1.31630 2.347

Perspective3rd -0.36627 0.29251 -1.252

ConditionFiction -2.33616 1.71242 -1.364

P1 -0.22961 0.09266 -2.478

P2 0.26024 0.08082 3.220

Gender2 -0.19041 0.25740 -0.740

LikeFiction -0.06288 0.12357 -0.509

LikeFact -0.22446 0.19042 -1.179

Perspective3rd:ConditionFiction 0.85234 0.46231 1.844

ConditionFiction:LikeFiction -0.13147 0.19125 -0.687

ConditionFiction:LikeFact 0.47739 0.23752 2.010

> PValues_acc2 <- Anova(fullmodelacc2, type = 3, test = 'F')

> PValues_acc2

Analysis of Deviance Table (Type III Wald F tests with Kenward-Roger df)

Response: Third_person_corr

F Df Df.res Pr(>F)

(Intercept) 5.3600 1 34.417 0.026694 *

Perspective 1.5471 1 32.549 0.222444

Condition 1.7084 1 34.854 0.199749

P1 5.9974 1 32.988 0.019809 *

P2 10.1136 1 32.995 0.003196 **

Gender 0.5395 1 32.605 0.467884

LikeFiction 0.2560 1 32.553 0.616313

LikeFact 1.3595 1 32.907 0.252004

Perspective:Condition 3.3432 1 32.731 0.076608 .

Condition:LikeFiction 0.4523 1 33.843 0.505817

Condition:LikeFact 3.8840 1 33.651 0.057018 .

---

Signif. codes: 0 ‘***’ 0.001 ‘**’ 0.01 ‘*’ 0.05 ‘.’ 0.1 ‘ ’ 1

> fullmodelRTs = lmer (First_person_RT ~ First_person_corr + Perspective*Condition +

P1 + P2 + Gender + LikeFiction*Condition + LikeFact*Condition + (1|Story), data=cdata,

REML=TRUE)

> summary (fullmodelRTs)

Linear mixed model fit by REML ['lmerMod']

Formula: First_person_RT ~ First_person_corr + Perspective * Condition +

P1 + P2 + Gender + LikeFiction * Condition + LikeFact * Condition + (1 | Story)

Data: cdata

REML criterion at convergence: 614.7

Scaled residuals:

Min 1Q Median 3Q Max

-1.55976 -0.47784 -0.06205 0.28725 3.02307

Random effects:

Groups Name Variance Std.Dev.

Story (Intercept) 1.095e-09 3.309e-05

Residual 1.424e+06 1.193e+03

Number of obs: 46, groups: Story, 4

Fixed effects:

Estimate Std. Error t value

(Intercept) -558.420 2178.081 -0.256

First_person_corr -312.464 208.482 -1.499

Perspective3rd 785.665 503.246 1.561

ConditionFiction 2.959 2799.604 0.001

P1 42.560 156.709 0.272

P2 146.650 136.308 1.076

Gender2 -308.754 459.763 -0.672

LikeFiction -100.371 214.766 -0.467

LikeFact 525.016 318.976 1.646

Perspective3rd:ConditionFiction -609.347 778.805 -0.782

ConditionFiction:LikeFiction -30.494 325.275 -0.094

ConditionFiction:LikeFact 121.978 393.503 0.310

>

> PValues_RT1<- Anova(fullmodelRTs, type = 3, test = 'F')

> PValues_RT1

Analysis of Deviance Table (Type III Wald F tests with Kenward-Roger df)

Response: First_person_RT

F Df Df.res Pr(>F)

(Intercept) 0.0594 1 33.997 0.8090

First_person_corr 2.0234 1 33.884 0.1640

Perspective 2.2218 1 33.675 0.1454

Condition 0.0000 1 32.531 0.9992

P1 0.0693 1 33.286 0.7940

P2 1.0126 1 33.977 0.3214

Gender 0.4354 1 32.384 0.5140

LikeFiction 0.2119 1 32.819 0.6483

LikeFact 2.4934 1 33.727 0.1237

Perspective:Condition 0.5813 1 33.120 0.4512

Condition:LikeFiction 0.0081 1 33.821 0.9286

Condition:LikeFact 0.0874 1 33.990 0.7693

>

> fullmodelRTs3 = lmer (Third_person_RT ~ Third_person_corr + Perspective*Condition +

P1 + P2 + Gender + LikeFiction*Condition + LikeFact*Condition + (1|Story), data=cdata,

REML=TRUE)

> summary (fullmodelRTs3)

Linear mixed model fit by REML ['lmerMod']

Formula: Third_person_RT ~ Third_person_corr + Perspective * Condition +

P1 + P2 + Gender + LikeFiction * Condition + LikeFact * Condition + (1 | Story)

Data: cdata

REML criterion at convergence: 560.7

Scaled residuals:

Min 1Q Median 3Q Max

-2.0799 -0.5624 -0.1036 0.4767 2.5510

Random effects:

Groups Name Variance Std.Dev.

Story (Intercept) 13966 118.2

Residual 286230 535.0

Number of obs: 46, groups: Story, 4

Fixed effects:

Estimate Std. Error t value

(Intercept) -1110.111 1061.746 -1.046

Third_person_corr 93.964 121.097 0.776

Perspective3rd 19.910 228.686 0.087

ConditionFiction 2387.389 1288.675 1.853

P1 124.775 75.843 1.645

P2 81.797 67.166 1.218

Gender2 -79.131 197.798 -0.400

LikeFiction 88.680 94.545 0.938

LikeFact 283.977 146.398 1.940

Perspective3rd:ConditionFiction -192.426 365.759 -0.526

ConditionFiction:LikeFiction -374.095 143.598 -2.605

ConditionFiction:LikeFact 4.796 187.441 0.026

>

> PValues_RT1<- Anova(fullmodelRTs3, type = 3, test = 'F')

> PValues_RT1

Analysis of Deviance Table (Type III Wald F tests with Kenward-Roger df)

Response: Third_person_RT

F Df Df.res Pr(>F)

(Intercept) 1.0135 1 33.721 0.32123

Third_person_corr 0.4420 1 25.981 0.51204

Perspective 0.0075 1 31.488 0.93156

Condition 2.8674 1 32.543 0.09994 .

P1 2.5296 1 33.424 0.12115

P2 1.1828 1 31.163 0.28513

Gender 0.1555 1 32.099 0.69592

LikeFiction 0.8592 1 32.056 0.36088

LikeFact 3.4476 1 33.651 0.07212 .

Perspective:Condition 0.2561 1 33.478 0.61611

Condition:LikeFiction 6.1614 1 33.997 0.01816 *

Condition:LikeFact 0.0006 1 33.999 0.98097

---

Signif. codes: 0 ‘***’ 0.001 ‘**’ 0.01 ‘*’ 0.05 ‘.’ 0.1 ‘ ’ 1
