# Supplementary material for: When Fiction Is Just as Real as Fact: No Differences in Reading Behavior between Stories Believed to be Based on True or Fictional Events
Source: Front Psychol. 2017 Sep 20;8:1618. doi: 10.3389/fpsyg.2017.01618 (PMC5613255; doi:10.3389/fpsyg.2017.01618)

# S6: Results split by story

## 1st person perspective taking
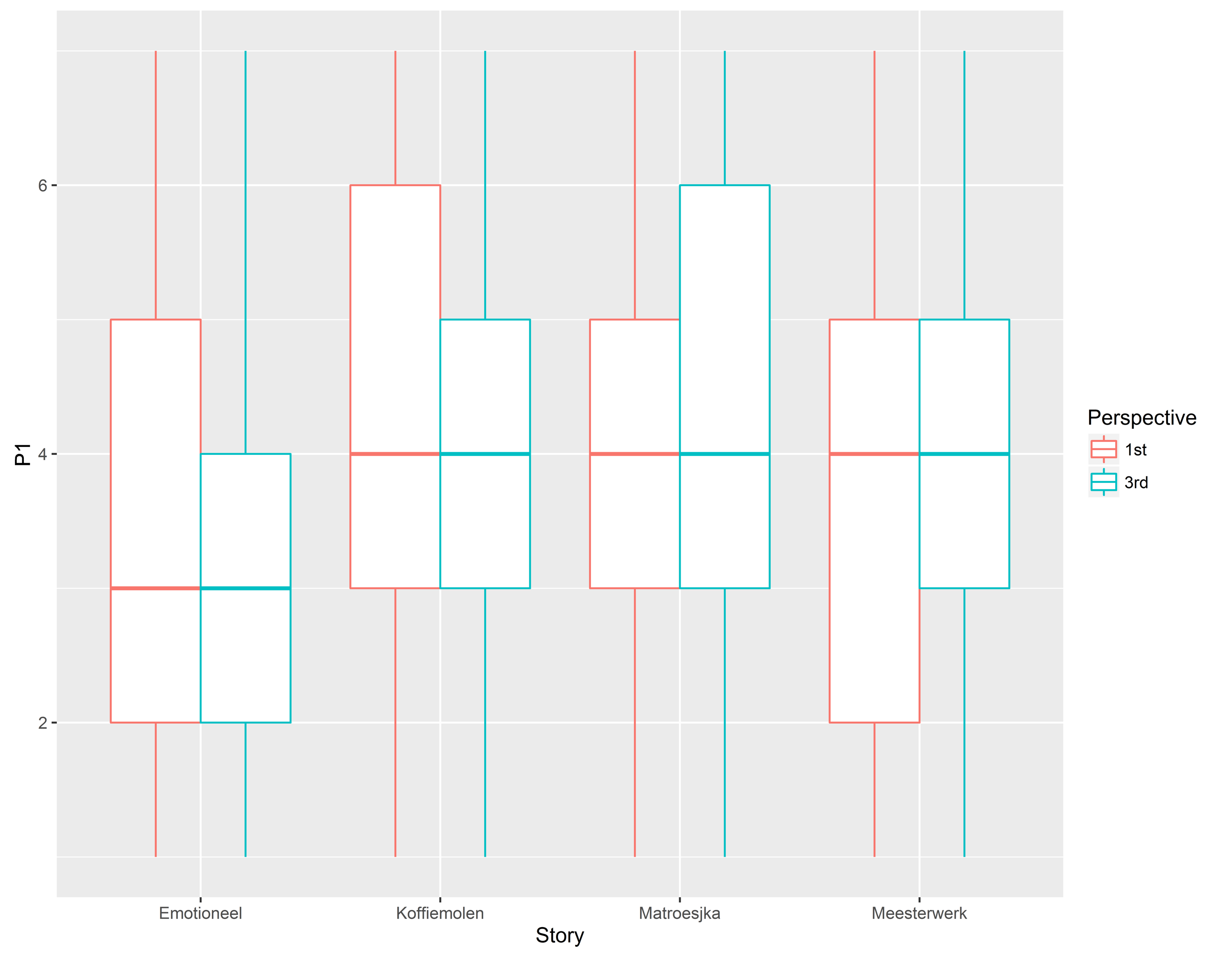


## 3rd person perspective taking
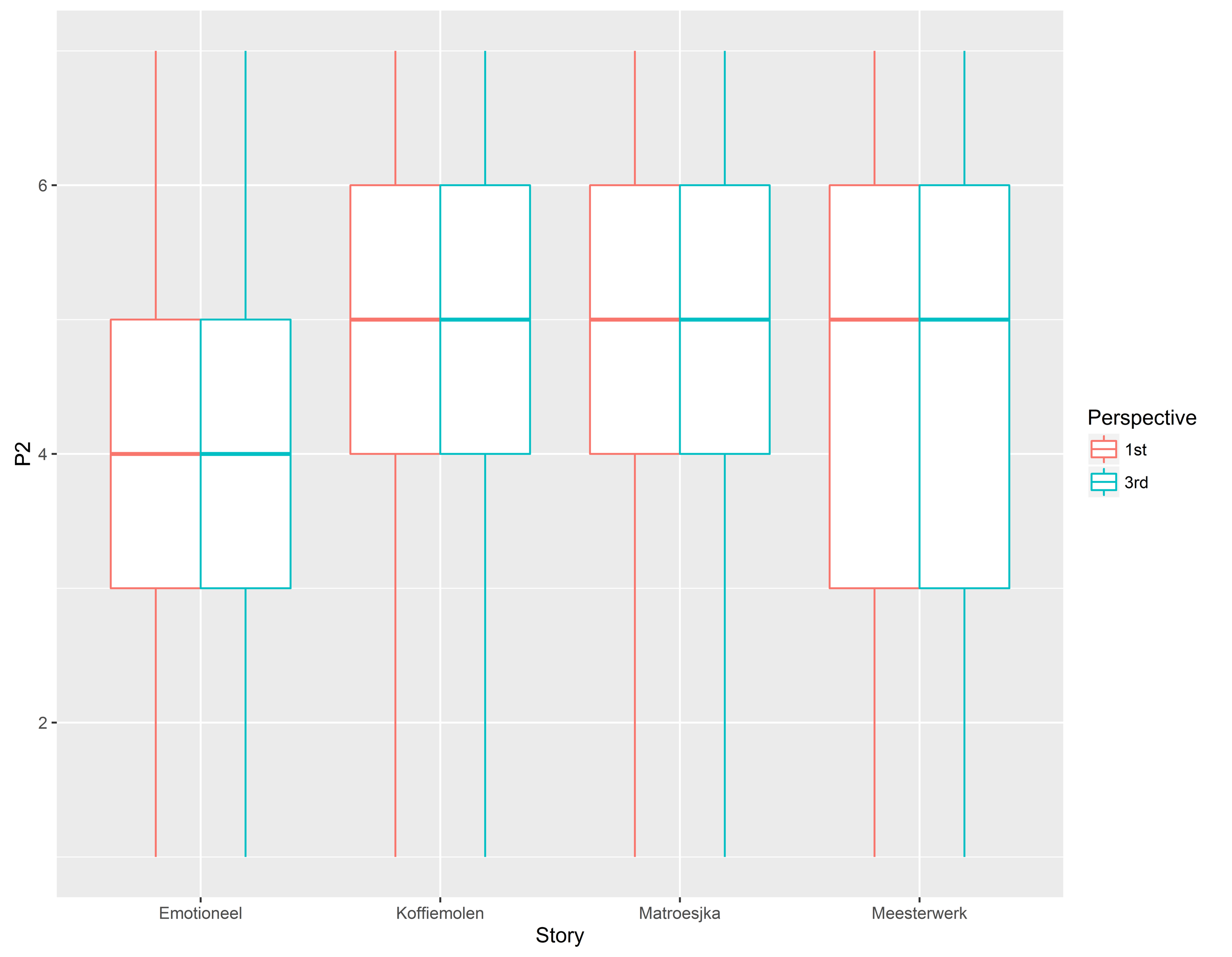


## Immersion subscales: Transportation


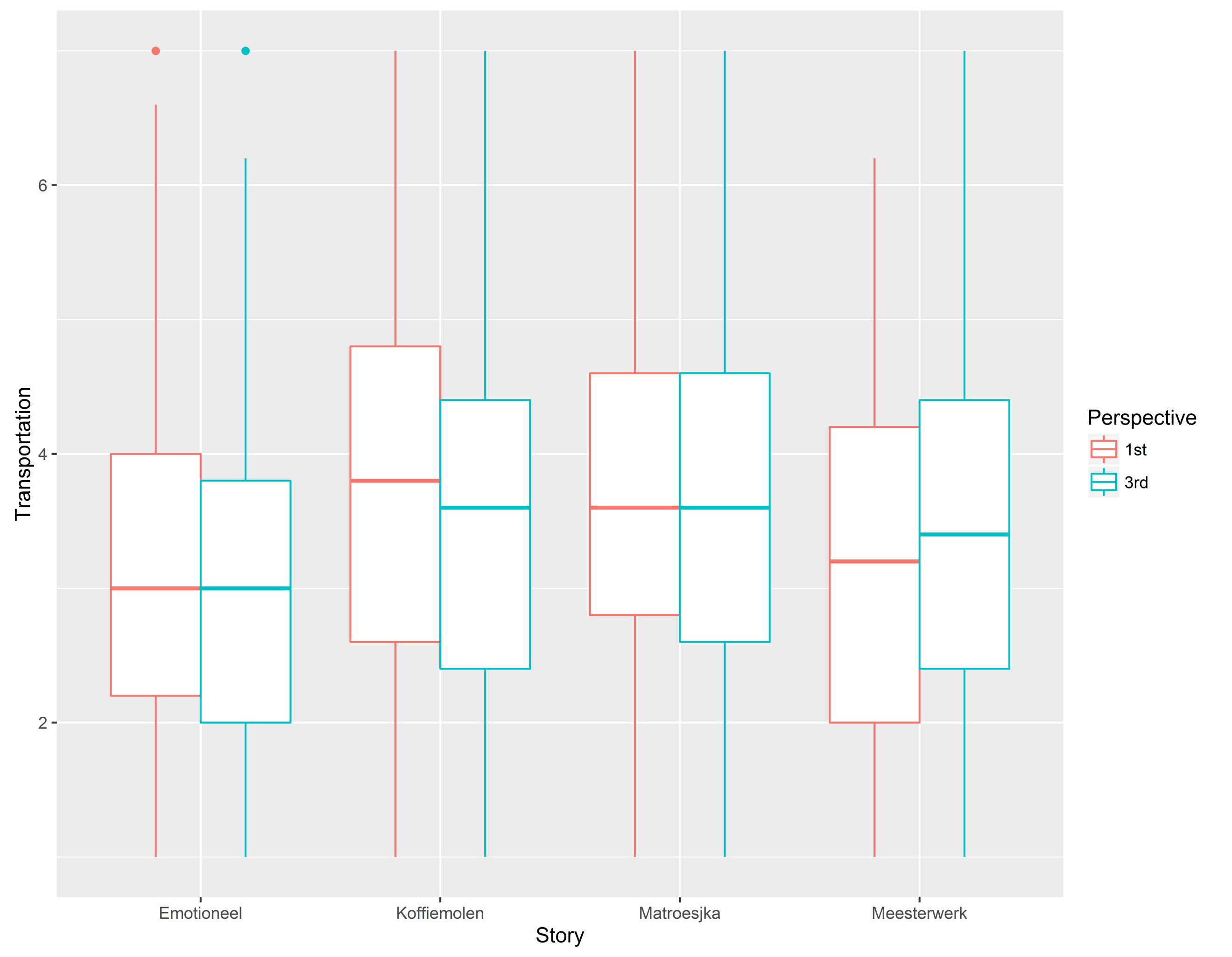


## Immersion subscales: Mental Imagery


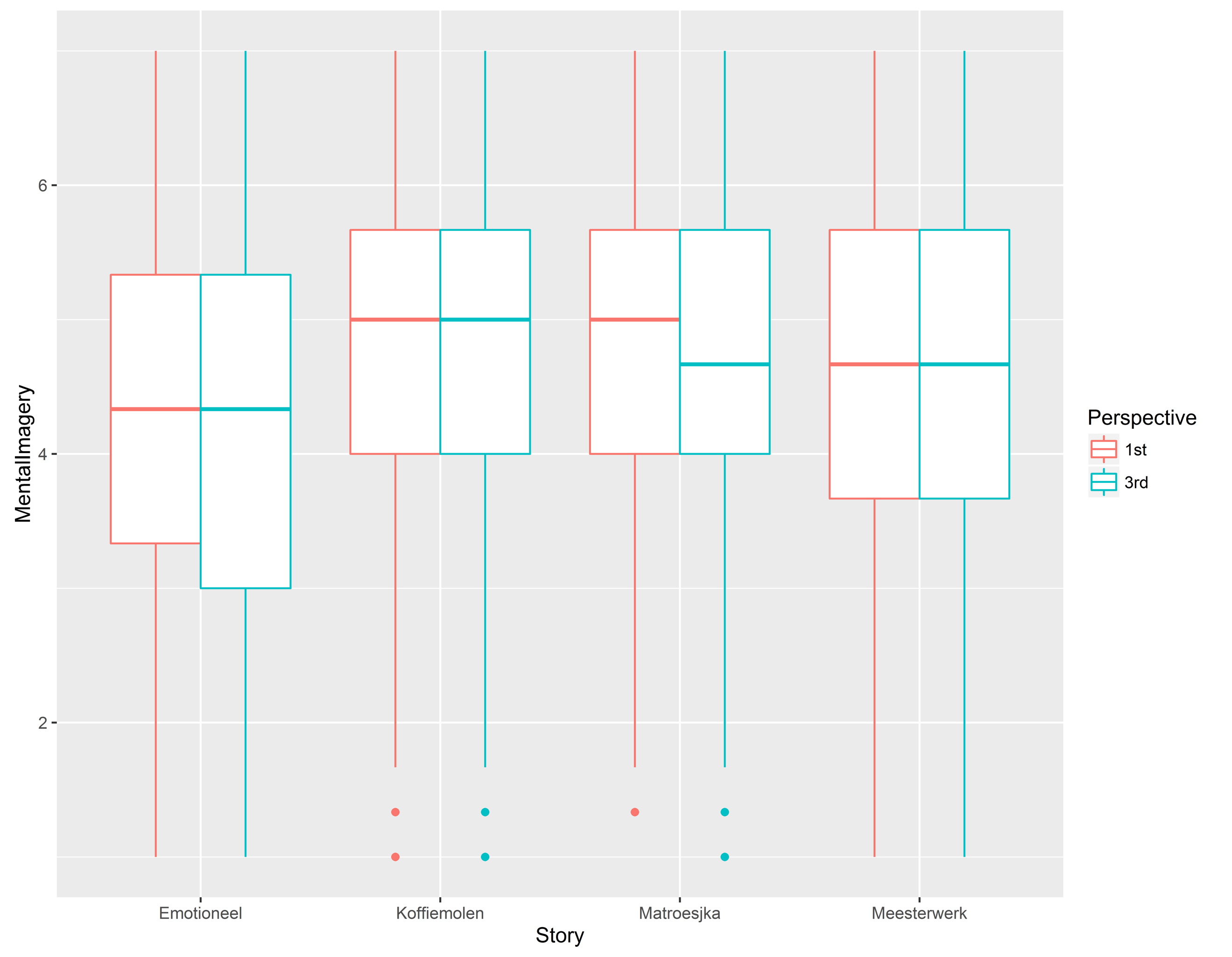


## Immersion subscales: Attention


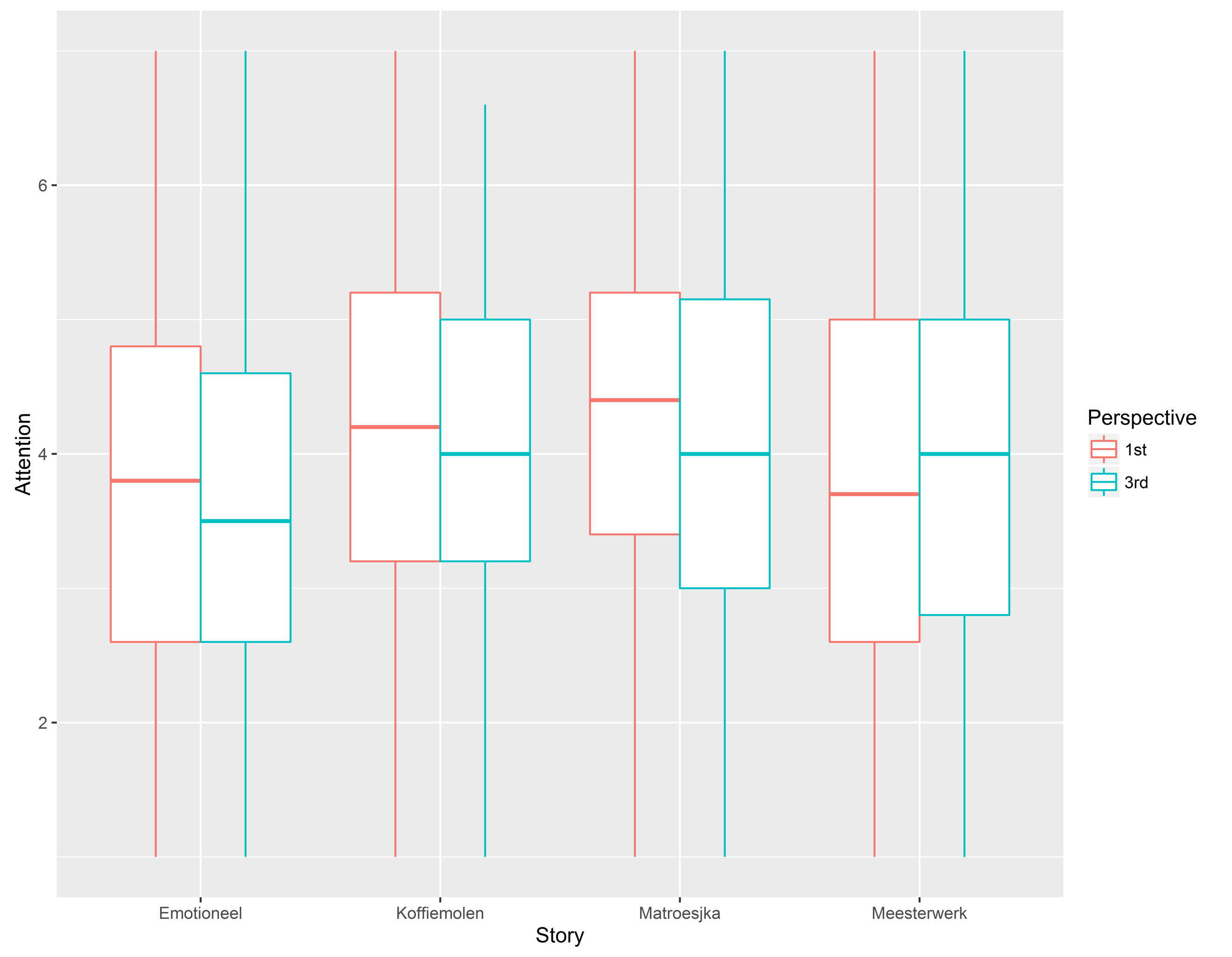


## Immersion subscales: Emotional Engagement with the Protagonist


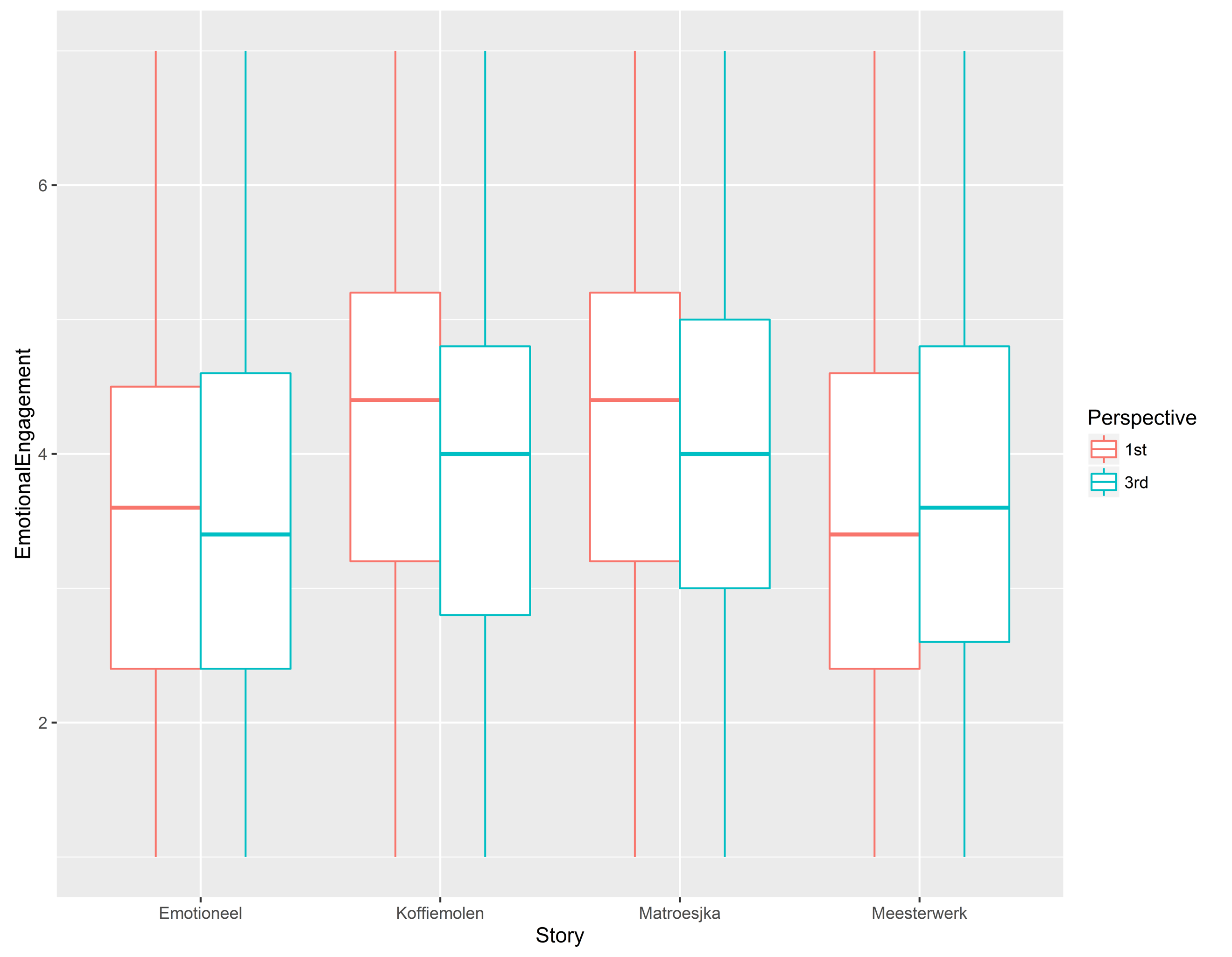

Supplement: Supplementary file 6 [file DataSheet6.DOCX]
