# Supplementary material for: When Fiction Is Just as Real as Fact: No Differences in Reading Behavior between Stories Believed to be Based on True or Fictional Events
Source: Front Psychol. 2017 Sep 20;8:1618. doi: 10.3389/fpsyg.2017.01618 (PMC5613255; doi:10.3389/fpsyg.2017.01618)
Supplement: Supplementary file 7 [file DataSheet7.DOCX]

# S7: Raw data

All data and experiment material is available via the following links. If you do not get immediately to the right directory, scroll down the archive and select the handle called ‘Fact or Fiction? (Project 55)’.

URL: https://corpus1.mpi.nl/media-archive/nbl_data/Frahar/Fact_or_fiction/Annotations/raw_behavioral_data_archive.zip

Handle URI: https://hdl.handle.net/1839/00-0000-0000-0022-58FF-E
